# The Conserved C-Terminus of the PcrA/UvrD Helicase Interacts Directly with RNA Polymerase

**DOI:** 10.1371/journal.pone.0078141

**Published:** 2013-10-16

**Authors:** Emma J. Gwynn, Abigail J. Smith, Colin P. Guy, Nigel J. Savery, Peter McGlynn, Mark S. Dillingham

**Affiliations:** 1 DNA:Protein Interactions Unit, School of Biochemistry, University of Bristol, Bristol, United Kingdom; 2 School of Medical Sciences, University of Aberdeen, Aberdeen, United Kingdom; 3 Department of Biology, University of York, York, United Kingdom; University of Iowa, United States of America

## Abstract

UvrD-like helicases play diverse roles in DNA replication, repair and recombination pathways. An emerging body of evidence suggests that their different cellular functions are directed by interactions with partner proteins that target unwinding activity to appropriate substrates. Recent studies in *E. coli* have shown that UvrD can act as an accessory replicative helicase that resolves conflicts between the replisome and transcription complexes, but the mechanism is not understood. Here we show that the UvrD homologue PcrA interacts physically with *B. subtilis* RNA polymerase, and that an equivalent interaction is conserved in *E. coli* where UvrD, but not the closely related helicase Rep, also interacts with RNA polymerase. The PcrA-RNAP interaction is direct and independent of nucleic acids or additional mediator proteins. A disordered but highly conserved C-terminal region of PcrA, which distinguishes PcrA/UvrD from otherwise related enzymes such as Rep, is both necessary and sufficient for interaction with RNA polymerase.

## Introduction

Helicases are ubiquitous, essential and abundant proteins that catalyse the separation of nucleic acids into their component single strands[1,2]. Based upon bio-informatics and structural analyses, helicases have been classified into several Superfamilies (SF) that also include proteins that do not display unwinding activity, but which display helicase-like architectures and catalyse related reactions such as ATP-dependent DNA translocation or nucleoprotein remodelling. UvrD-like helicases are a well-studied subfamily of the SF1 class whose members include the closely related enzymes UvrD and Rep from *E. coli*, and the PcrA protein from *Geobacillus stearothermophilus*. These three helicases have been used extensively as model systems for biochemical, biophysical and structural analysis of the DNA translocation and unwinding mechanism[3-5]. 

UvrD-like helicases share a conserved core motor region but display a broad range of cellular functions[5]. Indeed, it is not only the case that different UvrD-like enzymes perform different cellular roles. A single helicase protein is often implicated in several different DNA processing pathways, suggesting that these motors can in some manner be co-opted by the cell for a variety of tasks. There is emerging evidence that this functional specificity is conferred upon helicases by interaction with accessory proteins that target or modulate their activity for the task at hand[6]. This behaviour is exemplified by the multifunctional *E. coli* enzyme UvrD, which acts in nucleotide excision repair, mismatch repair, and the regulation of recombination[7-10]. It also has a role as the replicative helicase for rolling circle plasmids[11], and can assist genomic replication as a so-called “accessory helicase” that helps promote genome duplication through protein roadblocks such as the transcription apparatus[12,13]. Like UvrD, the highly homologous *E. coli* Rep helicase (37% identity) is involved in rolling circle replication pathways, but has also been shown to be recruited to the replisome to assist in genome duplication through interactions with DnaB[13]. This interaction requires the extreme C-terminus of Rep which, interestingly, is precisely the region that most clearly differentiates it from UvrD at the level of primary structure. The C-terminus of UvrD also appears to act as a protein interaction hub, having been shown to be important for interaction with UvrB, MutL and RecA[14-17]. As might be expected for such similar proteins, Rep and UvrD appear to have partially overlapping functions, at least one of which is essential as *rep* and *uvrD* mutations are synthetically lethal in rich growth medium. Because this lethality is suppressed by mutations that destabilise the interaction between RNAP and DNA, this essential function appears to be in overcoming blocks to replication presented by transcription complexes[13,18,19].

In contrast to the situation in *E. coli*, many bacteria do not contain two such similar helicases, but rather a single enzyme which is, on the basis of the distinctive C-terminal region discussed above, a UvrD homologue. For historical reasons this protein has been annotated PcrA (plasmid copy number reduction A) in many gram positive bacteria and this reflects its discovery as a protein that promotes plasmid rolling circle replication[20]. The PcrA protein has mainly been studied in an *in vivo* context in *Bacillus subtilis* and was shown to be involved in DNA repair and plasmid replication[21]. PcrA expression rescues the UV-sensitivity of an *E. coli uvrD* mutant indicating a probable role in nucleotide excision repair, which is additionally supported by biochemical studies[16]. Interestingly, *pcrA* mutants are hyper-recombinogenic, but this can be suppressed by mutations in the *recFOR* genes involved in initiating recombination at ssDNA gaps generated by replication defects[22]. Deletion of PcrA from *B. subtilis* renders the cells inviable[21] and PcrA expression restores the viability of the *uvrD rep* double mutant of *E. coli*[22]. Thus, the essential function of PcrA in *Bacillus subtilis* may be equivalent to the overlapping function of UvrD and Rep in *E. coli*. The PcrA helicase has been shown to interact physically and/or functionally with several proteins including the ‘beta propeller’ protein YxaL[23], the ribosomal protein L3[24], the plasmid replication initiator RepD[25], and most recently RNA polymerase (RNAP)[26,27]. In this work, we confirm the reported interaction between *Bacillus subtilis* PcrA and RNAP and demonstrate the conservation of an equivalent interaction in *E. coli*, where UvrD but not Rep interacts with RNAP. Furthermore, we show that this interaction is direct and dependent upon the extreme C-terminal extension of PcrA. The helicase activity of PcrA is stimulated by RNAP *in vitro* but, surprisingly, this does not require the C-terminal interaction domain. 

## Materials and Methods

### Protein preparations

*Geobacillus stearothermophilus* PcrA (GstPcrA) was purified as described previously[24], but with the following modification. Instead of running a low substitution blue sepharose column, after gel filtration the protein was loaded on a mono Q column at room temperature and eluted over a gradient between 100 mM and 1 M NaCl. Peak fractions were dialysed into storage buffer (50 mM Tris pH 7.5, 2 mM EDTA, 1 mM DTT, 200 mM NaCl and 10% glycerol). Non-biotinylated *Escherichia coli* UvrD and Rep helicases were purified essentially as described previously[16,28].

Biotinylated GstPcrA was expressed from a pET22b vector (Novagen) containing full length wild type PcrA with a 20 amino acid tag at the N-terminus (MSG LND IFE AQK IEW HEG GG). The lysine is a target for *in vivo* biotinylation by the *E. coli* BirA enzyme. BL21(DE3) cells (Novagen) were transformed with the modified plasmid pET22b-^bio^PcrA and pBirACm (Avidity) and grown to mid-log phase at 37 °C in LB, supplemented with the appropriate antibiotics. IPTG and biotin were added to 1 mM and 50 μM respectively, and the cells were grown for a further three hours at 37 °C. Cells were sonicated and ammonium sulfate (50% saturation) was added to the soluble extract. The precipitated material was recovered and biotinylated proteins were isolated by affinity chromatography using Softlink Avidin resin (Promega), according to the manufacturer’s instructions. HiTrap heparin chromatography (GE Healthcare), dialysis, storage and quantification of biotag-PcrA were then carried out essentially as described previously for the native protein[24]. All other bio-tagged helicases were expressed using analogous vector constructs and with an equivalent purification procedure. The concentrations of biotinylated *Geobacillus stearothermophilus* PcrA (GstPcrA), *Geobacillus* *sp*. PcrA (GspPcrA), *Bacillus subtilis* PcrA (BsuPcrA), *Escherichia coli* UvrD (EcUvrD) and *Escherichia coli* Rep (EcRep) were determined using theoretical extinction co-efficients of 81660, 81250, 75750, 111270 and 82280 M^-1^cm^-1^ respectively.

Biotinylated GstPcrA with a C-terminal deletion (PcrAΔC) was produced by site-directed mutagenesis (QuikChangeII kit, Stratagene) of the pET22b-^bio^PcrA vector to introduce a stop codon at amino acid position 653. The protein was expressed and purified in the same manner as the full length protein. The concentration of PcrAΔC was determined by spectrophotometry using a theoretical extinction co-efficient of 70250 M^-1^cm^-1^. The biotinylated C-terminus of *G. stearothermophilus* PcrA (PcrA-Ct) was produced by deleting the entire N-terminal region of the *pcrA* gene from the pET22b-^bio^PcrA vector such that the biotinylation tag was fused directly to the final 72 amino-acids of the protein (residues 653-724). The protein was purified using Softlink Avidin resin (Promega), according to the manufacturer’s instructions. Peak fractions were pooled and dialysed against a buffer containing 50 mM Tris pH 7.5, 2 mM EDTA, 1 mM DTT and 100 mM NaCl. The protein was applied to a 1 ml monoQ column (GE Healthcare) and the flowthrough collected and dialysed extensively against storage buffer (50 mM Tris pH 7.5, 2 mM EDTA, 1 mM DTT, 200 mM NaCl and 10% glycerol). The concentration of PcrA-Ct was determined by spectrophotometry using a theoretical extinction co-efficient of 16500 M^-1^cm^-1^.

For purification of *B. subtilis* RNA polymerase, *B. subtilis* MH5636 cells were employed[29]. This strain contains a C-terminal histidine tag on a genomically-encoded RNA polymerase β' subunit. Cells were grown to an OD_600_ of 1.2 in 4 x LB, in a 20 litre bioreactor (Applikon biotechnology). Harvested cells were resuspended in 10 ml resuspension buffer (10% sucrose, 20 mM Tris pH 7.5, 1 mM DTT & 100 mM NaCl + 5 mM Imidazole) per litre of the original media, lysed by sonication and clarified by centrifugation. The supernatant was run over a 5 ml HisTrap Nickel column (GE Healthcare), and step gradients of buffer A (20 mM Tris pH 7.5, 300 mM NaCl, 5% glycerol + 0.1 mM DTT) + 20 mM Imidazole and buffer A + 200 mM Imidazole were applied. The 200 mM imidazole peak fraction was dialysed against buffer B (10 mM Tris pH 7.5, 5% glycerol, 0.1 mM EDTA, 0.1 mM DTT) + 50 mM NaCl. The protein was then passed over a HiTrap heparin column (GE Healthcare) and eluted with a gradient of 50 mM NaCl to 800 mM NaCl. Fractions containing predominantly core RNAP were identified by coomassie-stained SDS-PAGE, pooled and dialysed into storage buffer (10 mM Tris pH 8.0, 150 mM NaCl, 0.1 mM EDTA, 0.1 mM DTT & 10% glycerol). The concentration of RNAP was determined using a Bradford assay (BioRad) according to manufacturer’s instructions and with BSA as a standard. *Escherichia coli* RNAP was purified as described previously[30].

### Cell lysate preparation

Cell lysate was prepared from a strain annotated *Geobacillus* *sp.* (DRM 13240, formerly annotated *Geobacillus stearothermophilus*), *B. subtilis* 168, and *E. coli* MG1655 by shaking cells at 60 °C, 30 °C, or 37 °C respectively and harvesting at an OD_600_ of 1.2 absorbance units. Cells were harvested by centrifugation, resuspended in 5 ml resuspension buffer (10% sucrose, 20 mM Tris pH 7.5, 1 mM EDTA, 1 mM DTT & 100 mM NaCl) per litre of original media, and lysed by sonication. The lysate was clarified by centrifugation and diluted to a total protein concentration of ~14 mg/ml. Where indicated in the text and figure legends, the lysate was depleted of nucleic acids by treatment with 5 mM MgCl_2_, 10 µg/µl DNaseI and 10 µg/µl RNaseA for 15 minutes at room temperature before use in pull down experiments. The apparently complete depletion of nucleic acids from the cell lysate was confirmed by agarose gel electrophoresis with ethidium bromide staining.

### Pull down experiments

Pull down experiments were carried out using streptavidin magnetic beads (New England Biolabs). Each addition or washing step was performed at 4 °C by placing Eppendorf tubes containing beads on a rotator device, designed to continually but gently mix the beads with the sample. Tubes were then placed on a magnetic stand for two minutes and excess solution was removed. In each pull down experiment, 100 µl of the bead preparation was washed with 200 µl pull down buffer (20 mM Tris pH 7.5, 150 mM NaCl and 1 mM EDTA) for 5 minutes, and then with 200 µl of biotinylated protein for 20 minutes to enable protein to bind to the beads. Biotinylated bait protein was used at 1 µM unless otherwise stated, and diluted from stocks in pull down buffer + 0.1% BSA. The beads were then washed with 200 µl pull down buffer + 0.1% BSA for 5 minutes before mixing with 200 µl of cell lysate for 20 minutes. Two subsequent 5 minute wash steps with pull down buffer were carried out, the first of which included 0.1% BSA. Beads were finally boiled for 5 minutes in 50 µl SDS loading buffer (62.5 mM Tris-HCl pH 6.8, 10% SDS, 25% glycerol, 0.7 M β-mercaptoethanol, 0.5% bromophenol blue). Following removal of the magnetic beads, the samples were loaded onto SDS-PAGE gels for visualization of bands. The identities of proteins in the bands of interest were confirmed by either N-terminal sequencing and mass spectrometry (both conducted at the Proteomics Facility, University of Bristol; see File **S1** for further methodological details and complete mass spectrometry datasets) or by probing with an antibody against the β subunit of *E. coli* RNAP.

### Surface Plasmon Resonance

Surface plasmon resonance was performed at 25 °C using a Biacore 2000 instrument. Biotinylated GstPcrA, BsuPcrA, BsuAddA, EcRecB, EcUvrD and EcRep were diluted in 10 mM Hepes pH7.4, 150 mM NaCl, 3 mM EDTA and 0.005% P20. Approximately 7000 resonance units of each protein were immobilised on the flow cell of a streptavidin chip. On each chip one flow cell did not contain immobilised protein, to act as a control for non-specific interactions to the dextran matrix. Following immobilisation, six concentrations of BsuRNAP (10, 20, 50, 100, 200 and 500 nM), in the same buffer, were flowed across each flow cell at 10 µl / min and binding was monitored. Data for binding to the blank flow cell was subtracted from each data set to correct for non-specific interactions. The experiment was also carried out in reverse, with approximately 3000 resonance units of BsuRNAP immobilised on a CM5 chip using EDC/NHS amine coupling in 10 mM Sodium Acetate, pH 4.9. 1 µM, 2 µM and 5 µM of native (non-biotinylated) GstPcrA, EcUvrD and EcRep were prepared in 10 mM Hepes pH7.4, 150 mM NaCl, 3 mM EDTA and 0.005% P20, flowed across each flow cell and binding was monitored. Data for binding to the blank flow cell was subtracted from each data set.

### Helicase assays

Substrates for helicase assays were prepared by radioactively labelling the 5′ ends of synthetic oligonucleotides using T4 polynucleotide kinase and γ-^32^ATP and annealing to a second strand in a 1:1 ratio. To make the 3′ tailed substrate, a labeled 30 base oligonucleotide (5′-CAA TAC GCA AAC CGC CTC TCC CCG CGC GTT) was annealed to a 60 base oligonucleotide (5′-AAC GCG CGG GGA GAG GCG GTT TGC GTA TTG GGC GCT CTT CCG CTT CCT CGC TCA CTG ACT-3′) to create a substrate with a 30 base pair duplex and a 30 base single stranded 3′-tail. Excess γ-^32^ATP was removed by passing the sample through a G-25 Microspin column (GE Healthcare). Helicase assays were performed essentially as described[31] using 50 nM helicase and 100 nM *B. subtilis* RNAP unless indicated otherwise. Assays on 3′-tailed substrates were carried out at 20 °C, in a buffer containing 25 mM Tris Acetate pH 7.5, 2 mM Magnesium Acetate, 0.5 mM DTT and 50 mM NaCl. Proteins were added and incubated together on ice for 5 minutes before addition of 1 nM DNA and a further incubation at 20 °C for 5 minutes. Reactions were then started by the addition of 0.5 mM ATP. Reactions were stopped by mixing with an equal volume of stop buffer (200 mM EDTA, 1% SDS, 10% Ficoll 400, 0.125% bromophenol blue, 0.125% xylene cyanol and 100 nM unlabelled 30mer oligonucleotide). The products were run on 8% acrylamide TBE gels at 120V, dried onto DEAE paper and visualised using a Typhoon 9400 phosphorimager (Molecular Dynamics). Gels were quantified using Imagequant software version 3.3 (Molecular Dynamics) to determine values for the percentage of DNA unwound that were corrected for the presence of unwound DNA in the “no protein” control.

## Results

### PcrA helicase interacts directly with RNA polymerase

**Figure 1:**
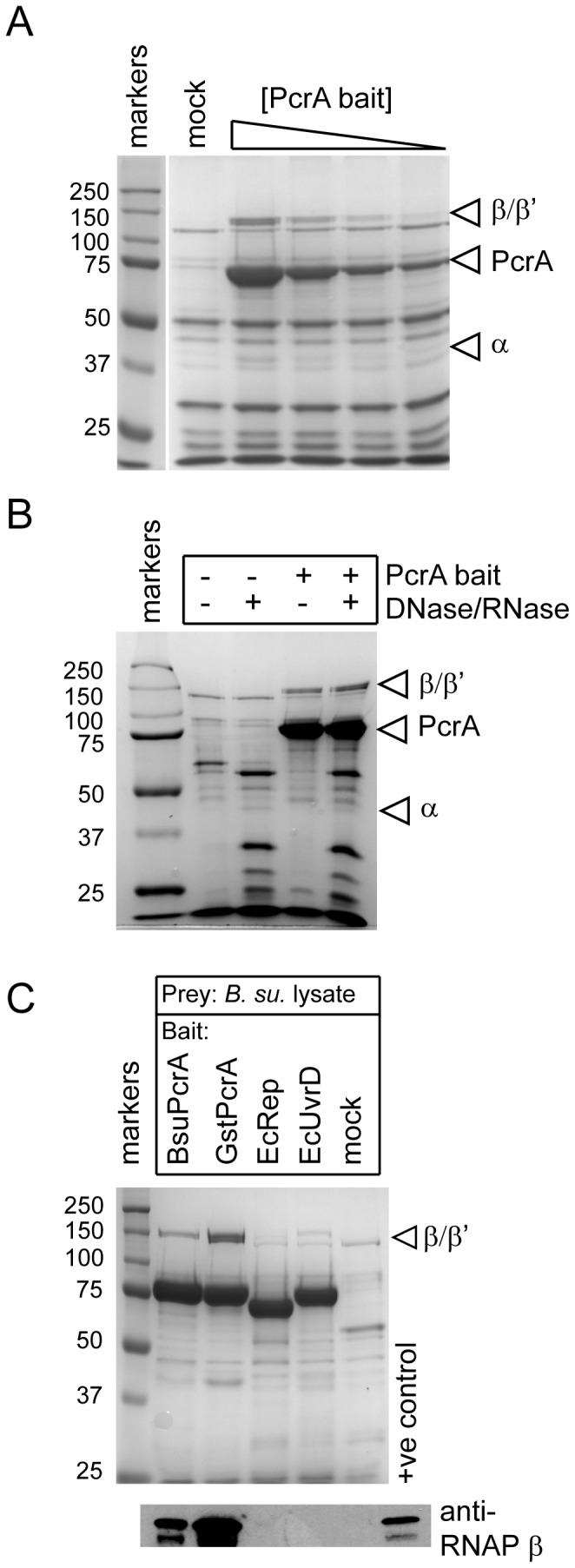
Interactions between PcrA helicase and RNAP studied using magnetic bead pull down assays. (A) SDS-PAGE gel showing dose-dependent pull down of the core RNAP subunits from a DNA/RNA-depleted *B. subtilis* cell extract by biotinylated BsuPcrA (between 2 - 17 µg) on magnetic streptavidin beads. In the mock pull down, the beads were not baited with PcrA. (B) Depletion of nucleic acids from the cell extract by treatment with DNase and RNase has no apparent effect on the efficiency of RNAP pull down by BsuPcrA. (C) Interaction with *Bacillus* RNAP is specific to the PcrA helicase. The beads were baited with the helicase indicated and used to pull down proteins from *B. subtilis* cell extract (upper panel). The samples were analysed for the presence of RNA polymerase using an antibody against the β subunit (lower panel). The positive control lane contains purified RNA polymerase.

To probe for protein interaction partners of PcrA and related enzymes, we purified biotinylated derivatives of several SF1A UvrD-like helicases with an N-terminal tag that is a target for *in vivo* modification by biotin ligase. The resulting biotin-tagged helicases were highly pure and shown to be active unwinding enzymes in helicase assays (Figure S1 in File **S1**). They were then used to bait streptavidin-coated magnetic beads for use in pull down experiments with whole cell extracts of *Bacillus subtilis, Geobacillus* *sp.* or *Escherichia coli*. The baited beads were incubated with the cell extract to allow interaction with prey proteins, before extensive washing with buffer, followed by SDS-PAGE analysis of the material retained on the beads. An experiment using biotinylated *B. subtilis* PcrA as bait in a *B. subtilis* cell extract revealed a dose-dependent pull down of three prominent bands at approximate molecular weights of 140 kDa, 140 kDa and 40 kDa, which are consistent with the β, β' and α subunits of RNAP respectively (Figure **1A** and Figure S2 in File **S1**). A mock pull down with no bait revealed non-specific retention of various unknown proteins on the magnetic beads but these were not enriched by the presence of increasing concentrations of bait. N-terminal sequencing (data not shown) and mass spectroscopy (see below) subsequently confirmed that the material retained on the beads in a PcrA-dependent fashion was indeed RNAP. To test for the possibility that the apparent interaction between PcrA and RNAP was mediated by nucleic acids, we performed the pull down experiment in the presence or absence of a combination of DNase1 and RNaseA or with the pan-nuclease benzonase (Figure **1B** and data not shown). Nucleic acid depletion of the cell extract had no discernable effect on the quantity of RNAP retained on the beads. Furthermore, we purified a mutant biotinylated GstPcrA protein with an alanine substitution at a conserved tryptophan residue (W259), which is known to dramatically decrease the affinity of PcrA for ssDNA and this was equally efficient at pulling down RNAP from the cell extract, even when it had also been depleted of nucleic acids (data not shown).

To test the specificity of the PcrA-RNAP interaction we next compared the ability of PcrA and related helicases to pull down RNAP from a *B. subtilis* cell extract (Figure **1C**). The well-characterised PcrA protein from the moderate thermophile *Geobacillus stearothermophilus*, which is 70% identical to the *B. subtilis* enzyme, showed similar interactions with RNAP. Note that, since biotinylated and unmodified *G. stearothermophilus* PcrA were both available in considerably larger yields than the equivalent enzymes from *B. subtilis*, they are used in several of the experiments presented in the manuscript in place of the cognate *B. subtilis* helicase. In contrast, the *E. coli* helicases Rep and UvrD were not able to interact with *Bacillus subtilis* RNAP at the level of detection of our assay. Similar experiments were performed using a cell extract from a *Geobacillus* species. These showed a clear preferential pull down of the RNAP by *G. stearothermophilus* PcrA, and weaker interactions with *B. subtilis* PcrA and *E. coli* UvrD (Figure S3 in File **S1**).

**Figure 2:**
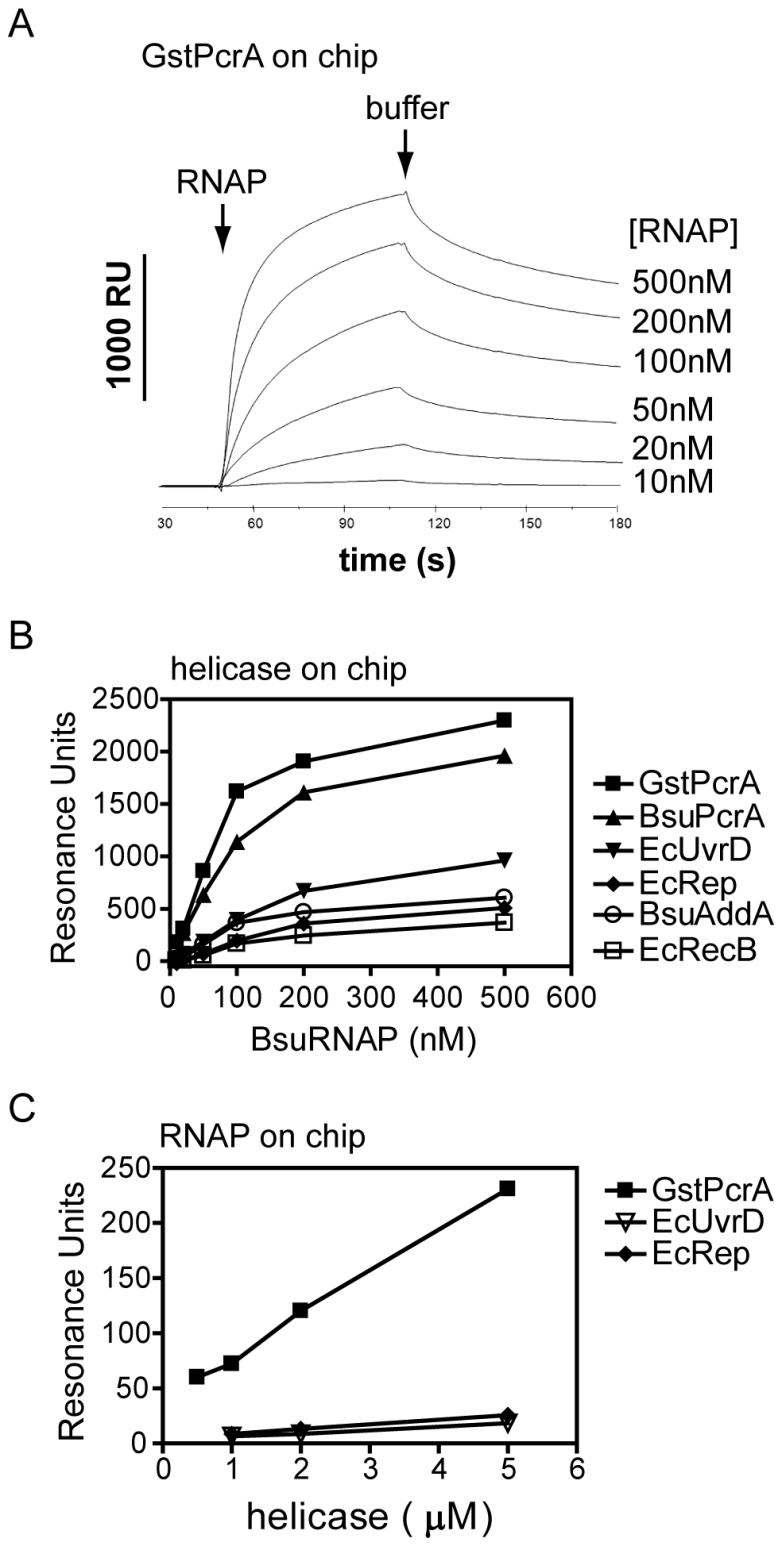
PcrA helicase interacts directly with purified RNA polymerase. Protein-protein interactions were monitored using surface plasmon resonance as described in the Methods section. (A) Example sensorgrams for the binding of BsuRNAP to immobilized GstPcrA. (B) Different biotinylated helicases were immobilized in a flow cell containing a streptavidin-coated sensor chip. Increasing concentrations of BsuRNAP were flowed over and binding was monitored as an increase in resonance units. The graph shows the collated results for binding of BsuRNAP to all immobilized helicases. C) his-tagged BsuRNAP was amine-coupled to a CM5 chip and the GstPcrA, EcUvrD and EcRep helicases were subsequently flowed over at different concentrations. Binding was monitored as an increase in resonance units.

These experiments suggest that the interaction between PcrA and RNAP is specific, and that it does not require the binding of nucleic acids to either protein, but do not exclude the possibility that other proteins or small molecules in the cell extract are required. To test for direct interactions between purified proteins we employed surface plasmon resonance to monitor the binding of purified core *B. subtilis* RNAP to PcrA (Figure **2**). This approach was used because pull down experiments using purified proteins were not tractable due to substantial non-specific binding of the RNAP to the magnetic beads. PcrA and other helicases were immobilised on a streptavidin-coated chip, and the resonance units recorded as RNAP was flowed over and then washed off the chip at various concentrations. The signals were corrected for the amount of RNAP binding to an unmodified control channel on the flow cell chip. RNAP was retained on the chip in a concentration-dependent manner and to a similar extent when either *G. stearothermophilus* or *B. subtilis* PcrA were immobilised (Figures **2A and 2B**). Replacement of the PcrA helicase with the *E. coli* helicases UvrD or Rep resulted in a much weaker retention of RNAP (Figure **2B**). Indeed, these signals were comparable to those obtained with immobilised *B. subtilis* AddA and *E. coli* RecB helicases. Those enzymes function within multiprotein complexes to process double-stranded DNA breaks for repair by recombination and they do not interact with RNAP in the cell[32]. Therefore, these weaker signals may well represent a background level of binding to immobilised proteins, albeit above the signal for the unmodified chip control channel. To control for artefacts associated with surface immobilisation and surface interactions, we switched around the bait and prey in our experiments by non-specific immobilisation of the RNAP onto the chip via amino-coupling chemistry, and then flowing over different helicases. The immobilised RNAP showed a clear preference for binding *G. stearothermophilus* PcrA in comparison to *E. coli* UvrD and Rep (Figure **2C**). These experiments are in broad agreement with the pull down studies presented above and show that the interaction of PcrA with RNAP does not require additional mediator proteins or nucleic acids.

As further confirmation of the interaction, we employed mass spectroscopy to compare proteins present in gel slices from the BsuPcrA pull down experiment with those from a mock pull down control. The results are summarised in Table **1**, which lists proteins detected with highest confidence in the pull down experiment that also displayed at least five-fold enrichment over the control experiment (see Table **S1** in File **S1**). As would be expected, the PcrA bait itself is detected as the most abundant and enriched protein in the pull down. All three large subunits of RNA polymerase are enriched by between 5- and 7-fold compared to the control. Interestingly, we also detected the helicases YvgS (HelD), which was shown previously by others to interact with RNAP[26] and whose expression level has been used as a biomarker for the inhibition of transcription by antibiotics[33] and YwqA, which is a homologue of the RNAP interaction partner RapA/HepA. The most enriched protein in this list is another SF2 helicase, the nucleotide excision repair protein UvrB. UvrB is a known interaction partner of UvrD in the *E. coli* nucleotide excision repair system[16,28], and PcrA is probably also involved in this pathway[21], consonant with a PcrA-UvrB interaction. A range of sigma factors are present (see File **S1**) suggesting that PcrA is not interacting with a specific RNAP-sigma variant, although SigB (a general stress response factor) is the most common. Noteworthy absentees from the list of possible interaction partners include the RecA, YxaL and rpL3 proteins. These are all thought to interact with PcrA, but were either undetected above the threshold confidence value of the experimental pull down, or with lower confidence scores in the pull down compared to the mock control experiment.

**Table 1:** Detection by mass spectroscopy of proteins pulled down from *Bacillus subtilis* soluble cell extracts using biotinylated BsuPcrA helicase as bait.

Protein detected (order of confidence)	Enrichment over control (fold)
PcrA	31
RpoC	5
RpoB	7
RpoA	6
YvgS	*
SigB	*
YwqA	19
UvrB	24

The table shows the eight most confident hits in the pulldown (listed by highest confidence first) which were also present at greater than five-fold enrichment compared to a “no bait” control experiment. The complete data set and further details are available in Table S1 in File **S1**. The protein names used here are those given in the Subtilist database[62]. * YvgS and SigB were not detected in the mock pull down control experiment above a threshold confidence value.

### UvrD helicase interacts with RNA polymerase from *E. coli*

**Figure 3:**
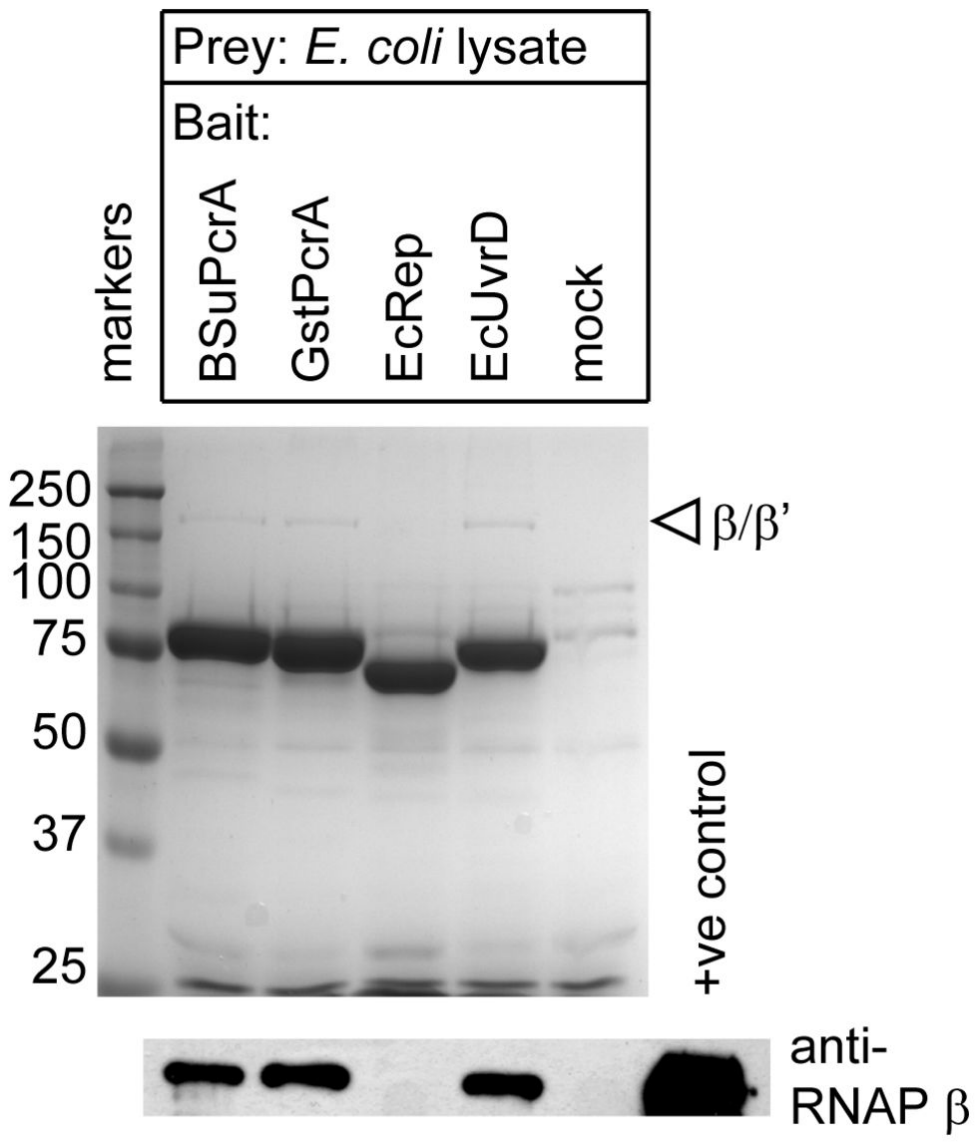
Interaction between *Escherichia coli* UvrD helicase and RNAP. Pull down experiments were performed as described in the methods using the indicated biotinylated helicase as bait and *E. coli* cell extract as the prey. Proteins retained on streptavidin magnetic beads were compared against a mock control experiment in which the beads were not baited (upper panel). The samples were also analysed by western blot for the presence of RNAP using an antibody against the β subunit (lower panel). The positive control lane contains purified RNA polymerase.

To investigate whether an equivalent interaction between a SF1 helicase and RNAP was conserved in *E. coli* we performed equivalent pull down experiments to those presented above but using *E. coli* cell extracts. *E. coli* UvrD was able to pull down RNAP whereas the closely related Rep helicase could not (Figure **3**). Interestingly, both PcrA proteins tested were able to interact with *E. coli* RNAP in these assays. Taken together, our experiments suggest that the interaction with RNAP is specific to the PcrA and UvrD helicases.

### A conserved C-terminal extension of PcrA is necessary and sufficient for interaction with RNA polymerase

**Figure 4:**
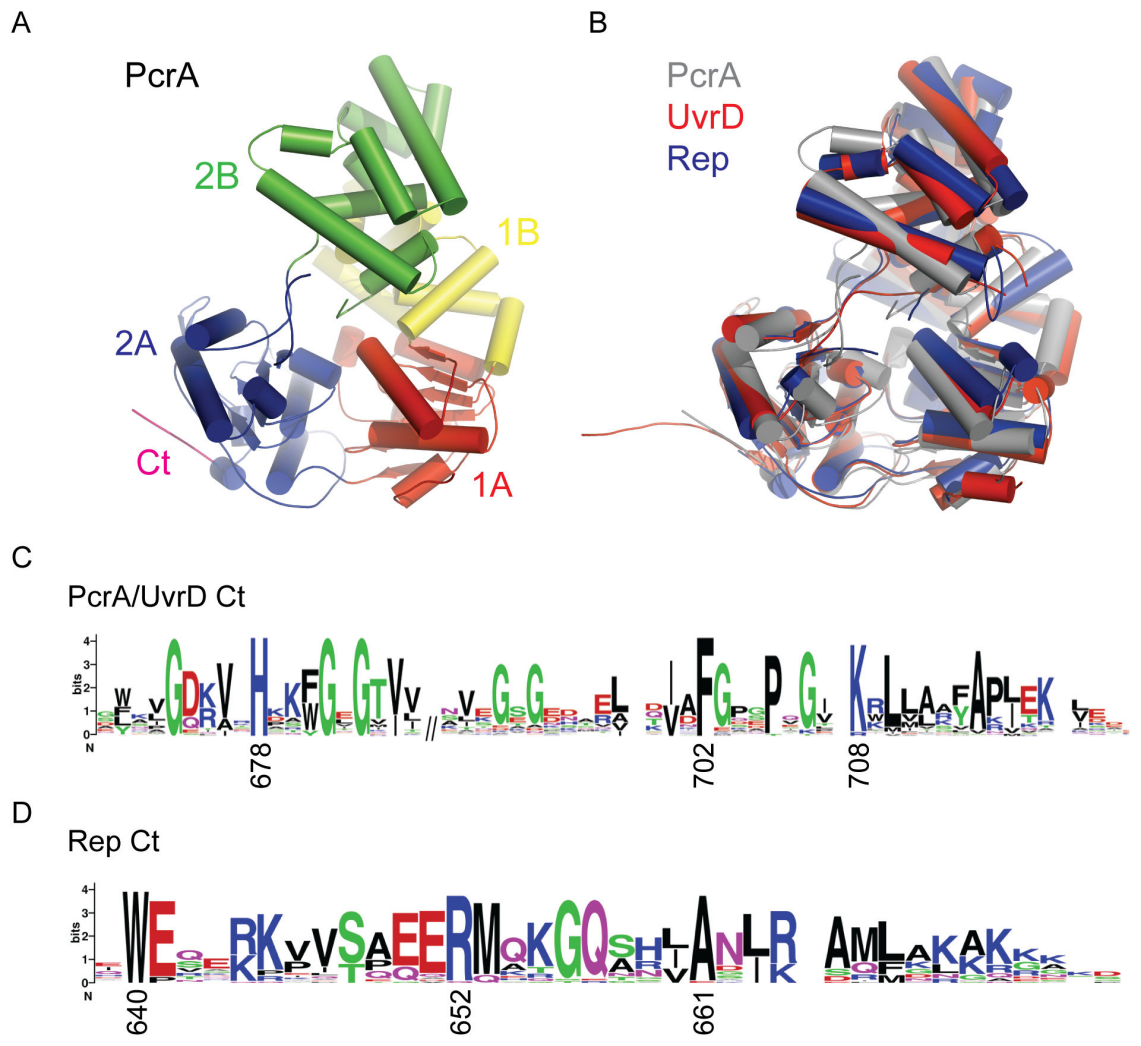
Comparison of the structures and C-terminal amino acid sequence conservation in UvrD-like helicases. The helicases shown are *G. stearothermophilus* PcrA (PDB:3PJR, [34]), *E. coli* UvrD (PDB:2IS1, [39]) and *E. coli* Rep (PDB:1UAA, [58]). (A) Subdomain structure of the PcrA helicase. The majority of the C-terminal domain (magenta) is disordered in all crystal structures of PcrA and closely related helicases. (B) PcrA, UvrD and Rep share very similar overall folds but the extreme C-terminus of each protein is disordered. (C) For the PcrA and UvrD helicases the disordered C-terminal region is a stretch of ~90 amino acids, of which the final ~50 are well conserved across diverse bacterial species. The weblogo[59] motif shows the sequence conservation and was created from a multiple alignment of 250 PcrA/UvrD sequences implemented in COBALT[60] using a representative protein set[61]. Reference numbering is shown at highly conserved residues for *E. coli* UvrD. (C) For Rep helicase, the C-terminal region is ~40 residues in length and is conserved in γ proteobacteria. This weblogo was created with 37 Rep sequences from a representative protein set. Reference numbering is shown at highly conserved residues for *E. coli* Rep.

Numerous crystal structures of UvrD-like helicases (including PcrA, Rep and UvrD) have been solved (Figures **4A and 4B**). They share a similar four subdomain architecture, including the very highly conserved core region consisting of two RecA-like folds in tandem repeat (subdomains 1A and 2A). This core region is found in all SF1 and SF2 helicases and translocases and is responsible for coupling ATP hydrolysis to directional motion on DNA or RNA[2]. More variable accessory domains (1B and 2B) are found as inserts within the core domains and it has been argued that these are likely to act as sites for interaction with partner proteins[3,6]. Interestingly, whereas UvrD is a close sequence homologue of PcrA for the entire length of both proteins, the similarity of Rep and PcrA is limited to the large N-terminal region of the proteins which includes the four subdomains and which define the canonical Superfamily I helicase. This is because PcrA and UvrD share homologous C-terminal extensions whereas the equivalent region of Rep is distinctive and involved in protein interactions with the replicative helicase DnaB (Figure **4C**). In every crystal structure of PcrA, UvrD and Rep, this C-terminal extension is disordered. Given the specificity of the RNAP interaction observed for PcrA/UvrD versus Rep, the primary structures suggest that the C-terminal extension of PcrA might be the site of interaction with RNAP.

**Figure 5:**
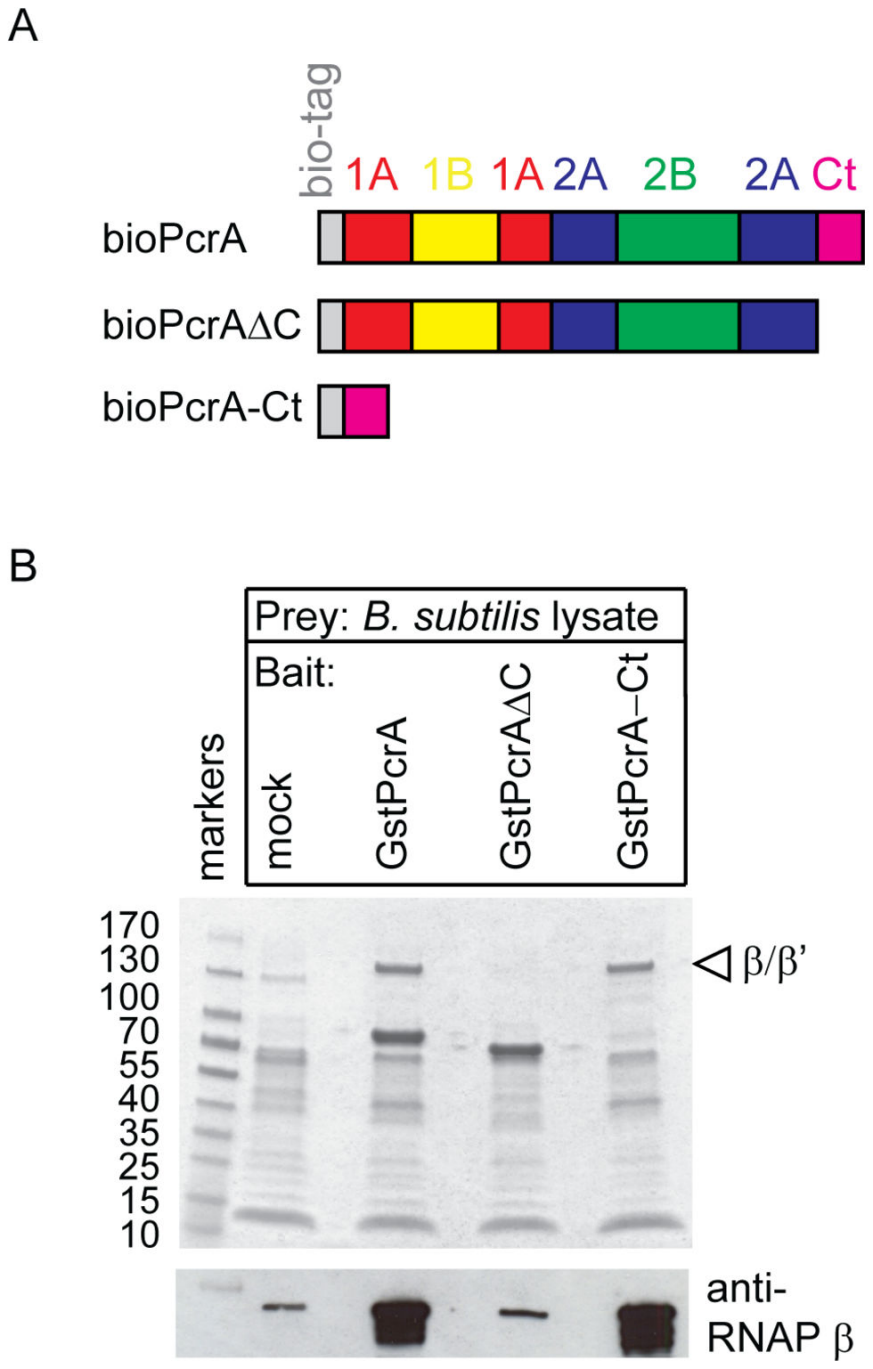
The C-terminal region of PcrA is necessary and sufficient for interaction with RNA polymerase. (A) Primary structure diagrams showing the subdomain organisation in biotinylated PcrA, PcrAΔC and PcrA-Ct proteins. The colouring is the same as in Figure 4 with the biotin tag shown in grey. (B) SDS-PAGE analysis of pull downs from DNA/RNA depleted *B. subtilis* cell extracts using magnetic beads baited with no protein (mock), full length GstPcrA, PcrA with the C-terminus removed (GstPcrAΔC), or the PcrA C-terminus alone (GstPcrA-Ct). The positions of the β and β’ subunits of RNAP are indicated with a triangle. Samples were also analysed by western blot with an anti-RNAP β antibody.

To test this proposal, we constructed modified versions of biotinylated *G. stearothermophilus* PcrA with the C-terminal 72 residues deleted (PcrA-ΔC), as well as a biotinylated protein consisting solely of the C-terminal extension of PcrA (PcrA-Ct). We then investigated the ability of these two protein fragments to pull down RNAP, and compared them in this respect with the full length PcrA helicase (Figure **5**). At equivalent concentrations of bait protein in molar terms, the PcrA-Ct was able to pull down RNAP as efficiently as full-length PcrA. In contrast, the PcrA-ΔC protein was unable to retain RNAP at a level above that detected in the mock pulldown experiment. The inability of PcrA-ΔC to pull down RNAP cannot be explained by an inability of the truncated protein to fold correctly, as the crystal structure of this deletion mutant has been solved previously[34] and the protein retains substantial helicase activity (see below). The C-terminal region of PcrA is therefore required for the interaction with RNAP. 

### RNA polymerase stimulates the apparent helicase activity of PcrA

**Figure 6:**
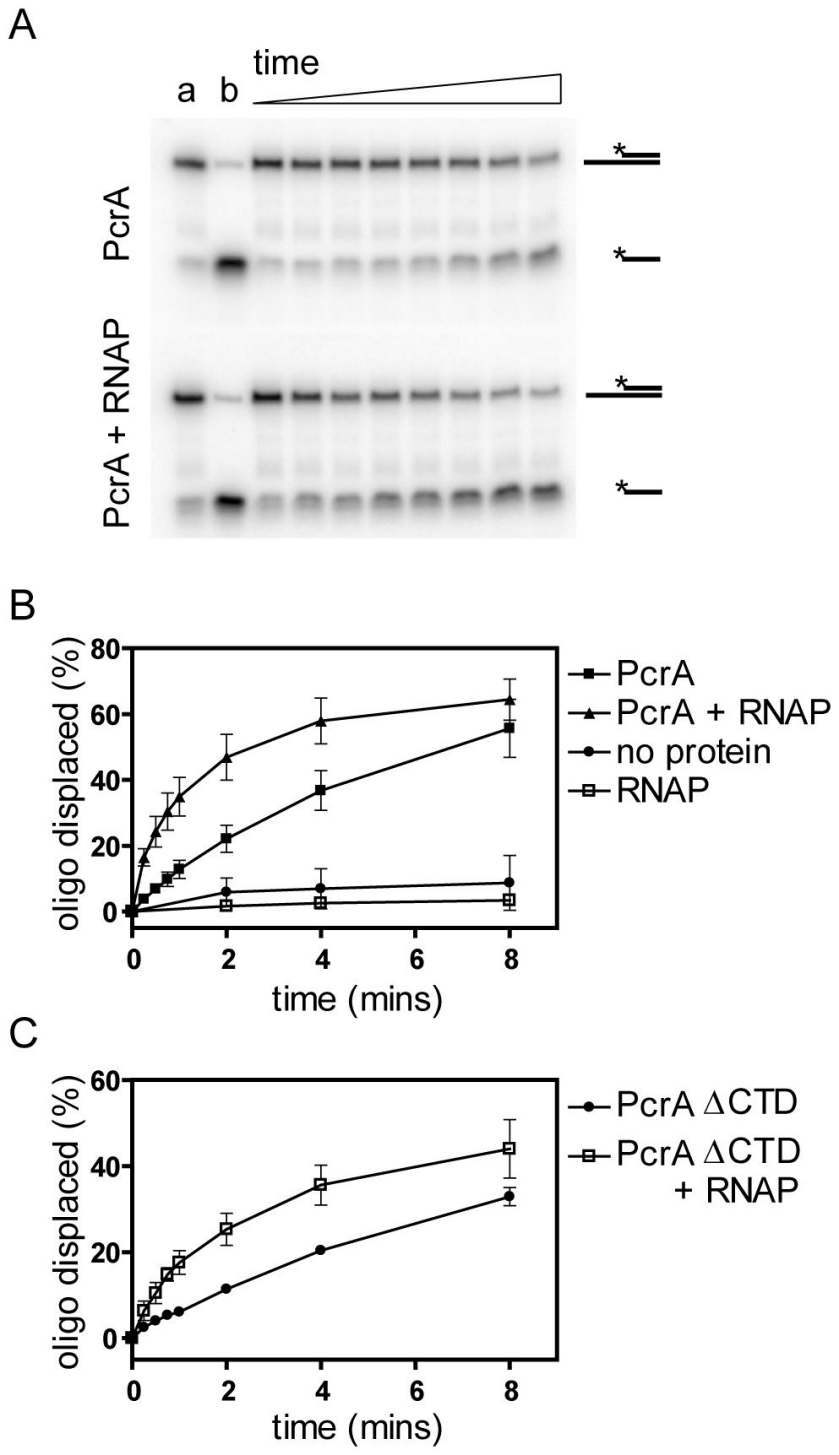
RNA polymerase stimulates the apparent helicase activity of the biotinylated GstPcrA and GstPcrAΔCt proteins. (A) Example helicase assay timecourses showing the unwinding of a partial duplex substrate by PcrA (top panel) or by PcrA in the presence of RNAP (lower panel). Lane a shows the substrate DNA (ie the “no protein” control) and lane b shows heat-denatured substrate. Experiments were performed as described in the methods section. (B) Quantification of helicase timecourse reactions showing the stimulatory effect of RNAP on full length PcrA. (C) Helicase timecourse reactions showing the stimulatory effect of RNAP on PcrA with the C-terminal region deleted. The error bars represent standard deviations about the mean for three independent experiments.

A common hallmark of helicase interaction partners is their ability to stimulate the observed unwinding activity of the enzyme in question[6]. Consequently, we investigated the effect of RNAP on the ability of GstPcrA to unwind a 3′-tailed duplex substrate. In light of the need to employ relatively high protein concentrations to favour interaction with RNAP, sub-optimal conditions for PcrA unwinding activity (ie high ionic strength) were employed in order to be able to detect any possible stimulation of helicase activity over a convenient timecourse of several minutes (see the Methods for details). PcrA helicase alone is able to unwind this substrate as would be expected based on its 3′-5′ polarity[35], but the rate of unwinding is significantly stimulated by the presence of RNAP (Figure **6**). In contrast, RNAP alone is unable to catalyse strand separation under identical conditions. The stimulation of PcrA by RNAP was also observed with alternative helicase substrates and was dose-dependent (Figure S4 in File **S1**). We next tested whether physical interaction between PcrA and RNAP was required for the stimulation of DNA unwinding by investigating the effect of RNAP on the helicase activity of GstPcrAΔC. Although the truncated helicase alone was somewhat less efficient at unwinding the partial duplex substrate than full length PcrA, we still observed stimulation of this activity upon addition of RNAP. Moreover, both the UvrD and Rep helicases were also stimulated by RNAP polymerase in a helicase assay, and PcrA was stimulated effectively by a non-cognate RNAP from *E. coli* (Figure S4 in File **S1**). These observations suggest that the stimulation of DNA unwinding activity by RNAP that we observe is, at least in part, a non-specific effect that is not dependent upon the physical helicase-RNAP interaction we have observed using the pull down and SPR approaches.

## Discussion

In this work we have shown that the disordered C-terminal region of the PcrA helicase interacts directly with RNAP, and that an equivalent interaction between the PcrA homologue UvrD and RNAP is conserved in *E. coli*. The functional significance of this interaction is unknown, but UvrD has been shown to be important in reducing conflicts that occur between DNA replication and transcription in rapidly growing *E. coli* cells[12,13]. Therefore, one may speculate that PcrA/UvrD acts to remove or remodel RNA polymerase complexes that would otherwise impede the process of genome duplication. Consistent with such a view, mutants in RNA polymerase that reduce backtracking of paused RNA polymerase elongation complexes suppress the viability defects associated with loss of UvrD[13]. We also found that RNA polymerase stimulated the helicase activity of the PcrA and UvrD helicases. However, this stimulatory effect was also evident with PcrA lacking the C-terminal interaction domain, as well as with the Rep helicase (which does not interact with RNAP), and so the full significance of this functional interaction is currently unclear. We also tested for effects of PcrA helicase on multiple turnover transcription reactions catalysed by *Bacillus subtilis* RNAP, but did not observe substantial stimulation or inhibition (data not shown). 

We have shown previously that the C-terminus of the related Rep helicase interacts with the replicative helicase DnaB, to form a complex that is also important for reducing replication-transcription conflicts[36]. It is precisely in this C-terminal region that Rep helicase differs most significantly from PcrA and UvrD. Remarkably, within sequence alignments of either the UvrD/PcrA or Rep class of enzyme, this C-terminal extension is one of the most conserved regions. Thus the sequence of this C-terminus may be critical for specifying the function of otherwise highly similar proteins at the level of primary and tertiary structure. On this basis, PcrA and UvrD may be regarded as true orthologues and this enzyme is ubiquitous in bacteria (and possibly essential in all bacteria that do not possess Rep). In contrast, Rep is a distinctive paralogous helicase that occurs only in the limited niche of γ-proteobacteria, but which has an overlapping function with PcrA/UvrD in the suppression of replication-transcription conflicts.

Despite a wealth of structural information for PcrA/UvrD, the C-terminal region has never been resolved[34,37-39]. Therefore all or part of this region may be natively disordered, consistent with its apparent role as a protein:protein interaction hub. However, secondary structure analyses suggest that the final and more highly conserved 50 amino acids will fold into a beta sheet structure[40] and, consistent with this, the domain prediction algorithm Ginzu identifies similarity with the N-terminal Tudor domain of *E. coli* RapA/HepA [41,42]. That domain is thought to be involved in interactions with RNAP that are similar to a structurally related domain of the transcription coupled repair factor Mfd[43]. *E. coli* Mfd and RapA are both classified as SF2 helicases and examples of DNA motor proteins that engage with RNAP in order to modulate its activity. Mfd is a multi-functional RNAP remodelling factor that has been shown to remove RNAP that is stalled at sites of lesions, restart RNAP that is arrested due to backtracking, and terminate transcription at some regulatory signals [44]. RapA is thought to act as a stable component of the transcription machinery and has been shown to stimulate transcription by promoting polymerase recycling [42]. The transcription terminator Rho, a member of helicase Superfamily 5, provides yet another example of a nucleic acid motor protein that has been suggested to engage with the transcription machinery [45].

The interaction between purified PcrA and core RNAP appears to be weak, and may therefore be transient in nature. The surface plasmon resonance data suggests a dissociation constant in the mid to high nanomolar range, and the complex is insufficiently tight to be detected by gel filtration (data not shown). Moreover, the precise site and stoichiometry of the interaction with RNAP is currently unknown. Two hybrid screens suggest that PcrA interacts with the β subunit of RNAP [27]. This is similar to the case for other Tudor domain containing proteins, some of which are known to form a complex of 1:1 stoichiometry with the polymerase [46,47]. Given that the PcrA helicase is monomeric in solution, and that the RNAP complex contains a single β subunit, it seems plausible that the PcrA:RNAP interaction will also adopt a 1:1 stoichiometry. However, PcrA/UvrD has been shown to have unwinding activity *in vitro* as a dimer or higher order oligomer [48,49], and core RNAP certainly contains multiple sites for interactions with regulatory proteins. Therefore, further characterisation of the PcrA:RNAP complex will be required to fully understand its architecture. Pull down assays with RNAP as bait have previously identified PcrA, and also a second SF1 helicase YvgS (sometimes called HelD), as RNAP partners in *Bacillus subtilis* [26]. Interestingly, our mass spectrometry analysis shows that the YvgS helicase is highly enriched in pull downs with PcrA as bait. This suggests either a direct or RNAP-mediated interaction of YvgS with the PcrA helicase, and raises the possibility that RNAP can indeed interact with many such regulatory factors simultaneously.

In addition to the examples discussed above of DNA and RNA motor proteins that bind RNAP and modulate its activity in bacterial systems, there are several interesting cases of eukaryotic helicases that interact with RNA polymerase II. For example, there is emerging evidence that SF2 RecQ-family helicases interact with RNA polymerase to suppress damaging effects of transcription-induced recombination [50,51]. Similarly, recent work on the yeast and human SF1 helicases Sen1p/Senataxin, which have both been shown to interact with RNA polymerase, suggests a role in resolving R-loops that would otherwise present barriers to the replication fork and lead to the formation of double-stranded DNA breaks and associated genomic instability [52-57].

In *E. coli*, the UvrD and Rep helicases can both resolve conflicts between the replisome and the transcription apparatus, but they seem to represent two different solutions to the same problem. Whereas the Rep helicase is targeted to the replisome to help drive it through roadblocks, the work here suggests that UvrD is instead targeted to the transcription apparatus. In this scenario, UvrD could help to displace or remodel stalled RNAP and/or the arrested or aborted transcripts that might otherwise impede the passage of the replication fork. Further clarification of the role of PcrA/UvrD interaction with RNAP in the bacterial cell will await *in vivo* analysis of the effects of appropriate mutant proteins in *E. coli* and *B. subtilis*.

## Supporting Information

File S1 **File contains the Supplementary Materials and Methods** ; Figures S1, S2, S3, S4**; and** Table S1.
(PDF)
