# Supplementary material for: The Conserved C-Terminus of the PcrA/UvrD Helicase Interacts Directly with RNA Polymerase
Source: PLoS One. 2013 Oct 16;8(10):e78141. doi: 10.1371/journal.pone.0078141 (PMC3797733; doi:10.1371/journal.pone.0078141)
Supplement: File S1 — File contains the Supplementary Materials and Methods ; Figures S1, S2, S3, S4; and Table S1. (PDF) [file pone.0078141.s001.pdf]

The conserved C-terminus of the PcrA/UvrD helicase interacts directly with RNA polymerase

Gwynn, E.J., Smith, A.J., Guy, C.P., Savery, N.J., McGlynn, P. and Dillingham, M.S.

File S1 - Supplementary Information

Supplementary Materials and Methods

Helicase assays

To make the circular substrate, a labeled 60 base oligonucleotide (5'AAC GCG CGG GGA GAG GCG GTT TGC GTA TTG GGC GCC AGG GTG GTT TTT CTT TTC ACC AGC) was annealed to M13mp18 DNA (Bayou biolabs) to form a single stranded circular DNA with a 60 base duplex region. Any excess label was removed by passage through an S-400 Microspin column (GE Healthcare). Helicase assays were performed as described in the main text. Briefly, assays on circular substrates were carried out at 20˚C, in a buffer of 25 mM Tris Acetate pH 7.5, 2 mM magnesium Acetate, 0.5 mM DTT and 50 mM NaCl. Proteins at the concentrations indicated were added and incubated together on ice for 5 minutes before addition of 1 nM DNA and a further incubation at 20˚C for 5 minutes. Reactions were then started by the addition of 0.5 mM ATP. Reaction were stopped at the times indicated by mixing with an equal volume of stop buffer (200 mM EDTA, 1% SDS, 10% Ficoll 400, 0.125% bromophenol blue, 0.125% xylene cyanol and 100 nM unlabelled oligo). Analysis of the gels was performed as in the main text methods.

Mass spectroscopy methods and analysis

Each gel lane (experimental and control) was cut into 5 slices and each slice subjected to in-gel tryptic digestion using a ProGest automated digestion unit (Digilab UK). The resulting peptides were fractionated using a Dionex Ultimate 3000 nanoHPLC system in line with an LTQ-Orbitrap Velos mass spectrometer (Thermo Scientific). In brief, peptides in 1% (vol/vol) formic acid were injected onto an Acclaim PepMap C18 nano-trap column (Dionex). After washing with 0.5% (vol/vol) acetonitrile 0.1% (vol/vol) formic acid peptides were resolved on a 250 mm × 75 μm Acclaim PepMap C18 reverse phase analytical column (Dionex) over a 150 min organic gradient, using 7 gradient segments (1-6% solvent B over 1min., 6-15% B over 58min., 15-32%B over 58min., 32-40%B over 3min., 40-90%B over 1min., held at 90%B for 6min and then reduced to 1%B over 1min.) with a flow rate of 300 nl min^{-1}. Solvent A was 0.1% formic acid and Solvent B was aqueous 80% acetonitrile in 0.1% formic acid. Peptides were ionized by nano-electrospray ionization at 2.1 kV using a stainless steel emitter with an internal diameter of 30 μm (Thermo Scientific) and a capillary temperature of 250°C. Tandem mass spectra were acquired using an LTQ- Orbitrap Velos mass spectrometer controlled by Xcalibur 2.1 software (Thermo Scientific) and operated in data-dependent acquisition mode. The Orbitrap was set to analyze the survey scans at 60,000 resolution (at m/z 400) in the mass range m/z 300 to 2000 and the top twenty multiply charged ions in each duty cycle selected for MS/MS in the LTQ linear ion trap. Charge state filtering, where unassigned precursor ions were not selected for fragmentation, and dynamic exclusion (repeat count, 1; repeat duration, 30s; exclusion list size, 500) were used. Fragmentation conditions in the LTQ were as follows: normalized collision energy, 40%; activation q, 0.25; activation time 10ms; and minimum ion selection intensity, 500 counts.

The raw data files were processed and quantified using Proteome Discoverer software v1.2 (Thermo Scientific) and searched against the UniProt B.subtilis database using the SEQUEST (Ver. 28 Rev. 13) algorithm. Peptide precursor mass tolerance was set at 10ppm, and MS/MS tolerance was set at 0.8Da. Search criteria included carbamidomethylation of cysteine (+57.0214) as a fixed modification and oxidation of methionine (+15.9949) as a variable modification. Searches were performed with full tryptic digestion and a maximum of 1 missed cleavage was allowed. The reverse database search option was enabled and all peptide data was filtered to satisfy false discovery rate (FDR) of 5%. The Proteome Discoverer software generates a reverse “decoy” database from the same protein database and any peptides passing the initial filtering parameters that were derived from this decoy database are defined as false positive identifications. The minimum cross-correlation factor (Xcorr) filter was readjusted for each individual charge state separately to optimally meet the predetermined target FDR of 5% based on the number of random false positive matches from the reverse decoy database. Thus each data set has its own passing parameters.

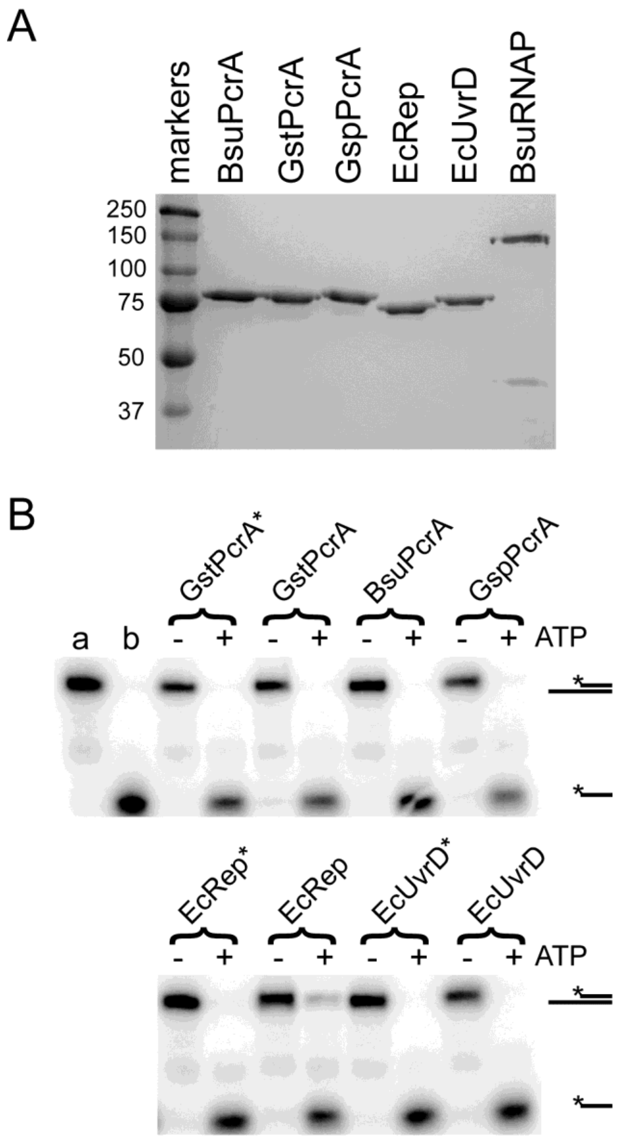

Figure S1. Purity and helicase activity of tagged proteins used in this study. (A) Purified tagged proteins used in this study. Each of the helicases was purified using an N-terminal biotin tag, and the RNAP is his-tagged on the β′ subunit. (B) Helicase assays were performed for 2 minutes with the protein indicated (50 nM) using a 3'-tailed partial duplex DNA substrate (1 nM) as described in the main methods section. Asterisks indicate native (non-biotinylated) proteins.

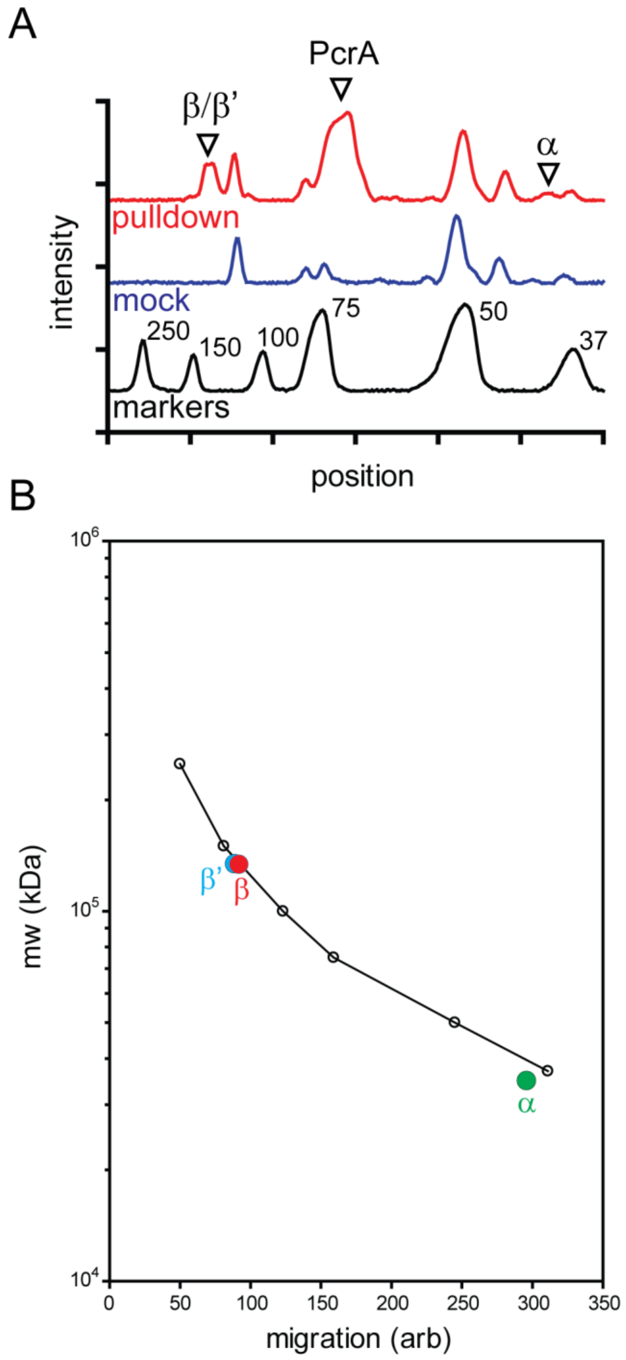

Figure S2. Analysis of the pulldown experiment shown in the main text Figure 1A. (A) Graph shows lane intensity versus migration for the markers (MW indicated), the mock pulldown control experiment and the pulldown. The positions of the PcrA bait and the positions of the three additional novel bands thought to be the β, β' and α subunits of RNAP are shown. (B) The migration position of the peaks for the markers (black) and putative RNAP subunits (coloured) are plotted against their molecular weights. The positions of the three unknown bands are consistent with the molecular weights of the RNAP subunits.

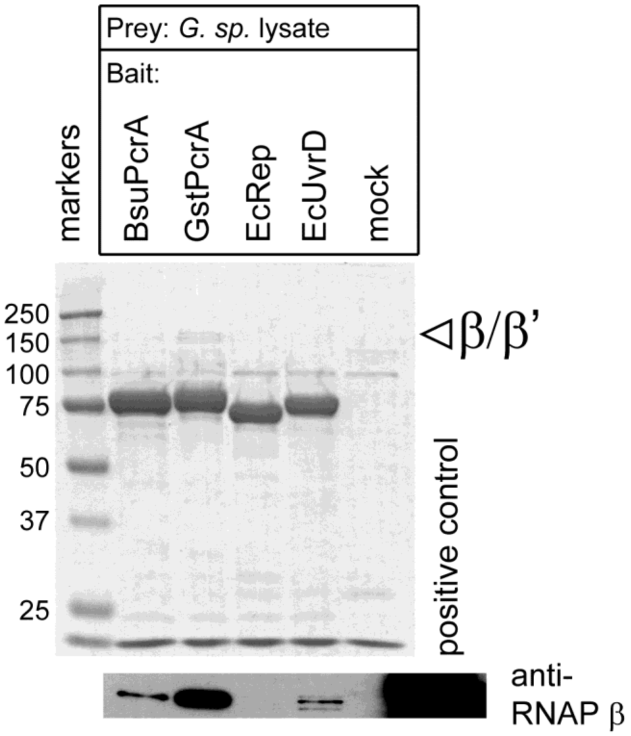

Figure S3. Pulldown experiments were performed as described in the main methods section using biotinylated helicases as bait (indicated) and a *Geobacillus sp.* cell extract. Proteins retained on streptavidin magnetic beads were compared against a mock control experiment in which the beads were not baited using SDS-PAGE. The samples were analysed by western blot for the presence of the β subunit of RNAP using an antibody against *E. coli* RpoB that cross reacts with *Bacillus* RNAP (lower panel). The positive control lane contains purified RNA polymerase.

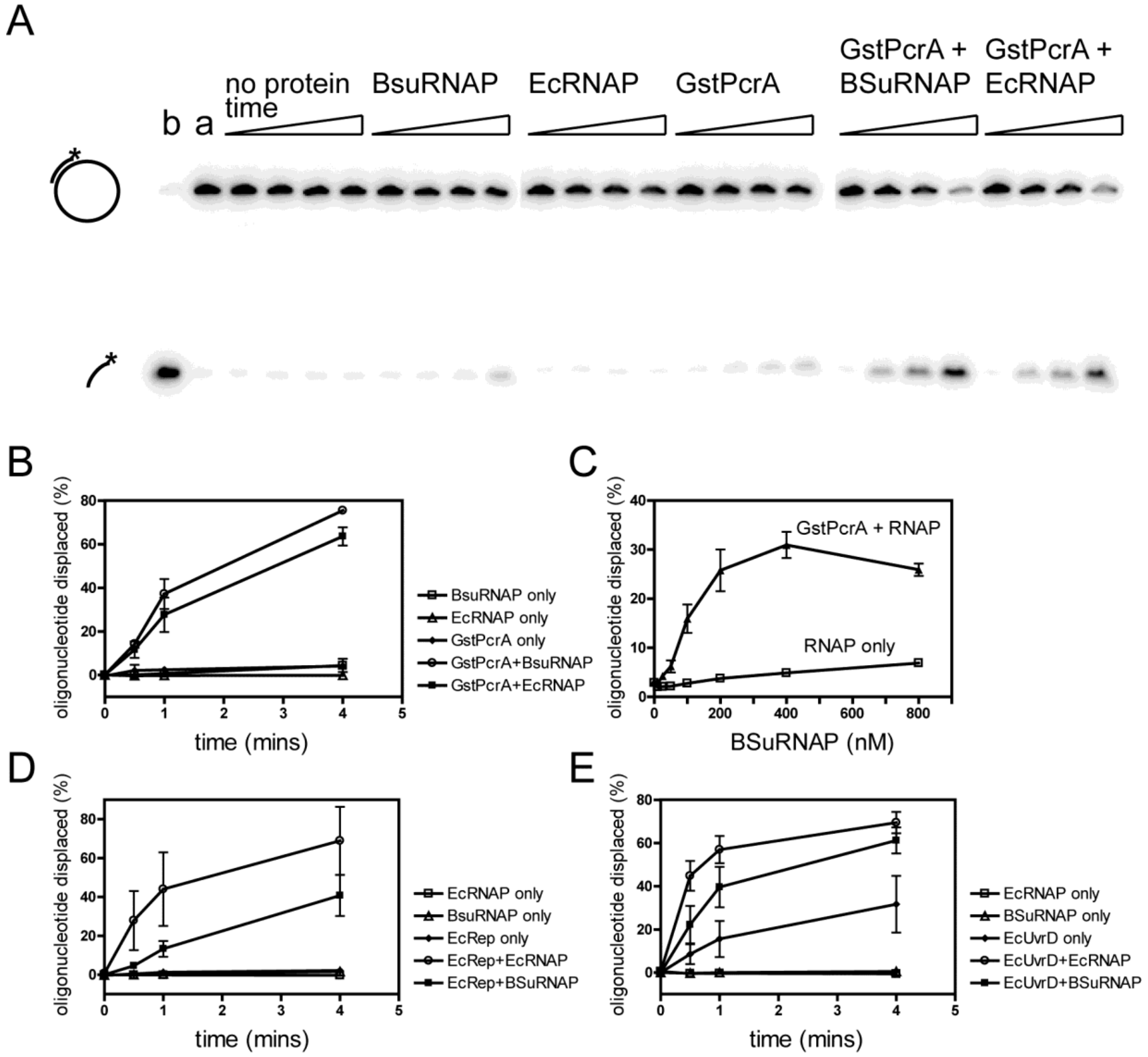

Figure S4. Stimulation of the PcrA, UvrD and Rep helicases by RNA polymerase from *B. subtilis* (Bsu) and *E. coli* (Ec). Experiments were performed as described in the Supplementary Methods section. (A) Representative raw data from helicase assay timecourse experiments using a circular substrate with a 60 bp partial duplex region. PcrA (50 nM) is a very poorly processive helicase and barely unwinds this substrate. Addition of either *B. subtilis* or *E. coli* RNAP (both 100 nM) stimulates the observed unwinding activity, but the RNAP complexes display no helicase activity by themselves. (B) Quantitation of the data shown in panel A. The error bars represent the standard deviation for three independent experiments. (C) Dose-dependent stimulation of PcrA (25 nM) unwinding activity by *B. subtilis* RNAP, measured at a 60 second timepoint. (D) Stimulation of *E. coli* Rep helicase by RNAP. (E) Stimulation of *E. coli* UvrD helicase by RNAP. The experiments shown in panels D and E are exactly equivalent to those in panel B but the identity of the helicase being studied is different as indicated.

Table S1

Mass spectroscopy analysis of proteins enriched from *Bacillus subtilis* cell extracts by pull down with BsuPcrA. A confidence score is given for the identification of each protein listed in the table below in both the experimental and mock control pull down lanes of the gel. The proteins are listed in order of the confidence score for the experimental lane. The confidence score for the experimental lane divided by that for the control is taken as a measure of the relative abundance of the protein in the pull down compared to the control. The main section of the paper reports the eight most confident protein identification hits in the experiment which also satisfy the requirement of being at least five-fold enriched in the experiment versus the control. The table below shows the entire dataset of proteins identified in either or both the experiment and the control (see also **Supplementary Methods** section). Raw data sets are available from the author upon request.

Accession	Description	Score Exp/Control	Score Control	Coverage control	PSMs control	Peptides control	Score exp	Coverage exp	PSMs exp	Peptides exp
**O34580**	ATP-dependent DNA helicase pcrA OS=Bacillus subtilis GN=pcrA PE=1 SV=1 - [PCRA_BACSU]	31.45	1955.49	29.91	88	19	61504.67	73.07	2568	65
**Q9KWU4**	Pyruvate carboxylase OS=Bacillus subtilis GN=pyc PE=2 SV=1 - [PYC_BACSU]	1.05	13998.19	57.75	804	68	14640.30	63.33	659	78
**P37871**	DNA-directed RNA polymerase subunit beta' OS=Bacillus subtilis GN=rpoC PE=3 SV=4 - [RPOC_BACSU]	5.10	2308.17	31.78	144	35	11760.95	63.97	573	73
**P37870**	DNA-directed RNA polymerase subunit beta OS=Bacillus subtilis GN=rpoB PE=1 SV=2 - [RPOB_BACSU]	7.23	1362.94	34.95	99	37	9847.50	65.13	515	78
**P96583**	DNA topoisomerase 3 OS=Bacillus subtilis GN=topB PE=3 SV=1 - [TOP3_BACSU]	1.28	6619.62	58.05	283	47	8456.72	60.94	346	51
**P54423**	Cell wall-associated protease OS=Bacillus subtilis GN=wprA PE=1 SV=2 - [WPRA_BACSU]	1.00	8115.63	44.52	428	42	8143.21	42.39	375	37
**Q45066**	DNA topoisomerase 4 subunit A OS=Bacillus subtilis GN=parC PE=3 SV=2 - [PARC_BACSU]	1.89	3026.61	40.07	173	33	5713.58	48.76	271	39
**Q04747**	Surfactin synthetase subunit 2 OS=Bacillus subtilis GN=srfAB PE=1 SV=3 - [SRFAB_BACSU]	0.78	6993.08	27.10	391	84	5451.99	29.56	326	90
**O07603**	Putative aminopeptidase yhfE OS=Bacillus subtilis GN=yhfE PE=3 SV=1 - [YHFE_BACSU]	1.10	4541.19	59.54	259	19	4996.95	56.36	288	21
**P27206**	Surfactin synthetase subunit 1 OS=Bacillus subtilis GN=srfAA PE=1 SV=4 - [SRFAA_BACSU]	0.80	5983.66	23.95	324	74	4811.48	29.33	252	85
**P39814**	DNA topoisomerase 1 OS=Bacillus subtilis GN=topA PE=3 SV=1 - [TOP1_BACSU]	1.89	2510.17	38.78	139	25	4741.62	56.73	227	36
**Q07833**	Wall-associated protein OS=Bacillus subtilis GN=wapA PE=1 SV=2 - [WAPA_BACSU]	1.35	2845.53	20.99	170	39	3833.61	22.37	170	44
**P96614**	DEAD-box ATP-dependent RNA helicase cshA OS=Bacillus subtilis GN=cshA PE=1 SV=2 - [CSHA_BACSU]	1.43	2662.00	41.50	152	21	3817.41	41.09	182	23
**O34425**	Glyceraldehyde-3-phosphate dehydrogenase 2 OS=Bacillus subtilis GN=gapB PE=1 SV=1 - [G3P2_BACSU]	1.16	3129.12	38.53	112	12	3637.95	42.65	126	15
**P17889**	Translation initiation factor IF-2 OS=Bacillus subtilis GN=infB PE=3 SV=1 - [IF2_BACSU]	1.68	1789.07	27.09	94	21	3012.20	36.03	125	26
**Q01465**	Rod shape-determining protein mreB OS=Bacillus subtilis GN=mreB PE=3 SV=3 - [MREB_BACSU]	1.32	2243.51	61.72	99	20	2972.03	64.69	123	21
**P39215**	Methyl-accepting chemotaxis protein mcpB OS=Bacillus subtilis GN=mcpB PE=1 SV=2 - [MCPB_BACSU]	1.01	2850.58	38.67	106	22	2878.57	51.51	88	24
**P16263**	Dihydrolipoyllysine-residue succinyltransferase component of 2-oxoglutarate dehydrogenase complex OS=Bacillus subtilis GN=odhB PE=3 SV=2 - [ODO2_BACSU]	0.71	3961.00	50.36	221	20	2827.27	46.52	156	20

**P80886**	Succinyl-CoA ligase [ADP-forming] subunit beta OS=Bacillus subtilis GN=sucC PE=1 SV=2 - [SUCC_BACSU]	1.02	2707.95	57.66	109	22	2755.96	58.70	101	23
**P46320**	Probable 6-phospho-beta-glucosidase OS=Bacillus subtilis GN=licH PE=2 SV=1 - [LICH_BACSU]	0.77	3514.43	49.32	164	20	2704.63	44.12	122	20
**Q03222**	Transcription termination factor rho OS=Bacillus subtilis GN=rho PE=3 SV=3 - [RHO_BACSU]	1.13	2315.65	42.86	140	18	2617.11	48.24	118	20
**P37571**	Negative regulator of genetic competence clpC/mecB OS=Bacillus subtilis GN=clpC PE=1 SV=1 - [CLPC_BACSU]	1.22	1955.30	35.43	105	28	2377.51	37.28	98	27
**P37809**	ATP synthase subunit beta OS=Bacillus subtilis GN=atpD PE=1 SV=1 - [ATPB_BACSU]	0.82	2754.24	43.76	125	16	2268.10	49.05	106	16
**O34784**	Uncharacterized membrane protein yobI OS=Bacillus subtilis GN=yobI PE=4 SV=1 - [YOBI_BACSU]	1.22	1800.02	29.73	115	39	2197.27	36.14	97	44
**P39793**	Penicillin-binding protein 1A/1B OS=Bacillus subtilis GN=ponA PE=1 SV=1 - [PBPA_BACSU]	1.18	1777.56	29.32	94	25	2097.73	36.21	91	27
**P20429**	DNA-directed RNA polymerase subunit alpha OS=Bacillus subtilis GN=rpoA PE=1 SV=1 - [RPOA_BACSU]	6.17	339.38	25.16	17	7	2092.65	40.45	112	16
**O31777**	Putative 8-amino-7-oxononanoate synthase/2-amino-3-ketobutyrate coenzyme A ligase OS=Bacillus subtilis GN=kbl PE=3 SV=1 - [BIKB_BACSU]	1.40	1417.76	50.26	92	18	1978.27	51.02	115	17
**P08821**	DNA-binding protein HU 1 OS=Bacillus subtilis GN=hupA PE=1 SV=2 - [DBH1_BACSU]	0.88	2220.37	59.78	102	7	1961.88	46.74	80	7
**P21880**	Dihydrolipoyl dehydrogenase OS=Bacillus subtilis GN=pdhD PE=3 SV=1 - [DLDH1_BACSU]	0.61	3216.19	41.91	131	16	1955.64	36.60	62	14
**O32178**	Probable 3-hydroxyacyl-CoA dehydrogenase OS=Bacillus subtilis GN=fadN PE=2 SV=2 - [FADN_BACSU]	1.91	1016.77	30.93	75	22	1943.08	47.40	92	31
**P18157**	Glycerol kinase OS=Bacillus subtilis GN=glpK PE=1 SV=2 - [GLPK_BACSU]	0.78	2431.37	34.48	98	19	1905.70	32.06	64	16
**P50735**	NAD-specific glutamate dehydrogenase OS=Bacillus subtilis GN=gudB PE=1 SV=2 - [GUDB_BACSU]	1.02	1833.11	34.19	80	13	1870.41	37.70	80	14
**O32215**	Helicase IV OS=Bacillus subtilis GN=helD PE=1 SV=1 - [HELD_BACSU]	#DIV/0!					1862.87	39.92	71	27
**O34750**	Putative ATP-dependent RNA helicase yfmL OS=Bacillus subtilis GN=yfmL PE=3 SV=1 - [YFML_BACSU]	1.15	1574.90	41.76	91	16	1808.25	47.34	91	21
**P37942**	Lipoamide acyltransferase component of branched-chain alpha-keto acid dehydrogenase complex OS=Bacillus subtilis GN=bfmBB PE=3 SV=1 - [ODB2_BACSU]	0.80	2192.03	39.86	93	15	1755.62	36.79	66	14
**P37945**	ATP-dependent protease La 1 OS=Bacillus subtilis GN=lonA PE=1 SV=1 - [LON1_BACSU]	1.59	1098.42	26.23	73	20	1743.34	35.27	82	26
**P33166**	Elongation factor Tu OS=Bacillus subtilis GN=tuf PE=3 SV=1 - [EFTU_BACSU]	0.56	3079.86	57.58	145	23	1737.05	49.75	79	18
**P14951**	UvrABC system protein C OS=Bacillus subtilis GN=uvrC PE=3 SV=2 - [UVRC_BACSU]	1.92	904.41	21.02	42	12	1733.66	42.71	99	29
**Q59192**	DNA topoisomerase 4 subunit B OS=Bacillus subtilis GN=parE PE=3 SV=2 - [PARE_BACSU]	1.36	1263.19	21.07	53	15	1723.10	33.13	75	20
**P35159**	Ribosomal large subunit pseudouridine synthase B OS=Bacillus subtilis GN=rluB PE=1 SV=2 - [RLUB_BACSU]	1.36	1252.18	60.25	65	17	1697.99	52.05	73	17
**O32047**	Protein-export membrane protein secDF OS=Bacillus subtilis GN=secDF PE=1 SV=2 - [SECDF_BACSU]	1.09	1441.85	28.49	81	18	1567.77	27.14	67	17
**P21464**	30S ribosomal protein S2 OS=Bacillus subtilis GN=rpsB PE=1 SV=3 - [RS2_BACSU]	2.26	685.00	41.87	31	12	1549.37	56.10	66	16
**O31545**	Uncharacterized RNA methyltransferase yfjO OS=Bacillus subtilis GN=yfjO PE=3 SV=1 - [YFJO_BACSU]	1.26	1230.22	41.20	71	20	1546.50	55.15	91	27
**P38021**	Ornithine aminotransferase OS=Bacillus subtilis GN=rocD PE=2 SV=2 - [OAT_BACSU]	0.92	1670.87	44.89	89	14	1545.21	45.89	84	15
**O34833**	Uncharacterized protein yceH OS=Bacillus subtilis GN=yceH PE=3 SV=1 - [YCEH_BACSU]	1.00	1473.58	50.69	56	16	1480.25	49.04	57	17
**P21465**	30S ribosomal protein S3 OS=Bacillus subtilis GN=rpsC PE=1 SV=4 - [RS3_BACSU]	1.25	1176.99	53.67	42	10	1465.54	60.55	58	14

**P80868**	Elongation factor G OS=Bacillus subtilis GN=fusA PE=1 SV=3 - [EFG_BACSU]	1.44	999.34	33.96	68	19	1443.20	37.57	65	21
**P55872**	Translation initiation factor IF-3 OS=Bacillus subtilis GN=infC PE=3 SV=3 - [IF3_BACSU]	1.39	1036.49	53.18	64	8	1440.43	53.18	77	9
**P54475**	DEAD-box ATP-dependent RNA helicase cshB OS=Bacillus subtilis GN=cshB PE=1 SV=1 - [CSHB_BACSU]	1.22	1169.90	36.76	46	15	1427.48	47.95	70	20
**P06574**	RNA polymerase sigma-B factor OS=Bacillus subtilis GN=sigB PE=1 SV=3 - [RPSB_BACSU]	#DIV/0!					1422.19	37.79	57	11
**P36949**	D-ribose-binding protein OS=Bacillus subtilis GN=rbsB PE=3 SV=2 - [RBSB_BACSU]	0.94	1510.37	40.33	57	14	1412.53	40.33	49	14
**P71073**	Uncharacterized protein yukF OS=Bacillus subtilis GN=yukF PE=3 SV=2 - [YUKF_BACSU]	1.14	1214.78	43.13	69	16	1385.57	43.36	74	17
**P94593**	Uncharacterized ATP-dependent helicase ywqA OS=Bacillus subtilis GN=ywqA PE=3 SV=2 - [YWQA_BACSU]	18.98	71.70	4.88	5	4	1360.88	32.21	73	28
**P39587**	Putative ribosomal RNA large subunit methyltransferase ywbD OS=Bacillus subtilis GN=ywbD PE=3 SV=1 - [YWBD_BACSU]	1.77	764.54	32.07	40	14	1354.72	33.33	48	15
**P71011**	Antilisterial bacteriocin subtilosin biosynthesis protein albA OS=Bacillus subtilis GN=albA PE=2 SV=1 - [ALBA_BACSU]	0.84	1531.19	41.74	91	18	1287.70	32.59	67	15
**O34863**	UvrABC system protein A OS=Bacillus subtilis GN=uvrA PE=3 SV=1 - [UVRA_BACSU]	1.70	758.13	13.06	40	12	1287.26	31.03	46	25
**P39214**	Methyl-accepting chemotaxis protein mcpA OS=Bacillus subtilis GN=mcpA PE=1 SV=2 - [MCPA_BACSU]	1.38	929.95	22.09	49	13	1282.49	27.84	49	20
**P02968**	Flagellin OS=Bacillus subtilis GN=hag PE=1 SV=2 - [FLA_BACSU]	0.82	1541.39	41.12	71	10	1269.69	36.84	41	8
**P37941**	2-oxoisovalerate dehydrogenase subunit beta OS=Bacillus subtilis GN=bfmBAB PE=1 SV=1 - [ODBB_BACSU]	1.06	1193.36	44.34	61	14	1262.95	45.57	63	14
**O34324**	Dihydrolipoyl dehydrogenase OS=Bacillus subtilis GN=acoL PE=3 SV=1 - [DLDH3_BACSU]	0.76	1670.80	29.26	70	13	1262.56	31.44	38	14
**O34529**	6-phosphofructokinase OS=Bacillus subtilis GN=pfkA PE=3 SV=1 - [K6PF_BACSU]	1.20	1047.98	24.45	43	8	1259.95	28.53	47	9
**P23129**	2-oxoglutarate dehydrogenase E1 component OS=Bacillus subtilis GN=odhA PE=3 SV=3 - [ODO1_BACSU]	1.08	1111.76	22.35	61	16	1199.77	30.30	44	21
**P71018**	Phosphate acyltransferase OS=Bacillus subtilis GN=plsX PE=1 SV=2 - [PLSX_BACSU]	0.95	1253.79	37.24	45	11	1197.16	37.24	51	10
**P39751**	MreB-like protein OS=Bacillus subtilis GN=mbl PE=3 SV=1 - [MBL_BACSU]	1.11	1060.71	35.44	51	13	1174.54	37.24	49	13
**Q05852**	UTP--glucose-1-phosphate uridylyltransferase OS=Bacillus subtilis GN=gtaB PE=1 SV=1 - [GTAB_BACSU]	1.06	1099.41	51.03	62	18	1170.18	44.86	54	16
**O06975**	Putative sporulation transcription regulator whiA OS=Bacillus subtilis GN=whiA PE=3 SV=1 - [WHIA_BACSU]	1.18	987.93	29.11	35	9	1165.19	35.76	41	11
**O07906**	Uncharacterized HTH-type transcriptional regulator yraN OS=Bacillus subtilis GN=yraN PE=4 SV=1 - [YRAN_BACSU]	1.51	755.40	37.37	36	11	1142.12	39.10	56	13
**P17865**	Cell division protein ftsZ OS=Bacillus subtilis GN=ftsZ PE=1 SV=3 - [FTSZ_BACSU]	1.04	1080.38	35.08	47	11	1126.27	36.91	47	11
**O34595**	Probable tRNA sulfurtransferase OS=Bacillus subtilis GN=thiI PE=3 SV=2 - [THII_BACSU]	0.85	1297.13	34.41	70	13	1104.71	36.91	62	14
**P39597**	Putative peroxidase ywbN OS=Bacillus subtilis GN=ywbN PE=3 SV=1 - [YWBN_BACSU]	1.21	908.17	30.05	48	13	1097.54	36.78	52	15
**P42175**	Nitrate reductase alpha chain OS=Bacillus subtilis GN=narG PE=3 SV=2 - [NARG_BACSU]	1.63	635.10	16.78	43	16	1035.11	24.10	53	22
**P09124**	Glyceraldehyde-3-phosphate dehydrogenase 1 OS=Bacillus subtilis GN=gapA PE=1 SV=2 - [G3P1_BACSU]	1.33	778.21	35.82	35	8	1031.63	39.70	48	11
**O34591**	Acetoin:2,6-dichlorophenolindophenol oxidoreductase subunit beta OS=Bacillus subtilis GN=acoB PE=3 SV=3 - [ACOB_BACSU]	1.00	1020.19	34.50	40	11	1016.20	41.52	48	14
**P24327**	Foldase protein prsA OS=Bacillus subtilis GN=prsA PE=1 SV=1 - [PRSA_BACSU]	1.17	869.70	55.82	40	17	1013.55	55.82	41	17

**P37940**	2-oxoisovalerate dehydrogenase subunit alpha OS=Bacillus subtilis GN=bfmBAA PE=1 SV=1 - [ODBA_BACSU]	1.07	913.70	36.67	50	13	977.27	43.64	50	14
**O34789**	Uncharacterized protein ydjI OS=Bacillus subtilis GN=ydjI PE=4 SV=1 - [YDJI_BACSU]	0.70	1397.06	42.41	82	13	973.94	41.18	61	12
**P30949**	Glutamate-1-semialdehyde 2,1-aminomutase OS=Bacillus subtilis GN=hemL PE=1 SV=1 - [GSA_BACSU]	0.98	987.99	33.49	45	10	969.16	33.95	40	10
**P71079**	Enoyl-[acyl-carrier-protein] reductase [NADPH] OS=Bacillus subtilis GN=fabL PE=1 SV=1 - [FABL_BACSU]	1.59	607.10	42.80	26	12	966.23	51.20	41	13
**O32129**	Lipoyl synthase OS=Bacillus subtilis GN=lipA PE=3 SV=2 - [LIPA_BACSU]	1.56	617.81	43.62	33	12	963.30	51.34	45	14
**P37877**	Acetate kinase OS=Bacillus subtilis GN=ackA PE=3 SV=1 - [ACKA_BACSU]	1.28	745.65	40.76	33	11	953.21	45.82	37	13
**P17820**	Chaperone protein dnaK OS=Bacillus subtilis GN=dnaK PE=1 SV=3 - [DNAK_BACSU]	0.69	1363.85	32.41	48	17	939.16	36.66	42	16
**P05653**	DNA gyrase subunit A OS=Bacillus subtilis GN=gyrA PE=3 SV=1 - [GYRA_BACSU]	1.99	472.26	18.76	33	14	937.75	35.57	43	23
**O31760**	Ribonuclease J 2 OS=Bacillus subtilis GN=rnjB PE=1 SV=3 - [RNJ2_BACSU]	0.95	983.48	37.48	55	17	934.72	35.50	41	17
**P39126**	Isocitrate dehydrogenase [NADP] OS=Bacillus subtilis GN=icd PE=1 SV=1 - [IDH_BACSU]	0.90	1027.59	37.12	46	17	929.71	34.99	43	15
**P37954**	UvrABC system protein B OS=Bacillus subtilis GN=uvrB PE=1 SV=2 - [UVRB_BACSU]	23.64	39.09	6.35	4	4	924.20	26.32	53	17
**O34666**	Carboxy-terminal processing protease ctpA OS=Bacillus subtilis GN=ctpA PE=2 SV=1 - [CTPA_BACSU]	0.90	999.72	30.69	44	14	901.57	28.97	33	14
**P36947**	Ribose import ATP-binding protein rbsA OS=Bacillus subtilis GN=rbsA PE=3 SV=2 - [RBSA_BACSU]	0.43	2084.77	41.38	82	22	894.97	35.70	44	18
**P16971**	Protein recA OS=Bacillus subtilis GN=recA PE=1 SV=2 - [RECA_BACSU]	0.91	971.96	27.87	42	9	882.78	29.31	39	9
**P21467**	30S ribosomal protein S5 OS=Bacillus subtilis GN=rpsE PE=1 SV=3 - [RS5_BACSU]	0.91	963.41	66.87	61	10	875.07	66.87	43	10
**P34957**	Quinol oxidase subunit 2 OS=Bacillus subtilis GN=qoxA PE=1 SV=4 - [QOX2_BACSU]	1.05	815.11	19.00	32	4	856.39	28.66	47	7
**P17631**	Chaperone protein dnaJ OS=Bacillus subtilis GN=dnaJ PE=2 SV=3 - [DNAJ_BACSU]	2.59	329.32	32.27	19	10	854.24	34.67	41	13
**P37808**	ATP synthase subunit alpha OS=Bacillus subtilis GN=atpA PE=1 SV=3 - [ATPA_BACSU]	0.68	1240.48	36.06	85	17	844.79	28.29	45	13
**P54419**	S-adenosylmethionine synthetase OS=Bacillus subtilis GN=metK PE=3 SV=2 - [METK_BACSU]	0.72	1161.57	30.75	56	12	838.07	28.50	36	11
**O35033**	Probable coenzyme A biosynthesis bifunctional protein coaBC OS=Bacillus subtilis GN=coaBC PE=3 SV=1 - [COABC_BACSU]	1.18	696.51	30.79	32	11	822.80	38.18	42	14
**O31404**	Acetoin:2,6-dichlorophenolindophenol oxidoreductase subunit alpha OS=Bacillus subtilis GN=acoA PE=2 SV=2 - [ACOA_BACSU]	0.91	894.04	35.14	48	10	815.51	34.53	44	9
**P28264**	Cell division protein ftsA OS=Bacillus subtilis GN=ftsA PE=1 SV=2 - [FTSA_BACSU]	1.02	792.57	20.23	29	9	806.01	20.00	28	8
**P39578**	Protein dltD OS=Bacillus subtilis GN=dltD PE=1 SV=1 - [DLTD_BACSU]	1.36	591.89	23.47	28	9	805.92	28.83	43	11
**P54576**	Methyl-accepting chemotaxis protein mcpC OS=Bacillus subtilis GN=mcpC PE=1 SV=2 - [MCPC_BACSU]	0.84	942.46	33.89	44	24	793.30	34.50	47	21
**O34660**	Putative aldehyde dehydrogenase dhaS OS=Bacillus subtilis GN=dhaS PE=3 SV=1 - [ALDH4_BACSU]	0.91	865.27	27.47	45	14	788.62	30.30	41	14
**O31455**	Putative hydrolase ybfO OS=Bacillus subtilis GN=ybfO PE=4 SV=1 - [YBFO_BACSU]	0.65	1203.64	38.12	65	14	776.98	36.77	61	14
**Q45493**	Ribonuclease J 1 OS=Bacillus subtilis GN=rnjA PE=1 SV=1 - [RNJ1_BACSU]	0.96	791.98	35.86	42	15	761.03	42.88	39	19
**P42919**	50S ribosomal protein L2 OS=Bacillus subtilis GN=rplB PE=1 SV=3 - [RL2_BACSU]	0.84	901.73	38.27	35	8	760.03	31.77	28	7

**P19405**	Alkaline phosphatase 3 OS=Bacillus subtilis GN=phoB PE=1 SV=4 - [PPB3_BACSU]	0.68	1079.08	28.57	58	12	733.93	23.38	37	9
**P39633**	NAD-specific glutamate dehydrogenase OS=Bacillus subtilis GN=rocG PE=1 SV=3 - [DHE2_BACSU]	1.11	656.29	34.43	44	12	731.50	33.96	45	12
**P13242**	CTP synthase OS=Bacillus subtilis GN=pyrG PE=3 SV=1 - [PYRG_BACSU]	0.65	1110.99	29.53	48	15	727.62	36.64	37	18
**P50863**	Protein mrp homolog salA OS=Bacillus subtilis GN=salA PE=2 SV=1 - [SALA_BACSU]	1.59	454.63	30.97	32	9	721.46	42.90	45	12
**O32177**	3-ketoacyl-CoA thiolase OS=Bacillus subtilis GN=fadA PE=2 SV=1 - [FADA_BACSU]	1.46	493.88	28.90	27	10	720.57	36.57	41	12
**P39778**	ATP-dependent hsl protease ATP-binding subunit hslU OS=Bacillus subtilis GN=hslU PE=3 SV=1 - [HSLU_BACSU]	0.64	1124.63	29.98	52	11	718.44	33.83	26	13
**P19670**	UDP-N-acetylglucosamine 1-carboxyvinyltransferase 2 OS=Bacillus subtilis GN=murAB PE=1 SV=4 - [MURA2_BACSU]	1.29	556.98	18.65	22	8	715.83	31.24	31	12
**P51833**	Ribonuclease 3 OS=Bacillus subtilis GN=rnc PE=3 SV=2 - [RNC_BACSU]	0.87	824.81	36.55	32	9	715.35	39.36	33	11
**O34788**	(R,R)-butanediol dehydrogenase OS=Bacillus subtilis GN=bdhA PE=3 SV=1 - [BDHA_BACSU]	0.75	927.93	25.43	40	9	700.53	26.88	32	9
**O05389**	Uncharacterized oxidoreductase yrbE OS=Bacillus subtilis GN=yrbE PE=3 SV=2 - [YRBE_BACSU]	0.79	883.94	53.67	36	13	694.39	49.56	35	12
**P14193**	Ribose-phosphate pyrophosphokinase OS=Bacillus subtilis GN=prs PE=1 SV=1 - [KPRS_BACSU]	1.15	604.20	34.07	26	8	692.27	34.07	26	8
**P28598**	60 kDa chaperonin OS=Bacillus subtilis GN=groL PE=1 SV=3 - [CH60_BACSU]	0.39	1785.28	30.70	80	19	688.32	27.02	27	14
**P96629**	ICEBs1 integrase OS=Bacillus subtilis GN=int PE=3 SV=1 - [INT_BACSU]	1.06	647.20	23.64	33	8	685.91	33.15	39	12
**P13799**	Sensor protein degS OS=Bacillus subtilis GN=degS PE=1 SV=2 - [DEGS_BACSU]	0.79	848.15	41.30	39	17	672.79	48.05	38	19
**O06491**	Glutamyl-tRNA(Gln) amidotransferase subunit A OS=Bacillus subtilis GN=gatA PE=3 SV=3 - [GATA_BACSU]	1.44	467.21	28.45	23	11	671.56	30.72	27	11
**P24219**	RNA polymerase sigma-54 factor OS=Bacillus subtilis GN=sigL PE=3 SV=1 - [RP54_BACSU]	3.93	166.18	0.00	3	1	653.51	33.26	27	12
**P54472**	UPF0135 protein yqfO OS=Bacillus subtilis GN=yqfO PE=3 SV=2 - [YQFO_BACSU]	1.45	443.09	31.10	35	10	640.57	32.44	50	11
**P37810**	ATP synthase gamma chain OS=Bacillus subtilis GN=atpG PE=3 SV=2 - [ATPG_BACSU]	0.77	820.89	24.74	30	10	634.25	28.92	26	11
**P39209**	Methyl-accepting chemotaxis protein tlpC OS=Bacillus subtilis GN=tlpC PE=3 SV=3 - [TLPC_BACSU]	1.06	598.43	16.23	28	9	632.26	29.32	23	14
**O31550**	Dihydrolipoyllysine-residue acetyltransferase component of acetoin cleaving system OS=Bacillus subtilis GN=acoC PE=3 SV=1 - [ACOC_BACSU]	1.65	378.24	27.39	32	10	623.55	29.40	28	11
**P40406**	Uncharacterized lipoprotein ybbD OS=Bacillus subtilis GN=ybbD PE=1 SV=1 - [YBBD_BACSU]	0.71	871.95	31.62	40	17	619.45	26.79	22	13
**O32231**	Ribonuclease R OS=Bacillus subtilis GN=rnr PE=3 SV=1 - [RNR_BACSU]	2.46	244.83	13.74	16	9	602.69	26.06	30	17
**O32038**	Aspartyl-tRNA synthetase OS=Bacillus subtilis GN=aspS PE=3 SV=1 - [SYD_BACSU]	1.69	351.74	17.06	22	9	596.17	30.41	25	16
**O31644**	Probable transcriptional regulator manR OS=Bacillus subtilis GN=manR PE=3 SV=2 - [MANR_BACSU]	3.61	164.25	14.66	15	7	593.65	31.94	38	17
**P37476**	Cell division protease ftsH homolog OS=Bacillus subtilis GN=ftsH PE=3 SV=1 - [FTSH_BACSU]	0.58	1012.68	24.18	43	15	590.42	24.65	27	14
**O32165**	FeS cluster assembly protein sufD OS=Bacillus subtilis GN=sufD PE=3 SV=1 - [SUFD_BACSU]	1.06	550.19	25.86	31	9	585.12	27.23	21	10
**P29072**	Chemotaxis protein cheA OS=Bacillus subtilis GN=cheA PE=3 SV=2 - [CHEA_BACSU]	1.26	457.05	14.58	24	9	577.13	23.96	24	13
**P80700**	Elongation factor Ts OS=Bacillus subtilis GN=tsf PE=1 SV=3 - [EFTS_BACSU]	0.54	1067.78	48.12	45	13	572.72	42.32	27	11

**P94391**	1-pyrroline-5-carboxylate dehydrogenase 2 OS=Bacillus subtilis GN=ycgN PE=3 SV=2 - [ROCA2_BACSU]	0.57	975.46	26.41	44	10	559.38	26.80	23	9
**P54466**	UPF0365 protein yqfA OS=Bacillus subtilis GN=yqfA PE=3 SV=1 - [YQFA_BACSU]	1.13	487.84	34.14	23	9	549.19	36.25	28	10
**P96681**	Uncharacterized HTH-type transcriptional regulator ydfD OS=Bacillus subtilis GN=ydfD PE=3 SV=1 - [YDFD_BACSU]	1.69	323.88	18.46	12	7	547.66	21.37	19	8
**P54418**	Phosphoenolpyruvate carboxykinase [ATP] OS=Bacillus subtilis GN=pckA PE=3 SV=3 - [PCKA_BACSU]	0.44	1218.77	31.31	63	14	539.81	26.94	29	12
**O31753**	1-deoxy-D-xylulose 5-phosphate reductoisomerase OS=Bacillus subtilis GN=dxr PE=3 SV=2 - [DXR_BACSU]	0.47	1153.81	41.78	54	14	537.87	39.95	29	14
**P23447**	Flagellar M-ring protein OS=Bacillus subtilis GN=fliF PE=3 SV=2 - [FLIF_BACSU]	2.50	215.23	18.47	16	7	537.68	37.69	21	12
**O31776**	L-threonine 3-dehydrogenase OS=Bacillus subtilis GN=tdh PE=3 SV=1 - [TDH_BACSU]	1.11	483.02	12.39	19	5	536.89	15.85	21	6
**O31656**	Uncharacterized protein ykrK OS=Bacillus subtilis GN=ykrK PE=4 SV=1 - [YKRK_BACSU]	1.52	352.79	43.35	20	10	534.89	43.35	25	10
**P39912**	Protein aroA(G) OS=Bacillus subtilis GN=aroA PE=1 SV=1 - [AROG_BACSU]	0.76	696.45	26.54	30	11	526.11	22.63	24	8
**P50866**	ATP-dependent Clp protease ATP-binding subunit clpX OS=Bacillus subtilis GN=clpX PE=2 SV=3 - [CLPX_BACSU]	0.51	1006.35	37.14	54	16	517.26	26.43	23	10
**O07544**	Endospore coat-associated protein yheC OS=Bacillus subtilis GN=yheC PE=3 SV=1 - [YHEC_BACSU]	0.80	634.40	38.84	41	14	505.70	42.70	38	14
**P25994**	Carbamoyl-phosphate synthase pyrimidine-specific large chain OS=Bacillus subtilis GN=pyrAB PE=3 SV=1 - [CARB_BACSU]	1.30	389.83	13.35	26	15	505.06	20.54	23	18
**P07860**	RNA polymerase sigma-F factor OS=Bacillus subtilis GN=sigF PE=1 SV=1 - [RPSF_BACSU]	#DIV/0!					499.99	21.57	16	6
**P39120**	Citrate synthase 2 OS=Bacillus subtilis GN=citZ PE=1 SV=2 - [CISY2_BACSU]	1.05	466.87	25.54	26	8	492.14	35.75	26	12
**P54420**	Asparagine synthetase [glutamine-hydrolyzing] 1 OS=Bacillus subtilis GN=asnB PE=1 SV=2 - [ASNB_BACSU]	0.70	704.46	25.00	34	14	490.13	19.94	30	10
**P39137**	Amino-acid permease rocE OS=Bacillus subtilis GN=rocE PE=2 SV=1 - [ROCE_BACSU]	1.17	413.88	5.57	17	2	482.52	5.57	15	2
**P37949**	GTP-binding protein lepA OS=Bacillus subtilis GN=lepA PE=3 SV=2 - [LEPA_BACSU]	0.53	899.69	18.95	42	10	480.62	17.81	26	9
**P12877**	50S ribosomal protein L5 OS=Bacillus subtilis GN=rplE PE=3 SV=1 - [RL5_BACSU]	0.53	905.16	58.66	36	9	477.06	53.07	17	8
**P80865**	Succinyl-CoA ligase [ADP-forming] subunit alpha OS=Bacillus subtilis GN=sucD PE=1 SV=3 - [SUCD_BACSU]	1.07	443.83	28.33	20	6	473.89	25.00	20	5
**P38424**	Probable GTP-binding protein engB OS=Bacillus subtilis GN=engB PE=1 SV=1 - [ENGB_BACSU]	1.12	419.92	58.46	25	9	470.44	35.90	24	8
**P68732**	Replication termination protein OS=Bacillus subtilis GN=rtp PE=1 SV=1 - [RTP_BACSU]	1.21	385.24	40.98	21	5	464.60	40.98	21	5
**P39634**	1-pyrroline-5-carboxylate dehydrogenase OS=Bacillus subtilis GN=rocA PE=2 SV=1 - [ROCA_BACSU]	0.54	858.40	36.89	53	14	464.14	36.12	24	12
**P21882**	Pyruvate dehydrogenase E1 component subunit beta OS=Bacillus subtilis GN=pdhB PE=3 SV=2 - [ODPB_BACSU]	0.65	709.00	24.62	29	7	457.49	24.62	17	7
**P09339**	Aconitate hydratase OS=Bacillus subtilis GN=citB PE=1 SV=4 - [ACON_BACSU]	0.93	489.06	18.15	30	13	455.74	22.11	19	14
**P96591**	Putative thiamine pyrophosphate-containing protein ydaP OS=Bacillus subtilis GN=ydaP PE=2 SV=1 - [YDAP_BACSU]	1.61	283.39	15.33	12	6	455.55	28.05	24	12
**Q45480**	Uncharacterized RNA pseudouridine synthase ylyB OS=Bacillus subtilis GN=ylyB PE=3 SV=3 - [YLYB_BACSU]	1.68	271.24	27.39	13	7	454.78	30.36	27	8
**P40750**	Penicillin-binding protein 4 OS=Bacillus subtilis GN=pbpD PE=1 SV=2 - [PBPD_BACSU]	0.80	560.44	24.36	36	12	448.25	23.40	24	12
**O34693**	Uncharacterized protein yloA OS=Bacillus subtilis GN=yloA PE=2 SV=1 - [YLOA_BACSU]	0.72	612.27	27.45	31	17	440.11	27.45	24	17

**O34433**	Putative phage-related protein yobO OS=Bacillus subtilis GN=yobO PE=4 SV=1 - [YOBO_BACSU]	1.03	427.62	10.17	20	6	438.47	11.17	15	6
**O31645**	PTS system mannose-specific EIIBCA component OS=Bacillus subtilis GN=manP PE=1 SV=2 - [PTN3B_BACSU]	0.60	718.92	21.85	38	13	431.81	26.92	29	13
**Q796K8**	Penicillin-binding protein H OS=Bacillus subtilis GN=pbpH PE=1 SV=2 - [PBPH_BACSU]	3.41	126.50	8.24	11	4	431.33	19.18	21	9
**O06714**	Nuclease sbcCD subunit C OS=Bacillus subtilis GN=sbcC PE=3 SV=3 - [SBCC_BACSU]	1.00	425.99	23.98	40	26	427.44	13.98	22	14
**P54533**	Dihydrolipoyl dehydrogenase OS=Bacillus subtilis GN=bfmBC PE=3 SV=1 - [DLDH2_BACSU]	0.52	828.59	31.22	47	14	426.89	25.74	18	12
**Q01464**	Septum site-determining protein minD OS=Bacillus subtilis GN=minD PE=3 SV=1 - [MIND_BACSU]	1.16	359.48	50.75	22	12	415.45	44.40	20	10
**Q07876**	S-adenosyl-L-methionine-dependent methyltransferase mraW OS=Bacillus subtilis GN=mraW PE=3 SV=2 - [MRAW_BACSU]	0.76	541.80	22.19	19	5	410.79	27.65	21	7
**O34847**	Acetyl-coenzyme A carboxylase carboxyl transferase subunit alpha OS=Bacillus subtilis GN=accA PE=1 SV=1 - [ACCA_BACSU]	0.89	460.46	32.31	23	9	410.65	38.15	15	11
**P39844**	D-alanyl-D-alanine carboxypeptidase dacC OS=Bacillus subtilis GN=dacC PE=1 SV=1 - [DACC_BACSU]	0.56	732.04	26.88	31	10	409.69	33.60	16	11
**P96714**	Uncharacterized protein ywqB OS=Bacillus subtilis GN=ywqB PE=4 SV=2 - [YWQB_BACSU]	5.93	68.58	6.90	5	4	406.58	25.56	24	12
**P71007**	Antilisterial bacteriocin subtilosin biosynthesis protein albE OS=Bacillus subtilis GN=albE PE=2 SV=1 - [ALBE1_BACSU]	1.33	300.74	28.24	15	8	400.99	31.61	20	11
**P54453**	Uncharacterized protein yqeH OS=Bacillus subtilis GN=yqeH PE=4 SV=1 - [YQEH_BACSU]	1.69	236.66	17.21	10	5	399.91	21.58	15	6
**P18255**	Threonyl-tRNA synthetase 1 OS=Bacillus subtilis GN=thrS PE=2 SV=1 - [SYT1_BACSU]	1.09	366.04	14.00	17	8	397.31	10.73	13	6
**P54571**	Malate-2H(+)/Na(+)-lactate antiporter OS=Bacillus subtilis GN=mleN PE=1 SV=1 - [MLEN_BACSU]	2.03	195.36	10.04	8	4	396.61	8.33	13	3
**P94360**	Maltodextrin import ATP-binding protein msmX OS=Bacillus subtilis GN=msmX PE=3 SV=1 - [MSMX_BACSU]	0.87	450.07	27.40	21	8	393.01	19.18	20	6
**P94545**	MutS2 protein OS=Bacillus subtilis GN=mutSB PE=3 SV=2 - [MUTS2_BACSU]	0.78	500.46	18.47	35	13	392.50	15.80	18	12
**P42958**	Probable tartrate dehydrogenase/decarboxylase OS=Bacillus subtilis GN=ycsA PE=3 SV=4 - [TTUC_BACSU]	0.86	453.90	21.47	14	6	390.96	28.81	11	8
**P37814**	ATP synthase subunit b OS=Bacillus subtilis GN=atpF PE=3 SV=1 - [ATPF_BACSU]	2.82	138.28	41.76	9	7	390.28	48.82	15	9
**P21468**	30S ribosomal protein S6 OS=Bacillus subtilis GN=rpsF PE=1 SV=2 - [RS6_BACSU]	1.35	289.75	49.47	13	6	389.79	49.47	13	5
**P35150**	D-alanyl-D-alanine carboxypeptidase dacB OS=Bacillus subtilis GN=dacB PE=1 SV=1 - [DACB_BACSU]	1.19	325.75	24.08	21	8	387.81	21.73	16	7
**P71067**	L-lactate permease OS=Bacillus subtilis GN=lutP PE=1 SV=2 - [LUTP_BACSU]	2.77	138.29	7.10	4	3	383.47	7.10	10	3
**P08065**	Succinate dehydrogenase flavoprotein subunit OS=Bacillus subtilis GN=sdhA PE=3 SV=4 - [DHSA_BACSU]	1.19	317.38	18.60	17	9	378.22	27.65	21	13
**P42182**	GTP-binding protein era homolog OS=Bacillus subtilis GN=era PE=3 SV=1 - [ERA_BACSU]	3.06	123.26	13.29	8	3	377.35	20.27	17	6
**O31773**	Putative penicillin-binding protein pbpX OS=Bacillus subtilis GN=pbpX PE=2 SV=1 - [PBPX_BACSU]	1.57	239.16	18.67	17	6	375.98	17.65	20	5
**O32162**	FeS cluster assembly protein sufB OS=Bacillus subtilis GN=sufB PE=3 SV=1 - [SUFB_BACSU]	0.56	674.34	23.01	34	9	375.65	21.08	13	8
**P80698**	Trigger factor OS=Bacillus subtilis GN=tig PE=1 SV=3 - [TIG_BACSU]	0.62	593.27	28.07	30	11	368.00	26.18	13	9
**P06224**	RNA polymerase sigma factor rpoD OS=Bacillus subtilis GN=sigA PE=1 SV=2 - [RPOD_BACSU]	4.00	91.14	9.43	5	3	364.48	28.57	18	9
**Q03224**	Uncharacterized protein ywjI OS=Bacillus subtilis GN=ywjI PE=3 SV=1 - [YWJI_BACSU]	0.63	575.16	27.41	24	8	359.96	27.10	13	7

**P37544**	UPF0011 protein yabC OS=Bacillus subtilis GN=yabC PE=3 SV=1 - [YABC_BACSU]	1.60	223.90	14.73	14	5	358.72	14.73	20	5
**P08838**	Phosphoenolpyruvate-protein phosphotransferase OS=Bacillus subtilis GN=ptsI PE=1 SV=3 - [PT1_BACSU]	0.83	429.31	20.53	23	11	356.22	23.51	14	10
**P45694**	Transketolase OS=Bacillus subtilis GN=tkt PE=3 SV=2 - [TKT_BACSU]	0.42	835.65	28.19	45	14	351.42	17.84	14	8
**Q06753**	Putative trmH family tRNA/rRNA methyltransferase yacO OS=Bacillus subtilis GN=yacO PE=3 SV=2 - [YACO_BACSU]	0.91	379.15	40.56	16	9	346.67	43.37	22	10
**P25144**	Catabolite control protein A OS=Bacillus subtilis GN=ccpA PE=1 SV=1 - [CCPA_BACSU]	0.67	518.29	29.94	29	8	345.51	21.86	19	6
**O32006**	Resolvase homolog yokA OS=Bacillus subtilis GN=yokA PE=3 SV=1 - [YOKA_BACSU]	3.01	114.36	10.64	8	4	344.78	30.46	17	12
**P40924**	Phosphoglycerate kinase OS=Bacillus subtilis GN=pgk PE=1 SV=3 - [PGK_BACSU]	0.59	581.13	28.68	29	9	342.87	23.60	16	7
**P31112**	Heptaprenyl diphosphate synthase component 1 OS=Bacillus subtilis GN=hepS PE=1 SV=1 - [HEPS1_BACSU]	2.14	159.59	16.33	7	5	342.14	23.51	14	7
**P46919**	Glycerol-3-phosphate dehydrogenase [NAD(P)+] OS=Bacillus subtilis GN=gpsA PE=3 SV=2 - [GPDA_BACSU]	1.30	261.03	25.80	12	7	338.38	28.12	13	7
**P37869**	Enolase OS=Bacillus subtilis GN=eno PE=1 SV=4 - [ENO_BACSU]	0.34	1004.37	27.91	41	12	337.30	25.12	16	10
**Q07868**	Penicillin-binding protein 2B OS=Bacillus subtilis GN=pbpB PE=2 SV=2 - [PBPB_BACSU]	1.28	263.21	17.88	20	10	336.46	17.46	18	10
**P46352**	Tyrosine recombinase xerD OS=Bacillus subtilis GN=xerD PE=3 SV=3 - [XERD_BACSU]	0.87	383.47	40.54	20	10	334.50	33.78	12	9
**O06478**	Putative aldehyde dehydrogenase yfmT OS=Bacillus subtilis GN=yfmT PE=2 SV=1 - [ALDH5_BACSU]	0.54	616.51	29.69	32	14	331.21	27.84	17	10
**P54482**	4-hydroxy-3-methylbut-2-en-1-yl diphosphate synthase OS=Bacillus subtilis GN=ispG PE=3 SV=1 - [ISPG_BACSU]	0.61	534.79	22.55	17	7	326.07	27.06	16	8
**P54382**	Bifunctional protein folD OS=Bacillus subtilis GN=folD PE=3 SV=1 - [FOLD_BACSU]	1.64	198.21	20.85	11	7	325.24	22.26	13	7
**P80885**	Pyruvate kinase OS=Bacillus subtilis GN=pyk PE=1 SV=2 - [KPYK_BACSU]	0.82	385.03	17.26	20	8	316.79	24.27	19	10
**P39127**	HTH-type transcriptional regulator citR OS=Bacillus subtilis GN=citR PE=4 SV=2 - [CITR_BACSU]	1.61	196.22	19.93	11	5	316.73	27.84	15	7
**O31501**	Swarming motility protein swrC OS=Bacillus subtilis GN=swrC PE=2 SV=2 - [SWRC_BACSU]	0.85	373.24	7.13	18	7	316.37	6.18	18	5
**P55343**	Protein mraZ OS=Bacillus subtilis GN=mraZ PE=3 SV=1 - [MRAZ_BACSU]	1.36	232.25	45.45	18	7	315.76	45.45	16	6
**P46344**	Uncharacterized protein yqfF OS=Bacillus subtilis GN=yqfF PE=4 SV=2 - [YQFF_BACSU]	0.57	556.08	19.83	35	14	314.75	16.17	16	9
**P18158**	Aerobic glycerol-3-phosphate dehydrogenase OS=Bacillus subtilis GN=glpD PE=2 SV=2 - [GLPD_BACSU]	0.77	407.24	29.73	29	15	313.88	28.11	23	15
**P21466**	30S ribosomal protein S4 OS=Bacillus subtilis GN=rpsD PE=1 SV=3 - [RS4_BACSU]	0.80	381.81	45.50	22	9	307.18	41.50	23	8
**O07581**	Probable anti-sigma-M factor yhdL OS=Bacillus subtilis GN=yhdL PE=1 SV=1 - [YHDL_BACSU]	2.61	117.68	17.60	6	5	307.16	19.55	12	6
**O31423**	Uncharacterized protein skfB OS=Bacillus subtilis GN=skfB PE=2 SV=2 - [SKFB_BACSU]	0.77	395.66	11.71	17	4	306.07	12.93	16	6
**P19582**	Homoserine dehydrogenase OS=Bacillus subtilis GN=hom PE=3 SV=2 - [DHOM_BACSU]	1.11	274.62	14.78	10	5	304.91	23.56	14	7
**P54394**	Probable ATP-dependent helicase dinG homolog OS=Bacillus subtilis GN=dinG PE=3 SV=1 - [DING_BACSU]	6.38	47.64	3.87	4	3	303.92	19.01	17	12
**P21881**	Pyruvate dehydrogenase E1 component subunit alpha OS=Bacillus subtilis GN=pdhA PE=1 SV=3 - [ODPA_BACSU]	0.48	638.52	36.93	27	11	303.78	29.11	21	10
**P24011**	Cytochrome c oxidase subunit 2 OS=Bacillus subtilis GN=ctaC PE=3 SV=2 - [COX2_BACSU]	1.47	205.90	17.13	12	6	302.83	16.29	10	6

**O31774**	2',3'-cyclic-nucleotide 2'-phosphodiesterase OS=Bacillus subtilis GN=ymdA PE=3 SV=1 - [CNPD_BACSU]	0.81	370.46	20.19	20	11	298.86	15.19	14	8
**O31755**	Prolyl-tRNA synthetase OS=Bacillus subtilis GN=proS PE=3 SV=1 - [SYP_BACSU]	0.49	607.62	28.90	26	14	297.57	13.48	15	6
**P13243**	Probable fructose-bisphosphate aldolase OS=Bacillus subtilis GN=fbaA PE=1 SV=2 - [ALF_BACSU]	0.44	670.97	28.42	34	8	294.55	31.23	18	8
**P96499**	Putative transcriptional regulator yvhJ OS=Bacillus subtilis GN=yvhJ PE=3 SV=1 - [YVHJ_BACSU]	0.59	488.90	20.97	25	8	290.27	22.76	14	8
**O31689**	Putative dipeptidase ykvY OS=Bacillus subtilis GN=ykvY PE=3 SV=1 - [YKVY_BACSU]	0.93	311.77	23.42	13	7	290.14	26.45	12	7
**P45858**	2-methylcitrate synthase OS=Bacillus subtilis GN=mmgD PE=1 SV=1 - [CISY3_BACSU]	0.57	507.79	34.68	23	10	287.38	25.81	14	8
**O31743**	Ribosome biogenesis GTPase A OS=Bacillus subtilis GN=rbgA PE=1 SV=1 - [RBGA_BACSU]	1.28	225.00	23.76	12	6	287.38	31.21	14	9
**P21469**	30S ribosomal protein S7 OS=Bacillus subtilis GN=rpsG PE=1 SV=4 - [RS7_BACSU]	0.75	380.57	43.59	14	6	287.20	44.23	12	7
**P34956**	Quinol oxidase subunit 1 OS=Bacillus subtilis GN=qoxB PE=1 SV=1 - [QOX1_BACSU]	0.94	301.54	4.78	22	4	282.77	6.32	14	4
**P13485**	CDP-glycerol:poly(glycerophosphate) glycerophosphotransferase OS=Bacillus subtilis GN=tagF PE=1 SV=1 - [TAGF_BACSU]	0.87	322.91	16.62	24	12	281.84	17.29	19	12
**O31716**	Uncharacterized ABC transporter ATP-binding protein ykpA OS=Bacillus subtilis GN=ykpA PE=3 SV=1 - [YKPA_BACSU]	0.61	462.08	29.26	22	12	281.71	25.74	18	11
**P12011**	Gluconokinase OS=Bacillus subtilis GN=gntK PE=3 SV=1 - [GNTK_BACSU]	0.50	553.80	26.32	25	11	277.97	15.01	10	7
**Q04796**	Dihydrodipicolinate synthase OS=Bacillus subtilis GN=dapA PE=1 SV=1 - [DAPA_BACSU]	0.56	495.20	34.48	23	7	277.29	40.69	16	9
**O34526**	Alanyl-tRNA synthetase OS=Bacillus subtilis GN=alaS PE=3 SV=1 - [SYA_BACSU]	0.83	330.51	19.13	22	14	275.66	10.82	9	7
**O32258**	Uncharacterized glycosylase yvbX OS=Bacillus subtilis GN=yvbX PE=3 SV=1 - [YVBX_BACSU]	0.89	306.02	14.24	14	4	273.74	21.22	17	6
**O32164**	Probable cysteine desulfurase OS=Bacillus subtilis GN=csd PE=3 SV=1 - [CSD_BACSU]	0.44	620.05	24.63	31	8	269.73	9.11	7	3
**Q795M6**	Putative aminotransferase yugH OS=Bacillus subtilis GN=yugH PE=3 SV=1 - [YUGH_BACSU]	0.65	414.23	22.80	18	6	268.61	24.35	11	6
**P37570**	Putative ATP:guanido phosphotransferase yacI OS=Bacillus subtilis GN=yacI PE=3 SV=1 - [YACI_BACSU]	1.04	253.19	22.31	14	8	264.24	19.83	14	7
**P26497**	Stage 0 sporulation protein J OS=Bacillus subtilis GN=spo0J PE=1 SV=2 - [SP0J_BACSU]	1.11	237.20	36.17	10	9	263.16	29.79	13	10
**P37487**	Manganese-dependent inorganic pyrophosphatase OS=Bacillus subtilis GN=ppaC PE=1 SV=1 - [PPAC_BACSU]	0.65	402.21	26.21	12	7	260.36	14.89	7	3
**P42435**	Nitrite reductase [NAD(P)H] OS=Bacillus subtilis GN=nasD PE=2 SV=1 - [NASD_BACSU]	1.12	230.32	10.56	16	6	258.05	18.01	15	10
**P54604**	Uncharacterized RNA pseudouridine synthase yhcT OS=Bacillus subtilis GN=yhcT PE=3 SV=2 - [YHCT_BACSU]	0.77	329.79	23.18	11	5	254.66	26.49	8	6
**P05649**	DNA polymerase III subunit beta OS=Bacillus subtilis GN=dnaN PE=3 SV=1 - [DPO3B_BACSU]	0.61	414.87	23.54	18	7	253.09	20.90	13	6
**P12042**	Phosphoribosylformylglycinamidine synthase 2 OS=Bacillus subtilis GN=purL PE=3 SV=2 - [PURL_BACSU]	2.15	116.54	8.63	10	5	250.93	20.62	16	9
**P39217**	Methyl-accepting chemotaxis protein tlpB OS=Bacillus subtilis GN=tlpB PE=3 SV=1 - [TLPB_BACSU]	0.80	310.53	12.39	16	8	248.13	10.42	11	6
**O34525**	Putative signal peptide peptidase sppA OS=Bacillus subtilis GN=sppA PE=3 SV=1 - [SPPA_BACSU]	0.88	281.29	9.55	10	2	247.58	9.55	6	2
**P94551**	Electron transfer flavoprotein subunit alpha OS=Bacillus subtilis GN=etfA PE=3 SV=1 - [ETFA_BACSU]	1.08	229.80	24.00	12	6	247.34	24.00	11	6
**O31749**	Uridylate kinase OS=Bacillus subtilis GN=pyrH PE=1 SV=2 - [PYRH_BACSU]	1.24	199.28	25.00	8	5	246.94	19.17	10	4

**P45745**	Dimodular nonribosomal peptide synthase OS=Bacillus subtilis GN=dhbF PE=1 SV=4 - [DHBF_BACSU]	0.69	355.52	8.03	31	16	244.07	7.28	18	13
**P68579**	SPBc2 prophage-derived sublancin-168-processing and transport ATP-binding protein sunT OS=Bacillus subtilis GN=sunT PE=3 SV=1 - [SUNT_BACSU]	0.33	743.12	19.57	36	12	242.12	15.46	18	11
**P17869**	RNA polymerase sigma-H factor OS=Bacillus subtilis GN=sigH PE=1 SV=1 - [RPSH_BACSU]	8.57	27.93	9.17	2	2	239.35	35.78	10	7
**O34942**	ATP-dependent DNA helicase recG OS=Bacillus subtilis GN=recG PE=3 SV=1 - [RECG_BACSU]	2.17	110.24	4.40	7	3	238.88	5.72	10	3
**P05652**	DNA gyrase subunit B OS=Bacillus subtilis GN=gyrB PE=3 SV=1 - [GYRB_BACSU]	1.19	200.72	9.56	14	6	237.89	17.24	14	9
**P54488**	Uncharacterized protein yqgF OS=Bacillus subtilis GN=yqgF PE=3 SV=1 - [YQGF_BACSU]	0.98	241.28	10.20	11	6	237.26	17.46	17	10
**Q08787**	Surfactin synthetase subunit 3 OS=Bacillus subtilis GN=srfAC PE=1 SV=2 - [SRFAC_BACSU]	1.03	228.79	8.39	21	9	236.07	11.06	14	10
**O34909**	Putative adenine deaminase yerA OS=Bacillus subtilis GN=yerA PE=1 SV=1 - [YERA_BACSU]	0.73	320.95	14.83	21	9	235.87	12.59	12	7
**P37503**	Uncharacterized HTH-type transcriptional regulator yybA OS=Bacillus subtilis GN=yybA PE=4 SV=1 - [YYBA_BACSU]	0.97	239.72	44.67	10	6	233.45	51.33	10	8
**P51835**	Cell division protein ftsY homolog OS=Bacillus subtilis GN=ftsY PE=1 SV=2 - [FTSY_BACSU]	0.78	300.29	22.80	16	6	233.16	22.80	12	6
**O06734**	AB hydrolase superfamily protein yisY OS=Bacillus subtilis GN=yisY PE=3 SV=1 - [YISY_BACSU]	0.78	297.02	31.72	14	7	231.62	32.46	14	9
**P42974**	NADH dehydrogenase OS=Bacillus subtilis GN=ahpF PE=1 SV=2 - [DHNA_BACSU]	0.44	525.28	20.24	26	9	228.96	16.11	11	7
**O34758**	UPF0755 protein yrrL OS=Bacillus subtilis GN=yrrL PE=3 SV=1 - [YRRL_BACSU]	1.28	179.09	24.17	14	7	228.41	25.28	12	9
**O31661**	Sporulation kinase E OS=Bacillus subtilis GN=kinE PE=1 SV=1 - [KINE_BACSU]	1.31	174.02	6.10	11	4	228.38	10.57	14	6
**P94544**	Uncharacterized protein yshC OS=Bacillus subtilis GN=yshC PE=3 SV=1 - [YSHC_BACSU]	#DIV/0!					227.82	17.89	9	8
**O32076**	Uncharacterized protein yuaG OS=Bacillus subtilis GN=yuaG PE=4 SV=1 - [YUAG_BACSU]	0.60	378.75	20.83	17	9	226.30	12.77	7	5
**P31102**	3-dehydroquinate synthase OS=Bacillus subtilis GN=aroB PE=3 SV=1 - [AROB_BACSU]	0.70	321.01	25.41	21	8	223.36	24.03	15	7
**O34325**	Uncharacterized protein ytrP OS=Bacillus subtilis GN=ytrP PE=4 SV=1 - [YTRP_BACSU]	2.15	103.19	0.00	3	2	221.71	6.22	6	3
**Q02115**	Transcriptional regulator lytR OS=Bacillus subtilis GN=lytR PE=1 SV=1 - [LYAT_BACSU]	1.72	129.15	12.09	8	3	221.59	16.34	12	4
**P54377**	Probable glycine dehydrogenase [decarboxylating] subunit 2 OS=Bacillus subtilis GN=gcvPB PE=3 SV=1 - [GCSPB_BACSU]	0.85	260.39	17.62	14	6	221.22	17.62	10	6
**P37585**	UDP-N-acetylglucosamine--N-acetylmuramyl-(pentapeptide) pyrophosphoryl-undecaprenol N-acetylglucosamine transferase OS=Bacillus subtilis GN=murG PE=3 SV=3 - [MURG_BACSU]	0.93	237.58	19.01	14	6	219.87	21.49	12	7
**P70965**	UDP-N-acetylglucosamine 1-carboxyvinyltransferase 1 OS=Bacillus subtilis GN=murAA PE=1 SV=1 - [MURA1_BACSU]	0.27	802.11	34.17	35	9	219.65	6.19	6	2
**P37527**	Pyridoxal biosynthesis lyase pdxS OS=Bacillus subtilis GN=pdxS PE=1 SV=3 - [PDXS_BACSU]	0.45	490.12	28.23	26	8	219.29	24.49	12	6
**Q05470**	Polyketide synthase pksL OS=Bacillus subtilis GN=pksL PE=1 SV=3 - [PKSL_BACSU]	1.30	168.38	3.13	12	12	218.50	3.77	19	14
**P46336**	Protein iolS OS=Bacillus subtilis GN=iolS PE=1 SV=1 - [IOLS_BACSU]	0.96	225.54	28.71	13	7	217.33	22.58	9	5
**O07021**	Lactate utilization protein B OS=Bacillus subtilis GN=lutB PE=1 SV=2 - [LUTB_BACSU]	0.39	550.13	17.12	22	7	214.30	7.31	9	3
**P53001**	Aspartate aminotransferase OS=Bacillus subtilis GN=aspB PE=3 SV=1 - [AAT1_BACSU]	0.63	337.56	25.95	14	7	213.27	29.77	12	9
**P49850**	DNA mismatch repair protein mutL OS=Bacillus subtilis GN=mutL PE=3 SV=1 - [MUTL_BACSU]	0.82	255.00	8.93	10	5	209.55	12.92	9	6

**P39148**	Serine hydroxymethyltransferase OS=Bacillus subtilis GN=glyA PE=3 SV=1 - [GLYA_BACSU]	0.44	468.18	27.47	18	9	207.10	13.25	9	4
**P42084**	Imidazolonepropionase OS=Bacillus subtilis GN=hutI PE=1 SV=1 - [HUTI_BACSU]	0.86	239.25	14.49	7	4	204.71	14.96	10	4
**P50621**	Ribonucleoside-diphosphate reductase subunit beta OS=Bacillus subtilis GN=nrdF PE=3 SV=1 - [RIR2_BACSU]	0.83	246.63	17.63	14	5	204.45	17.33	17	6
**P13800**	Transcriptional regulatory protein degU OS=Bacillus subtilis GN=degU PE=3 SV=2 - [DEGU_BACSU]	0.66	309.33	37.12	13	7	204.41	32.75	14	7
**P39760**	Ktr system potassium uptake protein C OS=Bacillus subtilis GN=ktrC PE=3 SV=1 - [KTRC_BACSU]	0.76	268.14	28.05	15	6	203.33	29.86	12	7
**P46337**	HTH-type transcriptional regulator iolR OS=Bacillus subtilis GN=iolR PE=4 SV=1 - [IOLR_BACSU]	2.10	96.58	20.32	4	4	202.59	33.47	10	8
**O05518**	Probable O-sialoglycoprotein endopeptidase OS=Bacillus subtilis GN=gcp PE=3 SV=1 - [GCP_BACSU]	0.59	342.95	19.08	14	5	201.90	10.40	7	3
**Q7WY72**	UPF0296 protein ylzA OS=Bacillus subtilis GN=ylzA PE=3 SV=1 - [YLZA_BACSU]	0.71	281.37	59.55	13	6	199.87	43.82	11	5
**O34949**	Uncharacterized HTH-type transcriptional regulator ykoM OS=Bacillus subtilis GN=ykoM PE=4 SV=1 - [YKOM_BACSU]	0.60	328.28	42.21	15	7	198.49	25.32	8	5
**P49814**	Malate dehydrogenase OS=Bacillus subtilis GN=mdh PE=1 SV=3 - [MDH_BACSU]	0.65	303.55	16.67	13	5	197.96	15.06	8	3
**Q06797**	50S ribosomal protein L1 OS=Bacillus subtilis GN=rplA PE=3 SV=4 - [RL1_BACSU]	0.51	388.89	33.62	17	8	196.48	28.88	7	6
**O34996**	DNA polymerase I OS=Bacillus subtilis GN=polA PE=3 SV=1 - [DPO1_BACSU]	2.52	76.59	7.05	10	6	192.81	9.66	7	6
**P54545**	DNA polymerase IV 1 OS=Bacillus subtilis GN=dinB1 PE=3 SV=2 - [DPO41_BACSU]	3.40	56.50	9.18	3	3	192.02	13.53	8	5
**P23453**	Flagellar motor switch protein fliM OS=Bacillus subtilis GN=fliM PE=3 SV=1 - [FLIM_BACSU]	0.45	429.33	22.29	18	7	191.99	18.98	10	5
**P21474**	30S ribosomal protein S16 OS=Bacillus subtilis GN=rpsP PE=1 SV=3 - [RS16_BACSU]	0.88	215.37	57.78	11	4	190.59	46.67	9	3
**P39776**	Tyrosine recombinase xerC OS=Bacillus subtilis GN=xerC PE=1 SV=1 - [XERC_BACSU]	2.86	66.60	8.22	2	2	190.59	21.71	9	5
**O31742**	50S ribosomal protein L19 OS=Bacillus subtilis GN=rplS PE=3 SV=2 - [RL19_BACSU]	0.90	210.63	39.13	11	5	189.28	39.13	13	5
**O31727**	UPF0001 protein ylmE OS=Bacillus subtilis GN=ylmE PE=3 SV=1 - [YLME_BACSU]	0.83	227.58	27.39	6	5	189.27	34.35	8	6
**C0SP89**	Putative methyl-accepting chemotaxis protein yoaH OS=Bacillus subtilis GN=yoaH PE=2 SV=1 - [YOAH_BACSU]	2.70	69.86	4.46	2	2	188.89	4.63	3	2
**P29727**	GMP synthase [glutamine-hydrolyzing] OS=Bacillus subtilis GN=guaA PE=3 SV=3 - [GUAA_BACSU]	0.59	318.31	14.81	20	7	188.72	14.62	6	6
**P21885**	Arginine decarboxylase OS=Bacillus subtilis GN=speA PE=1 SV=2 - [SPEA_BACSU]	0.56	332.83	19.39	20	9	186.96	14.90	9	6
**P16397**	Bacillopeptidase F OS=Bacillus subtilis GN=bpr PE=1 SV=2 - [SUBF_BACSU]	3.91	47.70	2.51	4	3	186.62	5.09	9	5
**O34829**	Uncharacterized HTH-type transcriptional regulator msmR OS=Bacillus subtilis GN=msmR PE=4 SV=1 - [MSMR_BACSU]	#DIV/0!					185.80	7.56	4	2
**O31575**	Regulatory protein recX OS=Bacillus subtilis GN=recX PE=3 SV=1 - [RECX_BACSU]	0.98	187.95	24.24	11	8	183.95	24.24	13	8
**O07020**	Lactate utilization protein A OS=Bacillus subtilis GN=lutA PE=1 SV=1 - [LUTA_BACSU]	0.80	228.61	18.49	9	4	183.31	23.95	9	5
**P21879**	Inosine-5'-monophosphate dehydrogenase OS=Bacillus subtilis GN=guaB PE=1 SV=2 - [IMDH_BACSU]	0.33	544.20	18.03	21	8	181.96	9.22	7	4
**P80866**	Vegetative protein 296 OS=Bacillus subtilis GN=yurY PE=1 SV=3 - [V296_BACSU]	0.49	370.52	55.17	22	12	181.49	27.59	10	7
**P71012**	PTS system fructose-specific EIIABC component OS=Bacillus subtilis GN=fruA PE=1 SV=2 - [PTF3A_BACSU]	0.60	301.21	12.76	17	5	180.19	11.02	9	4

**P30300**	Glycerol uptake operon antiterminator regulatory protein OS=Bacillus subtilis GN=glpP PE=4 SV=1 - [GLPP_BACSU]	0.78	230.73	29.17	11	5	179.84	29.17	8	5
**P37551**	Pur operon repressor OS=Bacillus subtilis GN=purR PE=1 SV=1 - [PURR_BACSU]	1.05	170.33	19.30	9	5	178.83	18.25	9	5
**P42064**	Oligopeptide transport ATP-binding protein appD OS=Bacillus subtilis GN=appD PE=3 SV=1 - [APPD_BACSU]	1.96	91.36	12.20	5	3	178.70	15.85	6	4
**P35149**	Stage IV sporulation protein A OS=Bacillus subtilis GN=spoIVA PE=1 SV=1 - [SP4A_BACSU]	0.83	213.16	14.02	14	6	176.90	14.02	9	6
**Q45577**	Probable amino acid-proton symporter ybeC OS=Bacillus subtilis GN=ybeC PE=3 SV=3 - [YBEC_BACSU]	1.05	167.82	3.34	6	2	175.56	0.00	5	1
**O31852**	D-gamma-glutamyl-meso-diaminopimelic acid endopeptidase cwlS OS=Bacillus subtilis GN=cwlS PE=1 SV=1 - [CWLS_BACSU]	3.70	47.20	9.18	3	3	174.88	11.59	6	4
**P17922**	Phenylalanyl-tRNA synthetase beta chain OS=Bacillus subtilis GN=pheT PE=3 SV=2 - [SYFB_BACSU]	0.53	328.44	15.80	16	12	174.17	6.47	5	4
**P39067**	Acetoin utilization protein acuC OS=Bacillus subtilis GN=acuC PE=3 SV=1 - [ACUC_BACSU]	1.30	132.82	9.04	7	3	173.04	5.94	5	2
**P19406**	Alkaline phosphatase 4 OS=Bacillus subtilis GN=phoA PE=1 SV=4 - [PPB4_BACSU]	0.29	598.80	32.54	32	12	172.47	18.44	9	6
**P37477**	Lysyl-tRNA synthetase OS=Bacillus subtilis GN=lysS PE=3 SV=1 - [SYK_BACSU]	0.63	271.91	15.23	14	7	170.65	15.03	7	6
**P20166**	PTS system glucose-specific EIICBA component OS=Bacillus subtilis GN=ptsG PE=1 SV=2 - [PTG3C_BACSU]	0.41	411.09	10.59	23	7	170.26	14.16	16	9
**P94541**	Ribonuclease HIII OS=Bacillus subtilis GN=rnhC PE=1 SV=2 - [RNH3_BACSU]	2.10	80.32	10.86	9	3	168.96	13.74	11	4
**O34309**	Putative phosphoenolpyruvate synthase OS=Bacillus subtilis GN=pps PE=3 SV=1 - [PPS_BACSU]	1.30	129.48	7.27	9	6	168.86	11.78	10	8
**O32169**	Methionine import ATP-binding protein metN OS=Bacillus subtilis GN=metN PE=1 SV=1 - [METN_BACSU]	0.89	189.15	9.09	6	2	168.29	23.17	10	6
**P37557**	Uncharacterized protein yabO OS=Bacillus subtilis GN=yabO PE=4 SV=1 - [YABO_BACSU]	0.81	207.95	46.51	11	5	167.73	46.51	8	5
**O31718**	UPF0356 protein ykzG OS=Bacillus subtilis GN=ykzG PE=3 SV=1 - [YKZG_BACSU]	#DIV/0!					167.02	39.13	7	4
**O34962**	Probable NAD-dependent malic enzyme 4 OS=Bacillus subtilis GN=ytsJ PE=3 SV=1 - [MAO4_BACSU]	0.73	228.53	13.66	9	4	166.81	19.76	9	6
**O34857**	Repressor rok OS=Bacillus subtilis GN=rok PE=1 SV=1 - [ROK_BACSU]	0.81	204.34	29.84	9	5	165.53	20.94	7	5
**Q45597**	Fructose-1,6-bisphosphatase class 3 OS=Bacillus subtilis GN=fbp PE=1 SV=2 - [F16PC_BACSU]	0.91	181.70	13.26	16	10	165.46	13.88	11	8
**O32218**	Disulfide bond formation protein D OS=Bacillus subtilis GN=bdbD PE=1 SV=1 - [BDBD_BACSU]	0.66	250.27	40.09	11	7	163.96	27.93	7	5
**P94356**	Uncharacterized protein yxkC OS=Bacillus subtilis GN=yxkC PE=1 SV=2 - [YXKC_BACSU]	0.96	169.67	38.33	10	8	163.24	41.11	8	7
**O07532**	Endopeptidase lytF OS=Bacillus subtilis GN=lytF PE=1 SV=2 - [LYTF_BACSU]	#DIV/0!					162.12	12.50	7	4
**P94403**	HTH-type transcriptional regulator bsdA OS=Bacillus subtilis GN=bsdA PE=4 SV=1 - [BSDA_BACSU]	1.58	102.67	7.59	3	2	162.03	20.00	14	6
**O32023**	Uncharacterized protein yqzC OS=Bacillus subtilis GN=yqzC PE=4 SV=2 - [YQZC_BACSU]	0.88	183.82	25.97	6	4	162.00	18.18	4	2
**P94591**	HTH-type transcriptional repressor glcR OS=Bacillus subtilis GN=glcR PE=1 SV=2 - [GLCR_BACSU]	#DIV/0!					161.04	27.52	10	7
**P04990**	Threonine synthase OS=Bacillus subtilis GN=thrC PE=3 SV=1 - [THRC_BACSU]	0.82	195.52	11.08	5	3	160.76	7.95	3	2
**P94547**	Long-chain-fatty-acid--CoA ligase OS=Bacillus subtilis GN=lcfA PE=3 SV=1 - [LCFA_BACSU]	0.94	171.18	6.61	11	3	160.52	10.36	8	4
**P46898**	50S ribosomal protein L6 OS=Bacillus subtilis GN=rplF PE=3 SV=2 - [RL6_BACSU]	0.79	199.63	27.37	9	5	158.27	32.96	8	5

**P71084**	Glutamate-1-semialdehyde 2,1-aminomutase 2 OS=Bacillus subtilis GN=gsaB PE=3 SV=2 - [GSAB_BACSU]	2.33	67.70	8.86	5	3	158.03	11.19	7	3
**O34579**	Uncharacterized protein yfkD OS=Bacillus subtilis GN=yfkD PE=4 SV=1 - [YFKD_BACSU]	1.85	85.32	13.26	4	2	157.63	18.94	6	4
**P40806**	Polyketide synthase pksJ OS=Bacillus subtilis GN=pksJ PE=1 SV=3 - [PKSJ_BACSU]	3.39	46.27	0.52	3	3	156.91	1.69	10	8
**P39601**	Uncharacterized HTH-type transcriptional regulator ywcC OS=Bacillus subtilis GN=ywcC PE=4 SV=2 - [YWCC_BACSU]	1.88	83.27	19.28	6	4	156.33	25.56	7	5
**P37468**	Ribosomal RNA small subunit methyltransferase A OS=Bacillus subtilis GN=rsmA PE=3 SV=1 - [RSMA_BACSU]	3.56	43.48	13.70	4	3	154.84	14.04	6	4
**P39593**	Hydroxyethylthiazole kinase OS=Bacillus subtilis GN=thiM PE=1 SV=1 - [THIM_BACSU]	1.09	141.56	22.43	6	4	154.61	13.24	5	3
**O34885**	Type-2 restriction enzyme BsuMI component ydiS OS=Bacillus subtilis GN=ydiS PE=2 SV=1 - [YDIS_BACSU]	1.49	103.51	12.54	7	4	154.52	13.41	8	4
**P35165**	RNA polymerase sigma factor sigX OS=Bacillus subtilis GN=sigX PE=1 SV=2 - [SIGX_BACSU]	#DIV/0!					154.15	40.72	7	6
**O34628**	Uncharacterized protein yvlB OS=Bacillus subtilis GN=yvlB PE=4 SV=1 - [YVLB_BACSU]	0.90	167.92	13.70	9	5	151.47	21.92	9	7
**O32044**	Single-stranded-DNA-specific exonuclease recJ OS=Bacillus subtilis GN=recJ PE=3 SV=1 - [RECJ_BACSU]	3.65	41.41	6.49	5	4	150.94	9.54	5	5
**P04969**	30S ribosomal protein S11 OS=Bacillus subtilis GN=rpsK PE=3 SV=1 - [RS11_BACSU]	0.68	219.00	29.77	9	4	149.87	19.85	6	3
**O07573**	HTH-type transcriptional regulator nsrR OS=Bacillus subtilis GN=nsrR PE=4 SV=1 - [NSRR_BACSU]	1.09	136.41	31.51	4	4	148.88	39.73	5	5
**O54408**	GTP pyrophosphokinase OS=Bacillus subtilis GN=relA PE=3 SV=3 - [RELA_BACSU]	2.00	74.35	5.59	8	3	148.50	4.77	7	3
**P26901**	Vegetative catalase OS=Bacillus subtilis GN=katA PE=1 SV=5 - [CATA_BACSU]	0.33	454.81	31.68	23	13	148.04	21.33	10	8
**P37599**	Chemotaxis protein cheV OS=Bacillus subtilis GN=cheV PE=2 SV=1 - [CHEV_BACSU]	0.79	188.02	23.43	9	5	147.70	21.78	6	4
**P23446**	Flagellar basal-body rod protein flgG OS=Bacillus subtilis GN=flgG PE=3 SV=2 - [FLGG_BACSU]	0.64	229.79	38.26	9	6	147.52	32.95	6	6
**P21475**	30S ribosomal protein S18 OS=Bacillus subtilis GN=rpsR PE=1 SV=3 - [RS18_BACSU]	1.13	129.72	36.71	11	4	147.17	36.71	8	4
**P39772**	Asparaginyl-tRNA synthetase OS=Bacillus subtilis GN=asnS PE=3 SV=2 - [SYN_BACSU]	0.43	336.67	26.51	22	10	145.30	19.53	8	7
**P24136**	Oligopeptide transport ATP-binding protein oppD OS=Bacillus subtilis GN=oppD PE=2 SV=2 - [OPPD_BACSU]	3.34	43.48	0.00	3	1	145.08	10.89	5	3
**O34921**	Uncharacterized protein ytoI OS=Bacillus subtilis GN=ytoI PE=4 SV=1 - [YTOI_BACSU]	0.48	301.16	20.73	17	7	144.73	9.34	6	3
**P35163**	Transcriptional regulatory protein resD OS=Bacillus subtilis GN=resD PE=3 SV=2 - [RESD_BACSU]	0.92	158.14	25.42	6	5	144.70	31.67	9	6
**O32000**	SPBc2 prophage-derived pesticidal crystal protein-like yokG OS=Bacillus subtilis GN=yokG PE=3 SV=1 - [YOKG_BACSU]	1.27	112.82	12.04	5	3	142.99	20.17	7	5
**P12875**	50S ribosomal protein L14 OS=Bacillus subtilis GN=rplN PE=3 SV=1 - [RL14_BACSU]	1.00	142.98	18.85	6	3	142.31	18.85	7	3
**O32002**	SPBc2 prophage-derived uncharacterized protein yokE OS=Bacillus subtilis GN=yokE PE=4 SV=1 - [YOKE_BACSU]	1.10	129.52	40.00	8	6	141.87	39.38	9	6
**P32728**	Uncharacterized protein ylxR OS=Bacillus subtilis GN=ylxR PE=4 SV=1 - [YLXR_BACSU]	1.80	78.89	42.86	4	4	141.85	42.86	7	5
**P54470**	Putative phosphotransferase yqfL OS=Bacillus subtilis GN=yqfL PE=1 SV=1 - [YQFL_BACSU]	0.51	279.74	27.78	10	7	141.54	27.41	8	7
**O34338**	Manganese transport system ATP-binding protein mntB OS=Bacillus subtilis GN=mntB PE=3 SV=1 - [MNTB_BACSU]	0.96	147.09	23.60	6	5	141.11	29.60	7	6
**P39779**	GTP-sensing transcriptional pleiotropic repressor codY OS=Bacillus subtilis GN=codY PE=1 SV=3 - [CODY_BACSU]	0.81	170.99	23.17	8	6	139.25	17.37	5	5

**P42975**	Bifunctional protein birA OS=Bacillus subtilis GN=birA PE=1 SV=1 - [BIRA_BACSU]	0.85	162.39	13.23	5	3	137.93	13.85	8	3
**P35136**	D-3-phosphoglycerate dehydrogenase OS=Bacillus subtilis GN=serA PE=3 SV=3 - [SERA_BACSU]	1.72	79.81	10.86	5	4	136.99	10.86	4	4
**P23545**	Alkaline phosphatase synthesis sensor protein phoR OS=Bacillus subtilis GN=phoR PE=1 SV=1 - [PHOR_BACSU]	5.08	26.95	0.00	1	1	136.99	8.64	5	4
**Q08352**	Alanine dehydrogenase OS=Bacillus subtilis GN=ald PE=3 SV=1 - [DHA_BACSU]	0.14	994.88	50.00	54	17	136.85	28.04	10	7
**P12874**	30S ribosomal protein S17 OS=Bacillus subtilis GN=rpsQ PE=1 SV=3 - [RS17_BACSU]	0.68	199.62	25.29	18	3	136.59	29.89	11	5
**P20277**	50S ribosomal protein L17 OS=Bacillus subtilis GN=rplQ PE=3 SV=2 - [RL17_BACSU]	0.63	217.26	32.50	8	3	135.80	32.50	6	3
**P37524**	Nucleoid occlusion protein OS=Bacillus subtilis GN=noc PE=1 SV=1 - [NOC_BACSU]	0.69	197.47	16.61	9	4	135.65	15.19	9	4
**P46908**	Anaerobic regulatory protein OS=Bacillus subtilis GN=fnr PE=2 SV=1 - [FNR_BACSU]	0.93	144.47	17.23	4	4	134.08	13.45	3	3
**P36945**	Ribokinase OS=Bacillus subtilis GN=rbsK PE=3 SV=2 - [RBSK_BACSU]	0.58	228.65	20.48	7	4	133.71	14.33	6	3
**O34450**	N-acetylglucosamine-6-phosphate deacetylase OS=Bacillus subtilis GN=nagA PE=1 SV=1 - [NAGA_BACSU]	1.10	120.58	18.18	9	6	133.19	23.74	9	6
**P39070**	ATP-dependent protease hslV OS=Bacillus subtilis GN=hslV PE=1 SV=1 - [HSLV_BACSU]	0.91	145.09	22.65	4	3	132.75	17.13	3	2
**P50849**	Polyribonucleotide nucleotidyltransferase OS=Bacillus subtilis GN=pnp PE=1 SV=3 - [PNP_BACSU]	0.73	179.72	14.89	10	9	131.86	3.55	4	2
**O07592**	Putative glycerophosphoryl diester phosphodiesterase yhdW OS=Bacillus subtilis GN=yhdW PE=3 SV=1 - [YHDW_BACSU]	1.16	113.47	23.46	6	5	131.70	17.70	5	4
**P49849**	DNA mismatch repair protein mutS OS=Bacillus subtilis GN=mutS PE=3 SV=3 - [MUTS_BACSU]	4.48	29.37	5.83	5	4	131.52	6.99	7	5
**P13484**	Probable poly(glycerol-phosphate) alpha-glucosyltransferase OS=Bacillus subtilis GN=tagE PE=1 SV=1 - [TAGE_BACSU]	0.80	163.85	9.81	6	5	130.37	7.88	8	4
**P50840**	Putative RNA methyltransferase ypsC OS=Bacillus subtilis GN=ypsC PE=3 SV=1 - [YPSC_BACSU]	#DIV/0!					129.75	12.21	5	4
**P30950**	Delta-aminolevulinic acid dehydratase OS=Bacillus subtilis GN=hemB PE=3 SV=1 - [HEM2_BACSU]	0.75	171.92	20.99	13	6	128.96	16.05	9	5
**P39845**	Plipastatin synthase subunit A OS=Bacillus subtilis GN=ppsA PE=1 SV=2 - [PPSA_BACSU]	1.88	68.08	0.00	7	4	128.17	2.58	7	5
**P40762**	Uncharacterized HTH-type transcriptional regulator yvmB OS=Bacillus subtilis GN=yvmB PE=4 SV=1 - [YVMB_BACSU]	2.30	55.64	18.34	3	3	128.17	31.36	5	5
**P46914**	Spore coat protein S OS=Bacillus subtilis GN=cotS PE=1 SV=1 - [COTS_BACSU]	0.92	138.50	19.66	12	6	128.03	17.09	14	5
**O32158**	Uncharacterized protein yurQ OS=Bacillus subtilis GN=yurQ PE=4 SV=1 - [YURQ_BACSU]	1.93	66.26	25.81	4	3	128.02	36.29	5	4
**P55910**	L-lactate permease OS=Bacillus subtilis GN=lctP PE=2 SV=3 - [LCTP_BACSU]	1.72	73.31	9.43	7	4	126.12	7.58	10	3
**Q45585**	RNA polymerase sigma factor sigW OS=Bacillus subtilis GN=sigW PE=2 SV=1 - [SIGW_BACSU]	#DIV/0!					126.04	15.51	6	4
**O34592**	AB hydrolase superfamily protein ydjP OS=Bacillus subtilis GN=ydjP PE=2 SV=1 - [YDJP_BACSU]	0.93	133.89	16.97	5	4	124.80	12.92	4	3
**O07921**	Chitosanase OS=Bacillus subtilis GN=csn PE=1 SV=1 - [CHIS_BACSU]	0.84	146.25	23.47	9	6	122.70	23.47	8	5
**P06533**	HTH-type transcriptional regulator sinR OS=Bacillus subtilis GN=sinR PE=1 SV=1 - [SINR_BACSU]	2.72	44.75	20.72	2	2	121.75	22.52	3	3
**P39796**	Trehalose operon transcriptional repressor OS=Bacillus subtilis GN=treR PE=1 SV=2 - [TRER_BACSU]	0.52	234.85	33.61	13	7	121.08	32.77	10	7
**P16336**	Preprotein translocase subunit secY OS=Bacillus subtilis GN=secY PE=3 SV=1 - [SECY_BACSU]	0.84	143.77	9.05	13	3	120.89	6.26	6	2

**P80861**	NADH dehydrogenase-like protein yjlD OS=Bacillus subtilis GN=yjlD PE=1 SV=3 - [YJLD_BACSU]	0.65	186.77	13.27	7	4	120.75	6.63	5	2
**P39216**	Methyl-accepting chemotaxis protein tlpA OS=Bacillus subtilis GN=tlpA PE=3 SV=1 - [TLPA_BACSU]	0.48	249.59	9.21	15	6	120.20	0.00	5	3
**P29141**	Minor extracellular protease vpr OS=Bacillus subtilis GN=vpr PE=1 SV=1 - [SUBV_BACSU]	1.02	117.37	10.30	6	5	119.34	8.06	4	4
**P96650**	Uncharacterized protein yddM OS=Bacillus subtilis GN=yddM PE=4 SV=1 - [YDDM_BACSU]	1.01	117.93	21.73	10	5	118.86	14.38	7	3
**O31632**	Cystathionine beta-lyase metC OS=Bacillus subtilis GN=metC PE=1 SV=1 - [METC_BACSU]	2.05	57.41	8.46	3	2	117.75	12.56	5	3
**P39646**	Phosphate acetyltransferase OS=Bacillus subtilis GN=pta PE=1 SV=3 - [PTA_BACSU]	0.75	156.89	17.96	12	5	117.16	15.48	5	4
**P46912**	Menaquinol-cytochrome c reductase cytochrome b subunit OS=Bacillus subtilis GN=qcrB PE=1 SV=1 - [QCRB_BACSU]	2.10	54.61	15.18	5	2	114.74	22.32	5	4
**P33189**	Uncharacterized aminotransferase yhxA OS=Bacillus subtilis GN=yhxA PE=3 SV=4 - [YHXA_BACSU]	0.39	293.88	24.44	14	8	114.32	11.11	5	4
**P28368**	Uncharacterized protein yvyD OS=Bacillus subtilis GN=yvyD PE=3 SV=2 - [YVYD_BACSU]	0.49	235.53	26.46	11	5	114.31	25.93	7	5
**P21883**	Dihydrolipoyllysine-residue acetyltransferase component of pyruvate dehydrogenase complex OS=Bacillus subtilis GN=pdhC PE=1 SV=2 - [ODP2_BACSU]	0.19	598.05	40.50	35	15	113.90	13.80	11	4
**P71009**	Putative ABC transporter ATP-binding protein albC OS=Bacillus subtilis GN=albC PE=2 SV=1 - [ALBC_BACSU]	1.02	111.74	20.92	4	4	113.74	18.41	5	4
**Q45598**	Uncharacterized protein yydD OS=Bacillus subtilis GN=yydD PE=4 SV=1 - [YYDD_BACSU]	0.43	264.11	6.14	10	3	113.68	8.70	5	4
**P94387**	Uncharacterized HTH-type transcriptional regulator ycgK OS=Bacillus subtilis GN=ycgK PE=4 SV=2 - [YCGK_BACSU]	1.80	63.07	11.73	4	3	113.28	26.23	9	6
**O34676**	L-lysine 2,3-aminomutase OS=Bacillus subtilis GN=kamA PE=1 SV=1 - [KAMA_BACSU]	1.90	59.47	5.31	3	2	113.01	7.86	4	3
**O34607**	Probable L-serine dehydratase, alpha chain OS=Bacillus subtilis GN=sdaAA PE=3 SV=1 - [SDHA_BACSU]	1.37	81.12	10.67	3	2	111.47	15.00	4	3
**P40780**	Uncharacterized protein ytxH OS=Bacillus subtilis GN=ytxH PE=4 SV=2 - [YTXH_BACSU]	0.30	365.31	50.99	13	7	110.77	27.81	4	4
**P55180**	UDP-glucose 4-epimerase OS=Bacillus subtilis GN=galE PE=3 SV=1 - [GALE_BACSU]	0.81	135.65	15.04	8	4	110.39	18.58	8	5
**O06477**	Putative sensory transducer protein yfmS OS=Bacillus subtilis GN=yfmS PE=2 SV=1 - [YFMS_BACSU]	0.52	212.28	16.78	14	5	110.31	15.03	6	4
**O34514**	o-succinylbenzoate synthase OS=Bacillus subtilis GN=menC PE=1 SV=1 - [MENC_BACSU]	0.71	155.08	21.56	9	7	110.22	11.86	4	4
**P54331**	Phage-like element PBSX protein xkdK OS=Bacillus subtilis GN=xkdK PE=4 SV=2 - [XKDK_BACSU]	#DIV/0!					110.15	12.02	5	4
**O06973**	UPF0042 nucleotide-binding protein yvcJ OS=Bacillus subtilis GN=yvcJ PE=1 SV=1 - [YVCJ_BACSU]	0.98	111.92	10.51	5	3	109.71	12.54	6	3
**P39456**	L-cystine import ATP-binding protein tcyC OS=Bacillus subtilis GN=tcyC PE=1 SV=4 - [TCYC_BACSU]	1.70	64.21	10.93	2	2	109.47	10.93	3	2
**P54531**	Leucine dehydrogenase OS=Bacillus subtilis GN=yqiT PE=3 SV=1 - [DHLE_BACSU]	0.21	529.96	28.30	22	9	108.83	25.00	12	7
**P96605**	Uncharacterized ABC transporter ATP-binding protein ydbJ OS=Bacillus subtilis GN=ydbJ PE=3 SV=1 - [YDBJ_BACSU]	0.68	160.53	26.95	9	6	108.79	21.43	7	5
**O31740**	Ribosome maturation factor rimM OS=Bacillus subtilis GN=rimM PE=3 SV=1 - [RIMM_BACSU]	1.46	74.44	28.16	6	4	108.38	28.74	7	5
**O30509**	Aspartyl/glutamyl-tRNA(Asn/Gln) amidotransferase subunit B OS=Bacillus subtilis GN=gatB PE=1 SV=2 - [GATB_BACSU]	0.23	456.54	26.26	22	10	107.16	7.35	5	3
**Q04797**	Aspartate-semialdehyde dehydrogenase OS=Bacillus subtilis GN=asd PE=1 SV=1 - [DHAS_BACSU]	0.30	353.35	28.03	12	8	106.87	0.00	4	1
**P39788**	Probable endonuclease III OS=Bacillus subtilis GN=nth PE=3 SV=1 - [END3_BACSU]	0.82	129.87	22.83	6	5	106.39	18.26	6	4

**P37105**	Signal recognition particle protein OS=Bacillus subtilis GN=ffh PE=1 SV=1 - [SRP54_BACSU]	0.34	313.45	15.02	14	5	105.86	12.56	7	4
**O35009**	Probable integrase/recombinase yoeC OS=Bacillus subtilis GN=yoeC PE=3 SV=2 - [YOEC_BACSU]	3.38	31.26	15.47	2	2	105.59	32.04	5	5
**P27876**	Triosephosphate isomerase OS=Bacillus subtilis GN=tpiA PE=1 SV=3 - [TPIS_BACSU]	0.25	419.33	33.60	15	11	105.56	20.16	7	5
**P23479**	Nuclease sbcCD subunit D OS=Bacillus subtilis GN=sbcD PE=3 SV=5 - [SBCD_BACSU]	0.56	188.64	13.30	12	4	105.29	10.23	4	3
**P32727**	Transcription elongation protein nusA OS=Bacillus subtilis GN=nusA PE=3 SV=2 - [NUSA_BACSU]	0.44	235.96	18.60	13	7	104.94	9.70	5	3
**O34448**	SPBc2 prophage-derived uncharacterized protein yopQ OS=Bacillus subtilis GN=yopQ PE=4 SV=1 - [YOPQ_BACSU]	0.64	161.41	14.57	10	6	104.04	5.43	5	2
**P39149**	Uracil phosphoribosyltransferase OS=Bacillus subtilis GN=upp PE=3 SV=1 - [UPP_BACSU]	0.73	140.92	28.23	8	4	103.43	20.10	6	4
**P50839**	Cell cycle protein gpsB OS=Bacillus subtilis GN=gpsB PE=1 SV=1 - [GPSB_BACSU]	1.06	97.18	15.31	4	2	103.05	16.33	5	3
**P39848**	Beta-N-acetylglucosaminidase OS=Bacillus subtilis GN=lytD PE=1 SV=1 - [LYTD_BACSU]	1.81	56.81	7.39	7	5	103.01	8.30	7	5
**Q45498**	UPF0637 protein yktB OS=Bacillus subtilis GN=yktB PE=1 SV=1 - [YKTB_BACSU]	1.01	100.84	14.15	4	2	102.30	19.34	3	3
**P80239**	Alkyl hydroperoxide reductase subunit C OS=Bacillus subtilis GN=ahpC PE=1 SV=2 - [AHPC_BACSU]	0.49	209.46	34.76	7	5	101.91	15.51	3	2
**P54616**	Enoyl-[acyl-carrier-protein] reductase [NADH] OS=Bacillus subtilis GN=fabI PE=1 SV=2 - [FABI_BACSU]	0.70	146.30	27.52	9	7	101.83	19.77	7	6
**P51785**	Dihydroxy-acid dehydratase OS=Bacillus subtilis GN=ilvD PE=1 SV=4 - [ILVD_BACSU]	1.15	88.71	8.60	10	5	101.77	12.37	6	5
**O07608**	Putative lipoate-protein ligase A OS=Bacillus subtilis GN=lplA PE=3 SV=1 - [LPLA_BACSU]	0.47	218.52	22.66	10	6	101.62	16.01	6	4
**P96618**	Holo-[acyl-carrier-protein] synthase OS=Bacillus subtilis GN=acpS PE=1 SV=1 - [ACPS_BACSU]	1.01	100.36	18.18	2	2	101.49	18.18	2	2
**P70974**	50S ribosomal protein L13 OS=Bacillus subtilis GN=rplM PE=1 SV=2 - [RL13_BACSU]	0.56	181.63	34.48	11	6	101.44	28.28	10	4
**O06967**	Multidrug resistance ABC transporter ATP-binding/permease protein bmrA OS=Bacillus subtilis GN=bmrA PE=1 SV=1 - [BMRA_BACSU]	0.94	107.76	10.02	11	5	100.98	8.66	5	4
**O32176**	Probable acyl-CoA dehydrogenase OS=Bacillus subtilis GN=fadE PE=2 SV=1 - [FADE_BACSU]	0.62	161.56	10.27	11	5	100.65	8.25	4	3
**P21476**	30S ribosomal protein S19 OS=Bacillus subtilis GN=rpsS PE=1 SV=3 - [RS19_BACSU]	1.42	70.71	28.26	4	3	100.50	28.26	5	3
**P10726**	RNA polymerase sigma-D factor OS=Bacillus subtilis GN=sigD PE=1 SV=2 - [RPSD_BACSU]	#DIV/0!					100.02	10.63	3	2
**P54484**	Cell wall-binding protein yqgA OS=Bacillus subtilis GN=yqgA PE=1 SV=2 - [YQGA_BACSU]	1.13	88.44	24.65	3	3	99.87	16.90	3	2
**P24010**	Cytochrome c oxidase subunit 1 OS=Bacillus subtilis GN=ctaD PE=3 SV=3 - [COX1_BACSU]	1.53	64.52	5.31	6	3	98.93	6.11	4	3
**P49787**	Biotin carboxylase 1 OS=Bacillus subtilis GN=accC1 PE=3 SV=3 - [ACCC1_BACSU]	0.73	135.03	11.78	8	4	98.83	12.44	5	4
**O31582**	Uncharacterized protein yfhO OS=Bacillus subtilis GN=yfhO PE=4 SV=2 - [YFHO_BACSU]	0.46	212.00	13.94	15	11	97.53	6.85	8	6
**O31571**	Putative NAD(P)H nitroreductase yfhC OS=Bacillus subtilis GN=yfhC PE=3 SV=1 - [YFHC_BACSU]	1.30	74.75	27.32	5	5	97.46	12.89	5	2
**P39763**	Protein mreBH OS=Bacillus subtilis GN=mreBH PE=2 SV=1 - [MREBH_BACSU]	0.85	114.45	33.43	9	7	96.92	24.78	8	6
**Q05522**	Chemotaxis response regulator protein-glutamate methylesterase OS=Bacillus subtilis GN=cheB PE=3 SV=2 - [CHEB_BACSU]	0.38	253.81	18.49	13	6	96.67	15.41	5	4
**P18579**	UDP-N-acetylenolpyruvoylglucosamine reductase OS=Bacillus subtilis GN=murB PE=3 SV=1 - [MURB_BACSU]	0.75	128.20	16.17	6	4	96.66	10.89	4	3

**P37494**	Uncharacterized ABC transporter ATP-binding protein yybJ OS=Bacillus subtilis GN=yybJ PE=3 SV=1 - [YYBJ_BACSU]	1.01	95.62	22.94	4	4	96.62	20.64	4	4
**O34841**	Uncharacterized protein yoeB OS=Bacillus subtilis GN=yoeB PE=2 SV=2 - [YOEB_BACSU]	0.76	127.60	32.60	8	5	96.37	24.86	8	4
**P39765**	Bifunctional protein pyrR OS=Bacillus subtilis GN=pyrR PE=1 SV=2 - [PYRR_BACSU]	0.66	146.45	23.20	4	4	96.34	24.86	5	4
**P12879**	30S ribosomal protein S8 OS=Bacillus subtilis GN=rpsH PE=1 SV=4 - [RS8_BACSU]	0.74	129.02	27.27	6	4	95.84	27.27	5	3
**P36948**	Ribose transport system permease protein rbsC OS=Bacillus subtilis GN=rbsC PE=3 SV=2 - [RBSC_BACSU]	0.64	148.13	12.11	8	4	95.07	9.63	8	4
**P25814**	Ribonuclease P protein component OS=Bacillus subtilis GN=rnpA PE=1 SV=2 - [RNPA_BACSU]	0.75	126.76	40.52	8	5	94.97	40.52	5	5
**P46911**	Menaquinol-cytochrome c reductase iron-sulfur subunit OS=Bacillus subtilis GN=qcrA PE=3 SV=1 - [QCRA_BACSU]	0.64	147.57	26.95	5	4	94.96	16.17	2	2
**O34381**	HTH-type transcriptional regulator pksA OS=Bacillus subtilis GN=pksA PE=4 SV=1 - [PKSA_BACSU]	1.50	63.13	12.68	3	2	94.88	20.00	7	3
**P39754**	Glucosamine--fructose-6-phosphate aminotransferase [isomerizing] OS=Bacillus subtilis GN=glmS PE=3 SV=3 - [GLMS_BACSU]	0.34	281.17	15.50	23	9	94.57	9.83	8	6
**P71088**	Sporulation-control protein spo0M OS=Bacillus subtilis GN=spo0M PE=1 SV=2 - [SP0M_BACSU]	0.72	130.69	13.18	5	3	94.38	8.91	3	2
**P25499**	Heat-inducible transcription repressor hrcA OS=Bacillus subtilis GN=hrcA PE=3 SV=1 - [HRCA_BACSU]	0.94	99.94	13.99	7	4	93.93	13.41	4	4
**P39062**	Acetyl-coenzyme A synthetase OS=Bacillus subtilis GN=acsA PE=3 SV=1 - [ACSA_BACSU]	2.54	36.34	3.67	4	2	92.14	6.47	4	3
**P24139**	Oligopeptide transport system permease protein oppC OS=Bacillus subtilis GN=oppC PE=2 SV=1 - [OPPC_BACSU]	0.61	149.36	15.08	6	3	91.49	9.51	4	2
**O34948**	Uncharacterized oxidoreductase ykwC OS=Bacillus subtilis GN=ykwC PE=3 SV=1 - [YKWC_BACSU]	1.32	69.16	10.07	2	2	91.23	0.00	2	1
**O05268**	Ferredoxin--NADP reductase 2 OS=Bacillus subtilis GN=yumC PE=1 SV=2 - [FENR2_BACSU]	0.21	418.85	27.71	17	8	89.86	13.25	3	3
**P08066**	Succinate dehydrogenase iron-sulfur subunit OS=Bacillus subtilis GN=sdhB PE=3 SV=3 - [DHSB_BACSU]	0.69	130.12	20.55	8	4	89.59	13.44	6	4
**P06534**	Stage 0 sporulation protein A OS=Bacillus subtilis GN=spo0A PE=1 SV=1 - [SP0A_BACSU]	0.61	145.82	13.86	5	3	89.50	9.36	5	2
**P19669**	Transaldolase OS=Bacillus subtilis GN=tal PE=1 SV=4 - [TAL_BACSU]	0.58	153.07	38.68	11	8	89.44	27.83	8	5
**P42956**	PTS system mannitol-specific EIICB component OS=Bacillus subtilis GN=mtlA PE=3 SV=4 - [PTMCB_BACSU]	0.24	372.48	8.16	15	3	89.12	8.16	4	3
**O07521**	3'-5' exoribonuclease yhaM OS=Bacillus subtilis GN=yhaM PE=2 SV=1 - [YHAM_BACSU]	1.37	64.51	8.92	4	2	88.69	16.56	8	4
**P54552**	Pyrroline-5-carboxylate reductase 2 OS=Bacillus subtilis GN=proI PE=3 SV=1 - [P5CR2_BACSU]	0.95	91.48	26.26	6	6	86.77	20.86	6	4
**P94559**	Putative metallophosphoesterase ysnB OS=Bacillus subtilis GN=ysnB PE=3 SV=2 - [YSNB_BACSU]	0.59	146.14	26.04	5	4	85.82	20.71	3	3
**P80244**	ATP-dependent Clp protease proteolytic subunit OS=Bacillus subtilis GN=clpP PE=1 SV=3 - [CLPP_BACSU]	0.77	110.36	19.80	8	5	85.39	19.80	4	3
**C0H3R3**	Uncharacterized protein yvzF OS=Bacillus subtilis GN=yvzF PE=4 SV=1 - [YVZF_BACSU]	2.07	41.17	0.00	1	1	85.23	21.31	4	2
**O34617**	Ribosomal RNA large subunit methyltransferase N OS=Bacillus subtilis GN=rlmN PE=3 SV=1 - [RLMN_BACSU]	0.81	104.52	10.19	4	3	85.11	9.92	4	3
**P12464**	DNA-directed RNA polymerase subunit delta OS=Bacillus subtilis GN=rpoE PE=1 SV=1 - [RPOE_BACSU]	#DIV/0!					85.09	24.28	3	3
**O31847**	Uncharacterized protein yozO OS=Bacillus subtilis GN=yozO PE=4 SV=1 - [YOZO_BACSU]	2.23	37.78	0.00	1	1	84.18	20.18	2	2
**P46920**	Glycine betaine transport ATP-binding protein opuAA OS=Bacillus subtilis GN=opuAA PE=3 SV=3 - [OPUAA_BACSU]	0.20	409.91	11.72	17	6	83.65	9.33	3	3

**P37946**	Threonine dehydratase biosynthetic OS=Bacillus subtilis GN=ilvA PE=3 SV=1 - [THD1_BACSU]	0.19	436.18	25.83	18	9	83.64	7.58	3	2
**P37811**	ATP synthase subunit delta OS=Bacillus subtilis GN=atpH PE=3 SV=1 - [ATPD_BACSU]	1.66	50.41	7.73	2	2	83.53	13.26	3	3
**P49938**	Iron(3+)-hydroxamate import ATP-binding protein fhuC OS=Bacillus subtilis GN=fhuC PE=3 SV=1 - [FHUC_BACSU]	1.01	81.68	14.50	3	3	82.79	10.04	2	2
**P54421**	Probable endopeptidase lytE OS=Bacillus subtilis GN=lytE PE=1 SV=1 - [LYTE_BACSU]	3.69	22.33	0.00	1	1	82.40	9.58	4	3
**P54476**	Probable endonuclease 4 OS=Bacillus subtilis GN=nfo PE=3 SV=1 - [END4_BACSU]	2.20	37.30	0.00	1	1	82.01	10.44	5	3
**P55342**	UPF0747 protein yllA OS=Bacillus subtilis GN=yllA PE=3 SV=3 - [YLLA_BACSU]	0.61	131.87	8.16	8	4	81.08	10.39	6	5
**P42976**	Dihydrodipicolinate reductase OS=Bacillus subtilis GN=dapB PE=3 SV=2 - [DAPB_BACSU]	0.63	128.72	6.74	6	3	80.88	16.10	7	3
**Q04795**	Aspartokinase 1 OS=Bacillus subtilis GN=dapG PE=3 SV=3 - [AK1_BACSU]	0.35	225.55	20.05	10	6	80.04	18.07	8	5
**P25503**	Urocanate hydratase OS=Bacillus subtilis GN=hutU PE=1 SV=2 - [HUTU_BACSU]	0.56	142.28	11.05	8	5	79.73	6.52	3	3
**O31681**	Spore protein ykvP OS=Bacillus subtilis GN=ykvP PE=2 SV=1 - [YKVP_BACSU]	0.83	95.72	0.00	3	1	79.57	10.78	4	3
**O07587**	Putative aspartate aminotransferase yhdR OS=Bacillus subtilis GN=yhdR PE=3 SV=1 - [AAT3_BACSU]	0.64	122.70	16.79	9	5	78.92	16.79	10	5
**P94583**	Response regulator aspartate phosphatase D OS=Bacillus subtilis GN=rapD PE=3 SV=1 - [RAPD_BACSU]	2.44	32.27	8.76	4	3	78.82	6.21	3	2
**O32102**	Ferri-bacillibactin esterase besA OS=Bacillus subtilis GN=besA PE=1 SV=2 - [BESA_BACSU]	#DIV/0!					78.70	7.61	3	2
**P13792**	Alkaline phosphatase synthesis transcriptional regulatory protein phoP OS=Bacillus subtilis GN=phoP PE=1 SV=4 - [PHOP_BACSU]	0.50	154.59	17.92	5	4	77.50	10.83	4	2
**O31498**	DNA ligase OS=Bacillus subtilis GN=ligA PE=3 SV=1 - [DNLJ_BACSU]	2.48	31.20	3.74	3	2	77.41	9.43	5	5
**P37474**	Transcription-repair-coupling factor OS=Bacillus subtilis GN=mfd PE=3 SV=1 - [MFD_BACSU]	0.65	118.67	4.08	6	4	77.41	3.31	5	3
**P96612**	D-alanine--D-alanine ligase OS=Bacillus subtilis GN=ddl PE=3 SV=1 - [DDL_BACSU]	0.48	160.27	14.41	7	4	76.89	7.06	3	2
**P37963**	Stage VI sporulation protein D OS=Bacillus subtilis GN=spoVID PE=2 SV=1 - [SP6D_BACSU]	1.12	68.56	14.96	7	7	76.86	6.43	7	3
**P28612**	Chemotaxis protein motB OS=Bacillus subtilis GN=motB PE=3 SV=1 - [MOTB_BACSU]	0.45	168.69	22.61	8	5	76.58	19.54	5	4
**P51834**	Chromosome partition protein smc OS=Bacillus subtilis GN=smc PE=1 SV=3 - [SMC_BACSU]	0.88	86.64	7.08	8	7	76.23	4.55	5	4
**O34454**	UPF0111 protein ykaA OS=Bacillus subtilis GN=ykaA PE=3 SV=1 - [YKAA_BACSU]	1.05	72.17	11.22	4	2	76.09	11.22	3	2
**O31581**	AB hydrolase superfamily protein yfhM OS=Bacillus subtilis GN=yfhM PE=3 SV=1 - [YFHM_BACSU]	0.43	176.09	24.13	9	5	75.95	14.69	4	3
**O34478**	Uncharacterized protein yccF OS=Bacillus subtilis GN=yccF PE=4 SV=1 - [YCCF_BACSU]	0.66	114.74	10.06	6	3	75.78	6.98	2	2
**P54431**	Uncharacterized protein yrkD OS=Bacillus subtilis GN=yrkD PE=4 SV=1 - [YRKD_BACSU]	0.75	100.63	61.90	4	3	75.52	39.68	2	2
**Q795R8**	Uncharacterized protein ytfP OS=Bacillus subtilis GN=ytfP PE=4 SV=2 - [YTFP_BACSU]	1.91	39.37	8.57	3	3	75.20	5.24	2	2
**P26908**	50S ribosomal protein L21 OS=Bacillus subtilis GN=rplU PE=3 SV=1 - [RL21_BACSU]	0.52	145.21	24.51	7	2	75.07	24.51	3	2
**P54553**	Uncharacterized protein yqjP OS=Bacillus subtilis GN=yqjP PE=4 SV=1 - [YQJP_BACSU]	0.83	90.90	8.46	5	2	75.01	7.84	3	2
**P36430**	Leucyl-tRNA synthetase OS=Bacillus subtilis GN=leuS PE=3 SV=3 - [SYL_BACSU]	0.26	279.53	16.04	14	10	74.07	3.61	3	2

**O31504**	Putative DNA methyltransferase yeeA OS=Bacillus subtilis GN=yeeA PE=4 SV=1 - [YEEA_BACSU]	#DIV/0!					73.88	3.98	6	4
**P54530**	Probable phosphate butyryltransferase OS=Bacillus subtilis GN=yqiS PE=3 SV=2 - [PTB_BACSU]	0.67	109.68	9.03	3	2	73.84	18.06	3	3
**P96729**	Cell wall-binding protein ywsB OS=Bacillus subtilis GN=ywsB PE=1 SV=1 - [YWSB_BACSU]	0.70	104.88	18.54	4	3	73.77	18.54	4	3
**O34874**	Putative cysteine desulfurase iscS 2 OS=Bacillus subtilis GN=iscS2 PE=3 SV=1 - [ISCS2_BACSU]	1.09	67.73	0.00	1	1	73.54	10.24	4	3
**O31654**	RNA polymerase sigma factor sigI OS=Bacillus subtilis GN=sigI PE=1 SV=1 - [SIGI_BACSU]	#DIV/0!					73.01	13.55	7	4
**P70947**	Putative phosphatase yitU OS=Bacillus subtilis GN=yitU PE=3 SV=1 - [YITU_BACSU]	0.95	76.80	8.52	2	2	73.00	12.96	4	3
**P07372**	Stage II sporulation protein D OS=Bacillus subtilis GN=spoIID PE=4 SV=1 - [SP2D_BACSU]	#DIV/0!					72.99	9.62	3	3
**O32259**	Lactate utilization protein C OS=Bacillus subtilis GN=lutC PE=1 SV=1 - [LUTC_BACSU]	0.48	151.79	16.25	7	4	72.97	5.83	3	2
**P39574**	Galactokinase OS=Bacillus subtilis GN=galK PE=3 SV=1 - [GAL1_BACSU]	0.96	75.89	11.03	5	4	72.91	5.90	2	2
**P54325**	Phage-like element PBSX protein xkdE OS=Bacillus subtilis GN=xkdE PE=3 SV=2 - [XKDE_BACSU]	0.71	99.75	5.05	5	2	71.19	8.28	3	3
**P24137**	Oligopeptide transport ATP-binding protein oppF OS=Bacillus subtilis GN=oppF PE=2 SV=3 - [OPPF_BACSU]	1.26	56.50	15.74	5	4	71.03	11.48	4	3
**O32037**	Uncharacterized protein yrvM OS=Bacillus subtilis GN=yrvM PE=3 SV=3 - [YRVM_BACSU]	0.74	96.11	15.75	5	3	70.99	16.14	3	3
**P39138**	Arginase OS=Bacillus subtilis GN=rocF PE=2 SV=1 - [ARGI_BACSU]	0.14	483.69	25.68	29	7	69.98	15.88	9	4
**P13267**	DNA polymerase III polC-type OS=Bacillus subtilis GN=polC PE=1 SV=2 - [DPO3_BACSU]	0.93	74.65	2.51	5	3	69.33	4.59	5	5
**P71047**	Putative HTH-type transcriptional regulator ywgB OS=Bacillus subtilis GN=ywgB PE=4 SV=1 - [YWGB_BACSU]	0.91	75.80	25.00	3	3	69.01	19.23	2	2
**O32090**	Nicotinate phosphoribosyltransferase OS=Bacillus subtilis GN=pncB PE=3 SV=1 - [PNCB_BACSU]	0.49	140.34	7.14	6	3	68.85	0.00	1	1
**P28366**	Protein translocase subunit secA OS=Bacillus subtilis GN=secA PE=1 SV=1 - [SECA_BACSU]	0.34	201.48	12.01	17	8	68.35	6.30	6	5
**P17867**	Putative DNA recombinase OS=Bacillus subtilis GN=cisA PE=3 SV=2 - [CISA_BACSU]	0.98	69.36	4.80	4	2	68.23	6.40	3	2
**P50831**	Probable ATP-dependent helicase ypvA OS=Bacillus subtilis GN=ypvA PE=3 SV=1 - [YPVA_BACSU]	#DIV/0!					68.15	5.93	4	3
**P39132**	Uncharacterized protein yhbE OS=Bacillus subtilis GN=yhbE PE=4 SV=2 - [YHBE_BACSU]	1.27	52.98	18.14	3	3	67.38	10.97	4	2
**P96628**	Protein sprT-like OS=Bacillus subtilis GN=ydcK PE=3 SV=1 - [SPRTL_BACSU]	1.30	51.51	0.00	1	1	66.80	15.33	2	2
**P37536**	Uncharacterized protein yaaO OS=Bacillus subtilis GN=yaaO PE=3 SV=1 - [YAAO_BACSU]	0.70	95.51	11.46	9	5	66.79	6.88	5	3
**P37965**	Glycerophosphoryl diester phosphodiesterase OS=Bacillus subtilis GN=glpQ PE=3 SV=1 - [GLPQ_BACSU]	1.41	47.02	18.43	4	4	66.16	9.90	3	2
**O34743**	Uncharacterized protein ylbA OS=Bacillus subtilis GN=ylbA PE=4 SV=1 - [YLBA_BACSU]	0.48	136.94	50.00	7	5	66.03	21.67	3	2
**P42923**	50S ribosomal protein L10 OS=Bacillus subtilis GN=rplJ PE=1 SV=4 - [RL10_BACSU]	0.81	81.30	13.25	3	3	65.65	12.65	2	2
**O34812**	Putative NADP-dependent oxidoreductase yfmJ OS=Bacillus subtilis GN=yfmJ PE=2 SV=1 - [YFMJ_BACSU]	0.41	159.39	13.86	7	5	65.60	0.00	2	1
**O34981**	2,3,4,5-tetrahydropyridine-2,6-dicarboxylate N-acetyltransferase OS=Bacillus subtilis GN=dapH PE=1 SV=2 - [DAPH_BACSU]	1.27	51.61	9.75	2	2	65.42	9.75	3	2
**Q7WY78**	Putative transcriptional regulator ywtF OS=Bacillus subtilis GN=ywtF PE=3 SV=2 - [YWTF_BACSU]	1.49	43.74	0.00	2	1	65.29	12.11	3	3

**P94459**	Plipastatin synthase subunit D OS=Bacillus subtilis GN=ppsD PE=1 SV=2 - [PPSD_BACSU]	0.64	101.85	1.97	9	5	65.21	1.78	5	5
**P40871**	2,3-dihydroxybenzoate-AMP ligase OS=Bacillus subtilis GN=dhbE PE=1 SV=2 - [DHBE_BACSU]	0.97	67.09	2.97	5	2	65.08	9.65	3	3
**O34746**	3-oxoacyl-[acyl-carrier-protein] synthase 3 protein 1 OS=Bacillus subtilis GN=fabHA PE=2 SV=1 - [FABH1_BACSU]	2.69	23.93	0.00	1	1	64.34	9.62	4	2
**P94390**	Proline dehydrogenase 2 OS=Bacillus subtilis GN=ycgM PE=3 SV=1 - [PROD2_BACSU]	#DIV/0!					64.23	7.59	3	2
**P35160**	Thiol-disulfide oxidoreductase resA OS=Bacillus subtilis GN=resA PE=1 SV=2 - [RESA_BACSU]	0.44	144.67	19.55	4	3	64.08	0.00	1	1
**O05521**	Redox-sensing transcriptional repressor rex OS=Bacillus subtilis GN=rex PE=1 SV=1 - [REX_BACSU]	0.63	100.86	14.88	4	3	63.86	10.23	2	2
**P45929**	Uncharacterized protein yqbM OS=Bacillus subtilis GN=yqbM PE=4 SV=3 - [YQBM_BACSU]	#DIV/0!					63.59	17.69	2	2
**P54523**	1-deoxy-D-xylulose-5-phosphate synthase OS=Bacillus subtilis GN=dxs PE=3 SV=1 - [DXS_BACSU]	2.50	25.42	3.00	2	2	63.49	5.85	5	3
**P94428**	Succinate-semialdehyde dehydrogenase [NADP+] OS=Bacillus subtilis GN=gabD PE=1 SV=1 - [GABD_BACSU]	0.62	102.11	4.76	3	2	63.39	8.23	4	3
**P13714**	L-lactate dehydrogenase OS=Bacillus subtilis GN=ldh PE=1 SV=3 - [LDH_BACSU]	0.20	323.11	22.50	14	6	63.31	7.50	4	2
**P37465**	Methionyl-tRNA synthetase OS=Bacillus subtilis GN=metG PE=3 SV=1 - [SYM_BACSU]	0.34	187.30	14.16	12	8	62.98	6.93	3	3
**P39813**	Protein smf OS=Bacillus subtilis GN=smf PE=3 SV=2 - [SMF_BACSU]	#DIV/0!					62.69	13.13	3	3
**O34979**	Uncharacterized ABC transporter ATP-binding protein yvrO OS=Bacillus subtilis GN=yvrO PE=3 SV=2 - [YVRO_BACSU]	0.77	81.71	15.72	6	3	62.64	11.79	4	2
**O31657**	Probable protease htpX homolog OS=Bacillus subtilis GN=htpX PE=3 SV=1 - [HTPX_BACSU]	#DIV/0!					62.40	8.72	2	2
**O32055**	Holliday junction ATP-dependent DNA helicase ruvB OS=Bacillus subtilis GN=ruvB PE=1 SV=2 - [RUVB_BACSU]	0.36	175.62	9.28	8	3	62.36	6.89	2	2
**P37437**	50S ribosomal protein L9 OS=Bacillus subtilis GN=rplI PE=3 SV=1 - [RL9_BACSU]	#DIV/0!					62.24	22.15	4	3
**O32033**	Uridine kinase OS=Bacillus subtilis GN=udk PE=3 SV=1 - [URK_BACSU]	0.69	89.68	12.80	3	2	61.45	17.54	4	3
**P49786**	Biotin carboxyl carrier protein of acetyl-CoA carboxylase OS=Bacillus subtilis GN=accB PE=3 SV=2 - [BCCP_BACSU]	0.23	270.29	69.18	17	5	61.11	0.00	1	1
**P36944**	Ribose operon repressor OS=Bacillus subtilis GN=rbsR PE=4 SV=2 - [RBSR_BACSU]	0.72	84.32	9.82	4	3	61.08	8.28	2	2
**P54532**	Probable butyrate kinase OS=Bacillus subtilis GN=buk PE=3 SV=2 - [BUK_BACSU]	0.38	158.58	12.67	7	4	60.99	0.00	3	1
**P16524**	Putative aminotransferase A OS=Bacillus subtilis GN=patA PE=1 SV=3 - [PATA_BACSU]	1.55	39.29	6.62	2	2	60.85	10.94	6	3
**P54159**	Uncharacterized protein ypbR OS=Bacillus subtilis GN=ypbR PE=4 SV=1 - [YPBR_BACSU]	1.35	44.44	3.02	3	3	60.08	3.77	6	4
**O34860**	RsbT co-antagonist protein rsbRB OS=Bacillus subtilis GN=rsbRB PE=1 SV=1 - [RSBRB_BACSU]	0.76	78.54	12.64	3	3	60.06	0.00	1	1
**P42305**	ATP-dependent RNA helicase dbpA OS=Bacillus subtilis GN=dbpA PE=1 SV=2 - [DBPA_BACSU]	#DIV/0!					59.98	8.35	7	4
**P39805**	Transcription antiterminator licT OS=Bacillus subtilis GN=licT PE=1 SV=1 - [LICT_BACSU]	0.62	95.85	11.91	3	3	59.80	9.75	2	2
**P39846**	Plipastatin synthase subunit B OS=Bacillus subtilis GN=ppsB PE=1 SV=1 - [PPSB_BACSU]	1.28	46.75	3.09	7	7	59.80	2.07	5	4
**P23448**	Flagellar motor switch protein fliG OS=Bacillus subtilis GN=fliG PE=3 SV=1 - [FLIG_BACSU]	0.94	62.63	12.43	4	4	58.76	9.76	4	3
**O31822**	Probable UTP--glucose-1-phosphate uridylyltransferase yngB OS=Bacillus subtilis GN=yngB PE=3 SV=1 - [YNGB_BACSU]	1.09	53.65	0.00	2	1	58.58	10.44	3	3

**P19672**	Uncharacterized protein yqxC OS=Bacillus subtilis GN=yqxC PE=4 SV=3 - [YQXC_BACSU]	1.26	46.54	0.00	1	1	58.51	13.88	4	4
**P39794**	PTS system trehalose-specific EIIBC component OS=Bacillus subtilis GN=treP PE=4 SV=3 - [PTTBC_BACSU]	1.05	55.30	5.53	7	2	57.80	9.15	4	3
**P09122**	DNA polymerase III subunit gamma/tau OS=Bacillus subtilis GN=dnaX PE=3 SV=3 - [DPO3X_BACSU]	1.64	35.25	0.00	1	1	57.72	3.91	2	2
**P23454**	FlaA locus 22.9 kDa protein OS=Bacillus subtilis GN=ylxF PE=4 SV=2 - [YLXF_BACSU]	2.01	28.47	10.78	4	2	57.21	12.25	4	2
**P94364**	Cytochrome d ubiquinol oxidase subunit 1 OS=Bacillus subtilis GN=cydA PE=3 SV=1 - [CYDA_BACSU]	1.02	55.28	0.00	4	1	56.39	3.85	3	2
**P39596**	UPF0409 protein ywbM OS=Bacillus subtilis GN=ywbM PE=3 SV=1 - [YWBM_BACSU]	0.59	95.59	11.95	5	4	56.21	0.00	2	1
**Q45068**	Amino-acid carrier protein alsT OS=Bacillus subtilis GN=alsT PE=2 SV=1 - [ALST_BACSU]	1.50	37.10	9.03	3	3	55.72	10.11	6	3
**P42297**	Uncharacterized protein yxiE OS=Bacillus subtilis GN=yxiE PE=3 SV=1 - [YXIE_BACSU]	0.97	57.56	26.35	3	3	55.72	17.57	4	2
**P23966**	Naphthoate synthase OS=Bacillus subtilis GN=menB PE=3 SV=2 - [MENB_BACSU]	0.20	279.01	26.57	8	5	55.64	10.33	2	2
**O31702**	Sulfur carrier protein moaD adenylyltransferase OS=Bacillus subtilis GN=moeB PE=3 SV=1 - [MOEB_BACSU]	0.36	152.13	18.88	10	6	54.83	10.32	4	3
**P17893**	Arginine repressor OS=Bacillus subtilis GN=argR PE=1 SV=1 - [ARGR_BACSU]	1.06	51.38	0.00	3	1	54.60	14.77	3	2
**O32068**	Uncharacterized RNA pseudouridine synthase ytzG OS=Bacillus subtilis GN=ytzG PE=3 SV=2 - [YTZG_BACSU]	0.81	67.33	15.48	3	3	54.60	15.48	4	3
**P45872**	Peptide chain release factor 1 OS=Bacillus subtilis GN=prfA PE=3 SV=1 - [RF1_BACSU]	0.42	129.75	14.89	6	4	53.94	8.99	2	2
**O34635**	Probable L-serine dehydratase, beta chain OS=Bacillus subtilis GN=sdaAB PE=3 SV=1 - [SDHB_BACSU]	0.56	95.50	27.73	6	5	53.68	21.36	4	4
**O31475**	Ferredoxin--NADP reductase 1 OS=Bacillus subtilis GN=ycgT PE=3 SV=1 - [FENR1_BACSU]	1.66	32.28	9.52	2	2	53.57	0.00	1	1
**O35024**	Manganese transport system membrane protein mntC OS=Bacillus subtilis GN=mntC PE=3 SV=1 - [MNTC_BACSU]	1.79	29.88	4.60	3	2	53.55	0.00	3	1
**P55339**	ABC-type transporter ATP-binding protein ecsA OS=Bacillus subtilis GN=ecsA PE=1 SV=1 - [ECSA_BACSU]	0.74	72.09	9.72	2	2	53.39	9.72	2	2
**O32223**	Uncharacterized oxidoreductase yvaA OS=Bacillus subtilis GN=yvaA PE=1 SV=1 - [YVAA_BACSU]	1.33	39.92	8.66	3	2	53.09	13.41	4	3
**P46918**	Minor teichoic acid biosynthesis protein ggaB OS=Bacillus subtilis GN=ggaB PE=3 SV=1 - [GGAB_BACSU]	0.47	112.02	5.44	11	5	52.63	3.44	3	3
**P42409**	RsbT co-antagonist protein rsbRA OS=Bacillus subtilis GN=rsbRA PE=1 SV=1 - [RSBRA_BACSU]	0.67	78.16	13.50	3	3	52.61	13.50	3	3
**P35162**	Cytochrome c biogenesis protein resC OS=Bacillus subtilis GN=resC PE=3 SV=2 - [RESC_BACSU]	0.79	66.42	9.46	7	3	52.19	0.00	1	1
**P42920**	50S ribosomal protein L3 OS=Bacillus subtilis GN=rplC PE=3 SV=2 - [RL3_BACSU]	0.33	156.33	22.49	4	3	51.58	22.49	3	3
**P22326**	Tyrosyl-tRNA synthetase 1 OS=Bacillus subtilis GN=tyrS1 PE=2 SV=2 - [SYY1_BACSU]	0.21	240.15	19.91	11	7	51.58	13.51	5	5
**P39071**	2,3-dihydro-2,3-dihydroxybenzoate dehydrogenase OS=Bacillus subtilis GN=dhbA PE=1 SV=3 - [DHBA_BACSU]	1.42	36.24	9.96	2	2	51.44	9.96	4	2
**O32099**	Benzil reductase OS=Bacillus subtilis GN=yueD PE=3 SV=1 - [BZRD_BACSU]	0.62	82.83	13.58	3	3	51.41	9.05	3	2
**O05220**	Uncharacterized protein ywrF OS=Bacillus subtilis GN=ywrF PE=3 SV=1 - [YWRF_BACSU]	0.28	184.00	17.56	5	3	51.39	17.56	3	3
**P94521**	Putative aminopeptidase ysdC OS=Bacillus subtilis GN=ysdC PE=1 SV=1 - [YSDC_BACSU]	0.52	98.40	9.97	4	3	51.28	0.00	1	1
**P71021**	Septum site-determining protein divIVA OS=Bacillus subtilis GN=divIVA PE=1 SV=1 - [DIV4A_BACSU]	0.38	133.43	16.46	4	2	51.23	16.46	2	2

**O34403**	Formamidopyrimidine-DNA glycosylase OS=Bacillus subtilis GN=mutM PE=3 SV=4 - [FPG_BACSU]	#DIV/0!					50.90	9.78	2	2
**P96716**	Tyrosine-protein kinase ywqD OS=Bacillus subtilis GN=ywqD PE=1 SV=1 - [YWQD_BACSU]	0.69	73.04	13.50	3	3	50.74	13.50	4	3
**P39847**	Plipastatin synthase subunit C OS=Bacillus subtilis GN=ppsC PE=1 SV=2 - [PPSC_BACSU]	0.62	81.20	3.29	11	6	50.52	0.00	3	3
**Q45477**	Isoleucyl-tRNA synthetase OS=Bacillus subtilis GN=ileS PE=3 SV=3 - [SYI_BACSU]	0.41	122.62	6.95	9	6	50.47	0.00	2	2
**P05096**	DNA primase OS=Bacillus subtilis GN=dnaG PE=3 SV=2 - [PRIM_BACSU]	#DIV/0!					50.45	4.15	2	2
**Q01960**	Flagellar biosynthesis protein flhF OS=Bacillus subtilis GN=flhF PE=1 SV=1 - [FLHF_BACSU]	0.62	80.78	6.01	5	2	50.45	0.00	1	1
**P40872**	Polyketide synthase pksM OS=Bacillus subtilis GN=pksM PE=1 SV=4 - [PKSM_BACSU]	#DIV/0!					50.39	1.10	4	4
**O05213**	UPF0214 protein ybbE OS=Bacillus subtilis GN=ybbE PE=3 SV=3 - [YBBE_BACSU]	0.67	74.70	13.38	7	6	50.36	4.54	2	2
**P46208**	Chaperone protein htpG OS=Bacillus subtilis GN=htpG PE=3 SV=1 - [HTPG_BACSU]	0.19	264.80	24.60	24	14	50.05	7.51	4	3
**Q04778**	HTH-type transcriptional regulator alsR OS=Bacillus subtilis GN=alsR PE=4 SV=1 - [ALSR_BACSU]	0.69	71.78	12.25	3	3	49.80	9.27	2	2
**O07597**	D-alanine aminotransferase OS=Bacillus subtilis GN=dat PE=3 SV=1 - [DAAA_BACSU]	0.93	53.55	13.83	3	3	49.73	24.82	5	5
**Q06796**	50S ribosomal protein L11 OS=Bacillus subtilis GN=rplK PE=3 SV=3 - [RL11_BACSU]	0.85	58.67	19.15	3	2	49.59	0.00	2	1
**P23974**	Putative esterase ytxM OS=Bacillus subtilis GN=ytxM PE=3 SV=2 - [YTXM_BACSU]	0.93	52.78	9.49	3	2	49.25	9.49	2	2
**O07605**	Proton/sodium-glutamate symport protein OS=Bacillus subtilis GN=gltT PE=3 SV=1 - [GLTT_BACSU]	0.36	135.38	3.73	8	2	49.25	0.00	5	1
**P42060**	50S ribosomal protein L22 OS=Bacillus subtilis GN=rplV PE=3 SV=1 - [RL22_BACSU]	0.41	118.68	23.01	4	2	49.15	0.00	1	1
**P39610**	Pyridoxine kinase OS=Bacillus subtilis GN=pdxK PE=1 SV=1 - [PDXK_BACSU]	0.78	62.66	16.24	6	3	49.08	11.07	4	2
**O34484**	Methionine aminopeptidase 2 OS=Bacillus subtilis GN=mapB PE=1 SV=1 - [AMPM2_BACSU]	1.70	28.89	10.04	3	2	49.06	0.00	2	1
**O34384**	Uncharacterized protein yceE OS=Bacillus subtilis GN=yceE PE=3 SV=1 - [YCEE_BACSU]	0.40	123.60	18.75	5	3	48.84	0.00	1	1
**P39648**	Uncharacterized protein ywfL OS=Bacillus subtilis GN=ywfL PE=4 SV=1 - [YWFL_BACSU]	1.13	42.38	9.25	2	2	47.69	0.00	1	1
**P96593**	Manganese transport protein mntH OS=Bacillus subtilis GN=mntH PE=1 SV=1 - [MNTH_BACSU]	1.82	26.03	0.00	1	1	47.29	5.18	3	2
**P12873**	50S ribosomal protein L29 OS=Bacillus subtilis GN=rpmC PE=3 SV=1 - [RL29_BACSU]	0.93	50.88	30.30	2	2	47.27	34.85	2	2
**P51831**	3-oxoacyl-[acyl-carrier-protein] reductase OS=Bacillus subtilis GN=fabG PE=3 SV=3 - [FABG_BACSU]	0.65	73.05	17.89	4	3	47.27	15.45	4	3
**O31751**	Undecaprenyl pyrophosphate synthetase OS=Bacillus subtilis GN=uppS PE=3 SV=1 - [UPPS_BACSU]	2.24	21.10	8.85	2	2	47.16	0.00	1	1
**O34471**	Uncharacterized protein ytlQ OS=Bacillus subtilis GN=ytlQ PE=4 SV=1 - [YTLQ_BACSU]	0.47	100.48	14.80	5	4	47.12	12.17	4	3
**P25813**	Ribosomal RNA small subunit methyltransferase G OS=Bacillus subtilis GN=rsmG PE=1 SV=1 - [RSMG_BACSU]	0.72	64.57	10.88	4	2	46.49	10.88	3	2
**O31425**	Sporulation-killing factor biosynthesis protein skfC OS=Bacillus subtilis GN=skfC PE=2 SV=2 - [SKFC_BACSU]	0.51	89.38	11.90	8	5	46.00	7.46	4	3
**P46910**	Probable transcription regulator arfM OS=Bacillus subtilis GN=arfM PE=2 SV=1 - [ARFM_BACSU]	1.03	44.73	21.52	6	3	45.97	0.00	3	1
**P50830**	Uncharacterized ATP-dependent helicase yprA OS=Bacillus subtilis GN=yprA PE=3 SV=1 - [YPRA_BACSU]	#DIV/0!					45.89	4.27	3	3

**P26900**	L-asparaginase 1 OS=Bacillus subtilis GN=ansA PE=2 SV=1 - [ASPG1_BACSU]	0.29	155.38	23.10	11	7	45.41	6.08	2	2
**P42176**	Nitrate reductase beta chain OS=Bacillus subtilis GN=narH PE=3 SV=1 - [NARH_BACSU]	0.40	114.44	9.24	8	5	45.33	8.21	4	3
**P42412**	Methylmalonate semialdehyde dehydrogenase [acylating] OS=Bacillus subtilis GN=iolA PE=1 SV=1 - [IOLA_BACSU]	0.38	118.16	9.03	4	3	45.32	6.37	4	2
**P39133**	Uncharacterized protein yhbF OS=Bacillus subtilis GN=yhbF PE=4 SV=3 - [YHBF_BACSU]	0.90	49.54	9.36	2	2	44.80	0.00	1	1
**O34768**	Uncharacterized sugar kinase ydjE OS=Bacillus subtilis GN=ydjE PE=3 SV=1 - [YDJE_BACSU]	1.00	44.87	8.75	3	2	44.73	13.44	4	3
**O05248**	Putative membrane protease yugP OS=Bacillus subtilis GN=yugP PE=4 SV=1 - [YUGP_BACSU]	1.43	31.21	0.00	3	2	44.73	8.44	3	2
**P40804**	Polyketide biosynthesis malonyl-ACP decarboxylase pksF OS=Bacillus subtilis GN=pksF PE=1 SV=3 - [PKSF_BACSU]	1.87	23.97	0.00	2	1	44.72	6.51	2	2
**P02394**	50S ribosomal protein L7/L12 OS=Bacillus subtilis GN=rplL PE=1 SV=4 - [RL7_BACSU]	0.25	176.54	47.15	7	6	44.68	39.02	4	4
**P32396**	Ferrochelatase OS=Bacillus subtilis GN=hemH PE=1 SV=1 - [HEMH_BACSU]	0.77	57.62	5.48	4	2	44.64	0.00	1	1
**O06724**	Uncharacterized protein yisK OS=Bacillus subtilis GN=yisK PE=2 SV=1 - [YISK_BACSU]	0.58	76.90	9.30	4	2	44.58	14.29	5	3
**P21477**	30S ribosomal protein S20 OS=Bacillus subtilis GN=rpsT PE=1 SV=4 - [RS20_BACSU]	0.39	112.47	17.05	4	2	44.17	17.05	3	2
**P96669**	Uncharacterized HTH-type transcriptional regulator ydeL OS=Bacillus subtilis GN=ydeL PE=3 SV=1 - [YDEL_BACSU]	#DIV/0!					44.13	4.97	3	2
**P37545**	Uncharacterized deoxyribonuclease yabD OS=Bacillus subtilis GN=yabD PE=3 SV=1 - [YABD_BACSU]	0.44	98.19	23.53	5	5	43.61	8.24	2	2
**P39134**	Protein prkA OS=Bacillus subtilis GN=prkA PE=3 SV=1 - [PRKA_BACSU]	0.36	119.69	10.94	8	6	43.51	5.71	3	3
**P39580**	Protein dltB OS=Bacillus subtilis GN=dltB PE=1 SV=1 - [DLTB_BACSU]	0.30	142.08	7.34	8	3	42.74	4.30	3	2
**P38423**	Uncharacterized protein yqxK OS=Bacillus subtilis GN=yqxK PE=4 SV=3 - [YQXK_BACSU]	0.89	47.71	0.00	1	1	42.66	5.68	3	2
**P21470**	30S ribosomal protein S9 OS=Bacillus subtilis GN=rpsI PE=1 SV=3 - [RS9_BACSU]	0.57	74.44	15.38	3	2	42.50	15.38	2	2
**Q00777**	Pyrroline-5-carboxylate reductase 3 OS=Bacillus subtilis GN=proG PE=3 SV=2 - [P5CR3_BACSU]	0.56	75.56	15.07	5	4	42.30	8.09	2	2
**P37464**	Seryl-tRNA synthetase OS=Bacillus subtilis GN=serS PE=3 SV=1 - [SYS_BACSU]	0.86	49.03	7.29	4	3	42.19	5.65	2	2
**P37563**	tRNA(Ile)-lysidine synthase OS=Bacillus subtilis GN=tilS PE=1 SV=2 - [TILS_BACSU]	#DIV/0!					42.12	6.99	3	2
**P80880**	Thioredoxin reductase OS=Bacillus subtilis GN=trxB PE=1 SV=3 - [TRXB_BACSU]	0.67	62.46	7.28	2	2	42.00	0.00	1	1
**P94461**	Primosomal protein N' OS=Bacillus subtilis GN=priA PE=3 SV=2 - [PRIA_BACSU]	#DIV/0!					41.98	7.08	4	4
**P14802**	Uncharacterized oxidoreductase yoxD OS=Bacillus subtilis GN=yoxD PE=3 SV=2 - [YOXD_BACSU]	0.56	75.06	16.39	3	3	41.68	9.24	2	2
**O32022**	Uncharacterized protein yqzD OS=Bacillus subtilis GN=yqzD PE=4 SV=1 - [YQZD_BACSU]	0.73	56.78	0.00	1	1	41.46	25.64	2	2
**O06974**	UPF0052 protein yvcK OS=Bacillus subtilis GN=yvcK PE=3 SV=1 - [YVCK_BACSU]	0.48	84.69	0.00	3	1	40.80	14.51	4	3
**O06746**	UPF0234 protein yitk OS=Bacillus subtilis GN=yitK PE=3 SV=1 - [YITK_BACSU]	1.99	20.55	16.56	2	2	40.79	14.72	2	2
**O31974**	SPBc2 prophage-derived uncharacterized protein yomK OS=Bacillus subtilis GN=yomK PE=4 SV=1 - [YOMK_BACSU]	0.46	89.37	16.89	3	3	40.75	14.19	3	2
**P56849**	50S ribosomal protein L33 1 OS=Bacillus subtilis GN=rpmGA PE=3 SV=2 - [RL331_BACSU]	0.49	83.39	0.00	2	1	40.59	44.90	2	2

**P17731**	Histidinol-phosphate aminotransferase OS=Bacillus subtilis GN=hisC PE=3 SV=3 - [HIS8_BACSU]	0.38	107.64	14.44	5	4	40.55	7.22	2	2
**P94550**	Electron transfer flavoprotein subunit beta OS=Bacillus subtilis GN=etfB PE=3 SV=1 - [ETFB_BACSU]	0.60	67.29	11.28	2	2	40.42	21.01	4	3
**P96596**	Uncharacterized protein ydbA OS=Bacillus subtilis GN=ydbA PE=4 SV=1 - [YDBA_BACSU]	#DIV/0!					40.09	13.24	3	3
**P16655**	Division initiation protein OS=Bacillus subtilis GN=divIB PE=3 SV=1 - [DIVIB_BACSU]	#DIV/0!					40.01	9.13	2	2
**P42065**	Oligopeptide transport ATP-binding protein appF OS=Bacillus subtilis GN=appF PE=3 SV=2 - [APPF_BACSU]	1.08	36.92	9.42	3	3	39.76	6.38	2	2
**O34442**	Magnesium transporter mgtE OS=Bacillus subtilis GN=mgtE PE=3 SV=1 - [MGTE_BACSU]	0.76	51.61	6.65	3	2	39.44	0.00	1	1
**P37540**	DNA polymerase III subunit delta' OS=Bacillus subtilis GN=holB PE=3 SV=1 - [HOLB_BACSU]	0.84	46.63	0.00	1	1	39.31	9.73	2	2
**P18256**	Threonyl-tRNA synthetase 2 OS=Bacillus subtilis GN=thrZ PE=2 SV=2 - [SYT2_BACSU]	1.31	29.76	0.00	1	1	39.10	4.39	2	2
**P18159**	Phosphoglucomutase OS=Bacillus subtilis GN=pgcA PE=1 SV=3 - [PGCA_BACSU]	0.34	114.85	11.70	8	6	38.70	0.00	1	1
**O07567**	NTD biosynthesis operon regulator ntdR OS=Bacillus subtilis GN=ntdR PE=4 SV=1 - [NTDR_BACSU]	1.65	23.04	0.00	1	1	38.04	10.33	3	3
**P37522**	Sporulation initiation inhibitor protein soj OS=Bacillus subtilis GN=soj PE=1 SV=1 - [SOJ_BACSU]	0.25	149.61	28.46	10	6	37.97	16.60	5	4
**P25972**	Orotate phosphoribosyltransferase OS=Bacillus subtilis GN=pyrE PE=3 SV=1 - [PYRE_BACSU]	0.60	63.21	10.19	3	2	37.84	10.19	2	2
**P81100**	Stress response protein SCP2 OS=Bacillus subtilis GN=yceC PE=1 SV=3 - [SCP2_BACSU]	0.29	127.65	27.64	6	5	37.37	15.58	3	3
**P26899**	Aspartate ammonia-lyase OS=Bacillus subtilis GN=ansB PE=3 SV=2 - [ASPA_BACSU]	0.12	316.39	14.95	17	6	37.25	4.42	3	2
**Q45589**	Uncharacterized protein ybbP OS=Bacillus subtilis GN=ybbP PE=4 SV=3 - [YBBP_BACSU]	1.61	22.96	10.26	2	2	36.86	9.52	2	2
**O32082**	Uncharacterized membrane protein yubF OS=Bacillus subtilis GN=yubF PE=4 SV=1 - [YUBF_BACSU]	0.33	111.51	17.24	4	2	36.61	0.00	2	1
**O05251**	Transcriptional regulatory protein malR OS=Bacillus subtilis GN=malR PE=3 SV=1 - [MALR_BACSU]	1.04	35.34	8.51	2	2	36.59	8.94	2	2
**O34328**	Guanylate kinase OS=Bacillus subtilis GN=gmk PE=3 SV=2 - [KGUA_BACSU]	1.08	33.68	11.27	2	2	36.44	0.00	1	1
**P54461**	Ribosomal RNA small subunit methyltransferase E OS=Bacillus subtilis GN=rsmE PE=1 SV=1 - [RSME_BACSU]	0.90	39.86	15.23	4	3	35.81	8.98	2	2
**P46354**	Purine nucleoside phosphorylase 1 OS=Bacillus subtilis GN=punA PE=1 SV=1 - [PUNA_BACSU]	0.31	115.64	15.50	2	2	35.68	0.00	1	1
**O32247**	Uncharacterized membrane protein yvbJ OS=Bacillus subtilis GN=yvbJ PE=4 SV=1 - [YVBJ_BACSU]	#DIV/0!					35.64	4.79	2	2
**O05514**	Thiamine-monophosphate kinase OS=Bacillus subtilis GN=thiL PE=3 SV=2 - [THIL_BACSU]	0.28	127.05	17.54	11	5	35.61	7.69	2	2
**P55874**	50S ribosomal protein L35 OS=Bacillus subtilis GN=rpmI PE=3 SV=2 - [RL35_BACSU]	0.76	46.68	34.85	3	2	35.48	0.00	2	1
**Q45600**	Uncharacterized metallophosphoesterase-like protein yydB OS=Bacillus subtilis GN=yydB PE=3 SV=1 - [YYDB_BACSU]	0.76	46.26	9.77	4	4	35.18	0.00	1	1
**P39773**	2,3-bisphosphoglycerate-independent phosphoglycerate mutase OS=Bacillus subtilis GN=gpmI PE=1 SV=4 - [GPMI_BACSU]	0.14	253.86	18.40	13	8	35.09	4.70	2	2
**P54510**	Uncharacterized protein yqhL OS=Bacillus subtilis GN=yqhL PE=4 SV=2 - [YQHL_BACSU]	1.13	30.99	0.00	1	1	35.07	25.40	4	3
**O31605**	Oligoendopeptidase F homolog OS=Bacillus subtilis GN=yjbG PE=3 SV=2 - [PEPF_BACSU]	0.25	141.49	10.45	7	5	34.86	0.00	3	1
**O31427**	SkfA peptide export ATP-binding protein skfE OS=Bacillus subtilis GN=skfE PE=2 SV=1 - [SKFE_BACSU]	1.66	20.73	0.00	1	1	34.47	10.04	2	2

**P39802**	Chemotaxis protein cheW OS=Bacillus subtilis GN=cheW PE=4 SV=1 - [CHEW_BACSU]	0.38	90.55	36.54	5	5	34.40	15.38	2	2
**O34707**	Uncharacterized protein ytpB OS=Bacillus subtilis GN=ytpB PE=4 SV=1 - [YTPB_BACSU]	0.30	114.46	12.26	7	4	34.40	0.00	2	1
**P32395**	Uroporphyrinogen decarboxylase OS=Bacillus subtilis GN=hemE PE=1 SV=1 - [DCUP_BACSU]	0.48	71.88	5.95	5	2	34.28	5.95	2	2
**O05261**	Uncharacterized HTH-type transcriptional regulator yulB OS=Bacillus subtilis GN=yulB PE=4 SV=1 - [YULB_BACSU]	0.70	48.44	7.75	3	2	34.08	0.00	1	1
**O32188**	Probable siderophore transport system ATP-binding protein yusV OS=Bacillus subtilis GN=yusV PE=1 SV=1 - [YUSV_BACSU]	0.49	69.49	10.55	5	3	34.04	7.27	2	2
**P08164**	NH(3)-dependent NAD(+) synthetase OS=Bacillus subtilis GN=nadE PE=1 SV=5 - [NADE_BACSU]	0.35	95.90	12.13	4	2	33.81	0.00	1	1
**P39795**	Trehalose-6-phosphate hydrolase OS=Bacillus subtilis GN=treA PE=1 SV=2 - [TREC_BACSU]	0.47	72.28	3.57	2	2	33.80	4.28	2	2
**P20964**	GTPase obgE OS=Bacillus subtilis GN=obg PE=1 SV=1 - [OBG_BACSU]	0.41	81.55	6.54	4	2	33.64	0.00	2	1
**O34714**	Oxalate decarboxylase oxdC OS=Bacillus subtilis GN=oxdC PE=1 SV=1 - [OXDC_BACSU]	0.34	99.31	12.73	7	4	33.43	0.00	1	1
**O34934**	Probable inorganic polyphosphate/ATP-NAD kinase 2 OS=Bacillus subtilis GN=ppnK2 PE=3 SV=1 - [PPNK2_BACSU]	0.83	40.13	18.73	3	3	33.27	0.00	2	1
**P32726**	Ribosome maturation factor rimP OS=Bacillus subtilis GN=rimP PE=3 SV=1 - [RIMP_BACSU]	0.41	78.91	16.03	6	3	32.31	0.00	1	1
**O31503**	Uncharacterized RNA methyltransferase yefA OS=Bacillus subtilis GN=yefA PE=3 SV=1 - [YEFA_BACSU]	0.25	130.21	9.59	6	4	32.26	4.58	2	2
**O34774**	Uncharacterized protein yobJ OS=Bacillus subtilis GN=yobJ PE=4 SV=1 - [YOBJ_BACSU]	0.77	41.31	7.50	3	2	31.83	10.71	3	3
**P39616**	Probable aldehyde dehydrogenase ywdH OS=Bacillus subtilis GN=ywdH PE=3 SV=2 - [ALDH2_BACSU]	0.93	34.15	3.95	2	2	31.61	7.46	3	2
**O32106**	Probable cytosol aminopeptidase OS=Bacillus subtilis GN=pepA PE=3 SV=1 - [AMPA_BACSU]	0.33	96.62	12.00	7	5	31.56	4.60	2	2
**O05519**	Uncharacterized ABC transporter ATP-binding protein ydiF OS=Bacillus subtilis GN=ydiF PE=3 SV=2 - [YDIF_BACSU]	#DIV/0!					31.48	4.05	2	2
**P37478**	Transcriptional regulatory protein yycF OS=Bacillus subtilis GN=yycF PE=1 SV=1 - [YYCF_BACSU]	0.63	49.42	6.81	3	2	31.31	11.06	2	2
**P23478**	ATP-dependent helicase/nuclease subunit A OS=Bacillus subtilis GN=addA PE=1 SV=2 - [ADDA_BACSU]	0.83	36.93	0.00	1	1	30.77	1.87	2	2
**O05496**	Uncharacterized UDP-glucosyltransferase ydhE OS=Bacillus subtilis GN=ydhE PE=3 SV=2 - [YDHE_BACSU]	0.65	46.16	7.59	3	3	30.11	7.09	2	2
**O05392**	Holliday junction ATP-dependent DNA helicase ruvA OS=Bacillus subtilis GN=ruvA PE=3 SV=2 - [RUVA_BACSU]	0.55	54.59	10.95	2	2	30.01	0.00	1	1
**P54375**	Superoxide dismutase [Mn] OS=Bacillus subtilis GN=sodA PE=1 SV=5 - [SODM_BACSU]	0.26	114.73	19.80	4	3	30.00	10.89	2	2
**P39812**	Glutamate synthase [NADPH] large chain OS=Bacillus subtilis GN=gltA PE=2 SV=3 - [GLTA_BACSU]	0.99	30.15	2.17	3	3	29.98	3.49	4	4
**P52996**	3-methyl-2-oxobutanoate hydroxymethyltransferase OS=Bacillus subtilis GN=panB PE=3 SV=1 - [PANB_BACSU]	0.27	111.16	16.97	3	3	29.59	11.91	2	2
**P46913**	Menaquinol-cytochrome c reductase cytochrome b/c subunit OS=Bacillus subtilis GN=qcrC PE=3 SV=1 - [QCRC_BACSU]	0.54	53.92	5.88	2	2	28.92	5.88	2	2
**P37535**	Uncharacterized protein yaaN OS=Bacillus subtilis GN=yaaN PE=3 SV=1 - [YAAN_BACSU]	0.29	99.63	11.66	4	4	28.79	5.96	2	2
**C0SP98**	Putative oligopeptide transport ATP-binding protein ykfD OS=Bacillus subtilis GN=ykfD PE=3 SV=1 - [YKFD_BACSU]	1.20	24.08	0.00	1	1	28.78	6.69	2	2
**P19946**	50S ribosomal protein L15 OS=Bacillus subtilis GN=rplO PE=3 SV=1 - [RL15_BACSU]	0.37	76.64	29.45	4	3	28.62	0.00	1	1
**P54716**	Maltose-6'-phosphate glucosidase OS=Bacillus subtilis GN=glvA PE=1 SV=1 - [GLVA_BACSU]	0.84	33.52	4.45	2	2	28.30	4.90	2	2

**P39821**	Gamma-glutamyl phosphate reductase OS=Bacillus subtilis GN=proA PE=3 SV=3 - [PROA_BACSU]	0.10	275.66	23.37	20	8	28.07	0.00	2	1
**O32036**	Putative O-methyltransferase yrrM OS=Bacillus subtilis GN=yrrM PE=3 SV=1 - [YRRM_BACSU]	0.46	59.91	12.90	3	2	27.68	11.98	2	2
**P32731**	Ribosome-binding factor A OS=Bacillus subtilis GN=rbfA PE=3 SV=1 - [RBFA_BACSU]	0.89	30.52	0.00	1	1	27.22	17.09	2	2
**O31989**	SPBc2 prophage-derived uncharacterized protein yolF OS=Bacillus subtilis GN=yolF PE=4 SV=1 - [YOLF_BACSU]	0.45	60.77	22.86	2	2	27.21	0.00	1	1
**Q06752**	Cysteinyl-tRNA synthetase OS=Bacillus subtilis GN=cysS PE=1 SV=1 - [SYC_BACSU]	0.25	109.15	18.03	10	7	27.19	7.51	3	3
**O32210**	Glyoxal reductase OS=Bacillus subtilis GN=yvgN PE=1 SV=1 - [GR_BACSU]	0.26	103.65	13.77	5	3	27.18	10.14	2	2
**P39756**	Protein fdhD homolog OS=Bacillus subtilis GN=fdhD PE=3 SV=1 - [FDHD_BACSU]	#DIV/0!					27.08	8.02	3	2
**O31418**	Putative ribonuclease mrnC OS=Bacillus subtilis GN=mrnC PE=4 SV=1 - [MRNC_BACSU]	0.47	56.92	18.88	2	2	27.00	15.38	2	2
**O05412**	Glutamate racemase 2 OS=Bacillus subtilis GN=yrpC PE=1 SV=1 - [MURI2_BACSU]	#DIV/0!					26.96	8.30	2	2
**P54473**	4-hydroxy-3-methylbut-2-enyl diphosphate reductase OS=Bacillus subtilis GN=ispH PE=3 SV=2 - [ISPH_BACSU]	0.61	43.90	8.28	2	2	26.94	0.00	1	1
**O34499**	Uncharacterized protein ykgB OS=Bacillus subtilis GN=ykgB PE=3 SV=1 - [YKGB_BACSU]	0.81	33.14	0.00	1	1	26.83	10.60	2	2
**P37529**	Deoxyadenosine/deoxycytidine kinase OS=Bacillus subtilis GN=dck PE=1 SV=1 - [DCK_BACSU]	0.45	58.20	11.06	2	2	26.27	0.00	1	1
**P05651**	DNA replication and repair protein recF OS=Bacillus subtilis GN=recF PE=3 SV=2 - [RECF_BACSU]	1.05	24.96	0.00	1	1	26.21	6.49	2	2
**P37813**	ATP synthase subunit a OS=Bacillus subtilis GN=atpB PE=3 SV=1 - [ATP6_BACSU]	0.52	49.93	9.43	3	2	25.95	0.00	2	1
**P39645**	UPF0447 protein ywfI OS=Bacillus subtilis GN=ywfI PE=3 SV=1 - [YWFI_BACSU]	0.30	87.65	17.32	7	4	25.90	8.27	2	2
**O34586**	Uncharacterized membrane protein ylbC OS=Bacillus subtilis GN=ylbC PE=4 SV=1 - [YLBC_BACSU]	0.68	37.61	12.43	4	3	25.39	8.96	2	2
**P14194**	General stress protein CTC OS=Bacillus subtilis GN=ctc PE=1 SV=4 - [CTC_BACSU]	0.45	56.06	21.08	5	3	25.39	15.20	3	2
**P12046**	Phosphoribosylaminoimidazole-succinocarboxamide synthase OS=Bacillus subtilis GN=purC PE=1 SV=1 - [PUR7_BACSU]	0.32	78.07	9.54	3	2	25.27	0.00	1	1
**O31446**	Uncharacterized protein ybfF OS=Bacillus subtilis GN=ybfF PE=4 SV=1 - [YBFF_BACSU]	0.69	36.87	10.56	4	3	25.26	0.00	1	1
**O34347**	Argininosuccinate synthase OS=Bacillus subtilis GN=argG PE=3 SV=1 - [ASSY_BACSU]	0.17	150.87	19.60	9	7	25.13	5.46	2	2
**P54518**	Uncharacterized peptidase yqhT OS=Bacillus subtilis GN=yqhT PE=3 SV=1 - [YQHT_BACSU]	0.24	106.11	8.22	3	2	24.97	0.00	2	1
**P46906**	Arginyl-tRNA synthetase OS=Bacillus subtilis GN=argS PE=3 SV=2 - [SYR_BACSU]	0.22	111.15	10.79	8	5	24.80	5.22	2	2
**O34353**	Uncharacterized protein ydjN OS=Bacillus subtilis GN=ydjN PE=4 SV=1 - [YDJN_BACSU]	0.80	30.77	8.91	3	3	24.49	0.00	1	1
**C0SP93**	Acetyl-coenzyme A carboxylase carboxyl transferase subunit beta OS=Bacillus subtilis GN=accD PE=1 SV=1 - [ACCD_BACSU]	0.53	46.28	6.55	3	2	24.46	0.00	2	1
**O06996**	Putative sporulation hydrolase cotR OS=Bacillus subtilis GN=cotR PE=2 SV=1 - [COTR_BACSU]	0.54	44.84	8.44	3	2	24.43	9.69	2	2
**O34558**	SPBc2 prophage-derived uncharacterized protein yopR OS=Bacillus subtilis GN=yopR PE=4 SV=2 - [YOPR_BACSU]	0.44	55.41	6.77	3	2	24.41	0.00	1	1
**P32397**	Protoporphyrinogen oxidase OS=Bacillus subtilis GN=hemY PE=1 SV=1 - [PPOX_BACSU]	0.20	119.96	8.30	6	3	24.32	0.00	1	1
**O31630**	Uncharacterized protein yjcH OS=Bacillus subtilis GN=yjcH PE=4 SV=1 - [YJCH_BACSU]	0.84	29.00	11.67	2	2	24.30	0.00	1	1

**P80876**	General stress protein 18 OS=Bacillus subtilis GN=yfkM PE=1 SV=3 - [GS18_BACSU]	0.31	77.00	19.19	4	3	24.07	0.00	1	1
**P94535**	Glycolate oxidase subunit glcD OS=Bacillus subtilis GN=glcD PE=3 SV=1 - [GLCD_BACSU]	0.14	168.34	9.36	6	3	23.86	0.00	1	1
**O31611**	GTP pyrophosphokinase yjbM OS=Bacillus subtilis GN=yjbM PE=1 SV=1 - [YJBM_BACSU]	0.92	25.78	0.00	1	1	23.84	9.00	2	2
**P38494**	30S ribosomal protein S1 homolog OS=Bacillus subtilis GN=ypfD PE=1 SV=1 - [RS1H_BACSU]	0.03	782.95	38.22	38	14	23.77	6.28	3	2
**P12047**	Adenylosuccinate lyase OS=Bacillus subtilis GN=purB PE=1 SV=1 - [PUR8_BACSU]	0.69	34.32	8.12	4	3	23.75	3.48	3	2
**O34876**	Cell division protein ftsX OS=Bacillus subtilis GN=ftsX PE=1 SV=1 - [FTSX_BACSU]	0.22	107.95	10.81	7	3	23.61	0.00	1	1
**P46343**	PhoH-like protein OS=Bacillus subtilis GN=phoH PE=3 SV=3 - [PHOL_BACSU]	0.37	63.04	10.97	5	3	23.43	0.00	2	1
**O07584**	1-acyl-sn-glycerol-3-phosphate acyltransferase OS=Bacillus subtilis GN=plsC PE=3 SV=1 - [PLSC_BACSU]	0.93	25.20	18.59	3	3	23.32	14.07	2	2
**P45913**	Uncharacterized protein yqaP OS=Bacillus subtilis GN=yqaP PE=4 SV=1 - [YQAP_BACSU]	0.41	57.15	19.09	5	5	23.22	0.00	1	1
**O35025**	Type-2 restriction enzyme BsuMI component ydiR OS=Bacillus subtilis GN=ydiR PE=2 SV=1 - [YDIR_BACSU]	0.81	27.89	7.35	3	2	22.47	0.00	1	1
**O34530**	Putative ribosome biogenesis GTPase rsgA OS=Bacillus subtilis GN=rsgA PE=1 SV=1 - [RSGA_BACSU]	0.43	51.71	0.00	4	1	22.24	8.72	2	2
**P39121**	Deoxyribose-phosphate aldolase OS=Bacillus subtilis GN=deoC PE=3 SV=3 - [DEOC_BACSU]	0.19	117.37	12.11	3	2	21.92	0.00	1	1
**P94421**	Uncharacterized ABC transporter solute-binding protein yclQ OS=Bacillus subtilis GN=yclQ PE=1 SV=1 - [YCLQ_BACSU]	0.38	57.86	16.72	5	4	21.75	0.00	1	1
**O31937**	SPBc2 prophage-derived uncharacterized protein yopA OS=Bacillus subtilis GN=yopA PE=4 SV=1 - [YOPA_BACSU]	0.15	142.81	16.67	8	6	21.65	0.00	1	1
**P14203**	Uncharacterized protein yuxH OS=Bacillus subtilis GN=yuxH PE=4 SV=1 - [YUXH_BACSU]	0.90	23.87	5.13	2	2	21.42	0.00	1	1
**P37887**	Cysteine synthase OS=Bacillus subtilis GN=cysK PE=1 SV=3 - [CYSK_BACSU]	0.23	92.22	19.16	7	4	21.32	0.00	2	1
**P05648**	Chromosomal replication initiator protein dnaA OS=Bacillus subtilis GN=dnaA PE=1 SV=1 - [DNAA_BACSU]	0.00	37.17	5.61	2	2				
**Q01625**	Membrane protein oxaA 1 OS=Bacillus subtilis GN=oxaA1 PE=2 SV=2 - [OXAA1_BACSU]	0.00	53.40	8.43	2	2				
**P12876**	50S ribosomal protein L24 OS=Bacillus subtilis GN=rplX PE=1 SV=1 - [RL24_BACSU]	0.00	46.38	19.42	2	2				
**P16616**	Porphobilinogen deaminase OS=Bacillus subtilis GN=hemC PE=3 SV=3 - [HEM3_BACSU]	0.00	41.61	8.28	2	2				
**P16304**	Adenylate kinase OS=Bacillus subtilis GN=adk PE=1 SV=2 - [KAD_BACSU]	0.00	52.52	11.98	2	2				
**P20691**	3-phosphoshikimate 1-carboxyvinyltransferase OS=Bacillus subtilis GN=aroE PE=3 SV=1 - [AROA_BACSU]	0.00	29.21	5.61	2	2				
**P28015**	Putative septation protein spoVG OS=Bacillus subtilis GN=spoVG PE=1 SV=1 - [SP5G_BACSU]	0.00	78.53	26.80	3	2				
**P14949**	Thioredoxin OS=Bacillus subtilis GN=trxA PE=1 SV=3 - [THIO_BACSU]	0.00	72.64	25.00	2	2				
**P31103**	Nucleoside diphosphate kinase OS=Bacillus subtilis GN=ndk PE=1 SV=3 - [NDK_BACSU]	0.00	70.67	14.09	2	2				
**P25152**	Uncharacterized peptidase ywaD OS=Bacillus subtilis GN=ywaD PE=3 SV=2 - [YWAD_BACSU]	0.00	68.75	5.27	3	2				
**Q45604**	Uncharacterized zinc-type alcohol dehydrogenase-like protein yycR OS=Bacillus subtilis GN=yycR PE=3 SV=1 - [YYCR_BACSU]	0.00	142.63	7.11	5	2				
**P42982**	Uncharacterized glycosyltransferase ypjH OS=Bacillus subtilis GN=ypjH PE=3 SV=2 - [YPJH_BACSU]	0.00	48.98	6.37	3	2				

**P54169**	Uncharacterized protein ypgR OS=Bacillus subtilis GN=ypgR PE=4 SV=1 - [YPGR_BACSU]	0.00	73.70	7.16	6	2
**P54517**	3-dehydroquinate dehydratase OS=Bacillus subtilis GN=yqhS PE=1 SV=1 - [AROQ_BACSU]	0.00	42.01	14.86	2	2
**P54534**	UPF0403 protein yqiW OS=Bacillus subtilis GN=yqiW PE=3 SV=1 - [YQIW_BACSU]	0.00	26.49	17.93	2	2
**P94514**	Sensory transduction protein lytT OS=Bacillus subtilis GN=lytT PE=3 SV=1 - [LYTT_BACSU]	0.00	41.87	8.71	3	2
**P94365**	Cytochrome d ubiquinol oxidase subunit 2 OS=Bacillus subtilis GN=cydB PE=3 SV=1 - [CYDB_BACSU]	0.00	39.85	5.33	3	2
**P96574**	Transcriptional regulator mtlR OS=Bacillus subtilis GN=mtlR PE=2 SV=1 - [MTLR_BACSU]	0.00	82.53	3.75	3	2
**P96672**	UPF0750 membrane protein ydeO OS=Bacillus subtilis GN=ydeO PE=3 SV=1 - [YDEO_BACSU]	0.00	22.10	6.21	2	2
**P96718**	UDP-glucose 6-dehydrogenase ywqF OS=Bacillus subtilis GN=ywqF PE=1 SV=1 - [YWQF_BACSU]	0.00	55.74	5.00	3	2
**O05250**	Sensor histidine kinase malK OS=Bacillus subtilis GN=malK PE=3 SV=1 - [MALK_BACSU]	0.00	31.00	3.75	2	2
**O06980**	Uncharacterized ABC transporter ATP-binding protein yvcR OS=Bacillus subtilis GN=yvcR PE=3 SV=1 - [YVCR_BACSU]	0.00	26.84	8.49	2	2
**O07516**	Uncharacterized membrane protein yhaH OS=Bacillus subtilis GN=yhaH PE=4 SV=1 - [YHAH_BACSU]	0.00	49.78	20.34	2	2
**O08455**	Uncharacterized protein yhaN OS=Bacillus subtilis GN=yhaN PE=2 SV=1 - [YHAN_BACSU]	0.00	45.09	2.80	4	2
**O07527**	Sensor histidine kinase yhcY OS=Bacillus subtilis GN=yhcY PE=3 SV=1 - [YHCY_BACSU]	0.00	24.78	6.07	2	2
**O07609**	Uncharacterized sugar epimerase yhfK OS=Bacillus subtilis GN=yhfK PE=2 SV=1 - [YHFK_BACSU]	0.00	36.29	12.62	2	2
**O34385**	Manganese-binding lipoprotein mntA OS=Bacillus subtilis GN=mntA PE=3 SV=1 - [MNTA_BACSU]	0.00	85.99	8.17	3	2
**P40768**	Uncharacterized protein ytkA OS=Bacillus subtilis GN=ytkA PE=4 SV=2 - [YTKA_BACSU]	0.00	75.10	23.45	3	2
**P80879**	General stress protein 20U OS=Bacillus subtilis GN=dps PE=1 SV=3 - [G20U_BACSU]	0.00	30.25	16.55	2	2
**O34618**	Uncharacterized amino acid permease ytnA OS=Bacillus subtilis GN=ytnA PE=3 SV=1 - [YTNA_BACSU]	0.00	42.15	4.32	3	2
**O34697**	Bacitracin export ATP-binding protein bceA OS=Bacillus subtilis GN=bceA PE=2 SV=1 - [BCEA_BACSU]	0.00	33.75	9.09	2	2
**O34944**	Putative dipeptidase ytjP OS=Bacillus subtilis GN=ytjP PE=3 SV=1 - [PEPVL_BACSU]	0.00	41.75	6.26	2	2
**O34424**	Uncharacterized membrane protein yteJ OS=Bacillus subtilis GN=yteJ PE=4 SV=1 - [YTEJ_BACSU]	0.00	64.18	11.59	4	2
**P80864**	Probable thiol peroxidase OS=Bacillus subtilis GN=tpx PE=1 SV=3 - [TPX_BACSU]	0.00	71.66	13.17	3	2
**O34680**	Probable BsuMI modification methylase subunit ydiP OS=Bacillus subtilis GN=ydiP PE=2 SV=1 - [YDIP_BACSU]	0.00	94.27	5.66	5	2
**O34394**	Uncharacterized protein yjjA OS=Bacillus subtilis GN=yjjA PE=4 SV=1 - [YJJA_BACSU]	0.00	43.08	9.63	4	2
**O34334**	Uncharacterized protein yjoA OS=Bacillus subtilis GN=yjoA PE=1 SV=1 - [YJOA_BACSU]	0.00	52.94	14.94	2	2
**O34703**	Uncharacterized ATPase yjoB OS=Bacillus subtilis GN=yjoB PE=1 SV=1 - [YJOB_BACSU]	0.00	50.61	6.15	2	2
**O34814**	Cell division ATP-binding protein ftsE OS=Bacillus subtilis GN=ftsE PE=1 SV=1 - [FTSE_BACSU]	0.00	27.55	10.53	2	2
**O34475**	Putative NAD(P)H nitroreductase yfkO OS=Bacillus subtilis GN=yfkO PE=1 SV=1 - [YFKO_BACSU]	0.00	35.54	9.05	2	2

**O34717**	Probable 2,4-dienoyl-CoA reductase OS=Bacillus subtilis GN=fadH PE=2 SV=1 - [FADH_BACSU]	0.00	69.61	5.51	2	2
**O31457**	UPF0176 protein ybfQ OS=Bacillus subtilis GN=ybfQ PE=3 SV=1 - [YBFQ_BACSU]	0.00	74.90	7.45	3	2
**P24138**	Oligopeptide transport system permease protein oppB OS=Bacillus subtilis GN=oppB PE=2 SV=2 - [OPPB_BACSU]	0.00	60.69	8.04	3	2
**O31629**	UPF0477 protein yjcG OS=Bacillus subtilis GN=yjcG PE=1 SV=1 - [YJCG_BACSU]	0.00	32.04	13.45	2	2
**O31635**	Uncharacterized protein yjcM OS=Bacillus subtilis GN=yjcM PE=4 SV=1 - [YJCM_BACSU]	0.00	30.38	5.38	2	2
**O31638**	Uncharacterized protein yjcP OS=Bacillus subtilis GN=yjcP PE=4 SV=1 - [YJCP_BACSU]	0.00	38.63	12.57	2	2
**O31754**	Zinc metalloprotease rasP OS=Bacillus subtilis GN=rasP PE=1 SV=1 - [RASP_BACSU]	0.00	143.28	5.21	5	2
**O32003**	SPBc2 prophage-derived aminoglycoside N(3')-acetyltransferase-like protein yokD OS=Bacillus subtilis GN=yokD PE=1 SV=1 - [YOKD_BACSU]	0.00	32.57	9.19	3	2
**O32031**	Uncharacterized membrane protein yrrS OS=Bacillus subtilis GN=yrrS PE=4 SV=1 - [YRRS_BACSU]	0.00	99.39	12.88	5	2
**O32052**	UPF0092 membrane protein yrbF OS=Bacillus subtilis GN=yrbF PE=3 SV=1 - [YRBF_BACSU]	0.00	33.22	24.72	2	2
**O32053**	Queuine tRNA-ribosyltransferase OS=Bacillus subtilis GN=tgt PE=3 SV=1 - [TGT_BACSU]	0.00	30.65	5.51	3	2
**O32148**	Purine catabolism protein pucG OS=Bacillus subtilis GN=pucG PE=2 SV=1 - [PUCG_BACSU]	0.00	40.90	5.77	5	2
**O32192**	Transcriptional regulatory protein cssR OS=Bacillus subtilis GN=cssR PE=1 SV=1 - [CSSR_BACSU]	0.00	71.23	12.89	2	2
**O34969**	Uncharacterized oxidoreductase yfjR OS=Bacillus subtilis GN=yfjR PE=3 SV=2 - [YFJR_BACSU]	0.00	59.06	11.54	2	2
**C0SP95**	Copper transport protein ycnJ OS=Bacillus subtilis GN=ycnJ PE=1 SV=1 - [YCNJ_BACSU]	0.00	47.41	4.99	2	2
**P04788**	Xylose isomerase OS=Bacillus subtilis GN=xylA PE=3 SV=3 - [XYLA_BACSU]	0.00	48.20	5.62	2	2
**P46353**	Phosphopentomutase OS=Bacillus subtilis GN=drm PE=3 SV=3 - [DEOB_BACSU]	0.00	115.67	6.35	3	2
**P17894**	DNA repair protein recN OS=Bacillus subtilis GN=recN PE=3 SV=2 - [RECN_BACSU]	0.00	65.96	4.34	2	2
**P54378**	Aminomethyltransferase OS=Bacillus subtilis GN=gcvT PE=1 SV=2 - [GCST_BACSU]	0.00	83.00	7.18	3	2
**P46347**	Putative metalloprotease yqfG OS=Bacillus subtilis GN=yqfG PE=3 SV=3 - [YQFG_BACSU]	0.00	51.87	14.65	2	2
**Q99027**	Sensor histidine kinase comP OS=Bacillus subtilis GN=comP PE=2 SV=3 - [COMP_BACSU]	0.00	42.43	2.60	2	2
**O32220**	Copper-exporting P-type ATPase A OS=Bacillus subtilis GN=copA PE=1 SV=2 - [COPA_BACSU]	0.00	50.69	2.74	3	2
**P39576**	Branched-chain-amino-acid aminotransferase 2 OS=Bacillus subtilis GN=ilvK PE=1 SV=5 - [ILVE2_BACSU]	0.00	39.15	5.23	2	2
**P54941**	Iron(3+)-hydroxamate-binding protein yxeB OS=Bacillus subtilis GN=yxeB PE=1 SV=2 - [YXEB_BACSU]	0.00	27.64	7.17	2	2
**P42962**	Uncharacterized protein ycsE OS=Bacillus subtilis GN=ycsE PE=3 SV=2 - [YCSE_BACSU]	0.00	67.24	13.25	3	2
**O32117**	NADH dehydrogenase-like protein yutJ OS=Bacillus subtilis GN=yutJ PE=3 SV=2 - [YUTJ_BACSU]	0.00	49.17	6.20	4	2
**P25811**	tRNA modification GTPase mnmE OS=Bacillus subtilis GN=mnmE PE=3 SV=1 - [MNME_BACSU]	0.00	50.33	7.63	3	3
**P22250**	Glutamyl-tRNA synthetase OS=Bacillus subtilis GN=gltX PE=3 SV=1 - [SYE_BACSU]	0.00	133.89	7.45	5	3

**P20278**	50S ribosomal protein L36 OS=Bacillus subtilis GN=rpmJ PE=3 SV=1 - [RL36_BACSU]	0.00	43.05	59.46	5	3
**P28611**	Chemotaxis protein motA OS=Bacillus subtilis GN=motA PE=3 SV=1 - [MOTA_BACSU]	0.00	117.13	15.19	4	3
**P28599**	10 kDa chaperonin OS=Bacillus subtilis GN=groS PE=1 SV=2 - [CH10_BACSU]	0.00	42.60	35.11	3	3
**P35161**	Cytochrome c biogenesis protein resB OS=Bacillus subtilis GN=resB PE=4 SV=1 - [RESB_BACSU]	0.00	44.03	6.83	4	3
**P39804**	Intracellular proteinase inhibitor OS=Bacillus subtilis GN=ipi PE=1 SV=1 - [IPI_BACSU]	0.00	40.77	27.73	3	3
**P37572**	DNA repair protein radA homolog OS=Bacillus subtilis GN=radA PE=3 SV=1 - [RADA_BACSU]	0.00	37.03	8.52	3	3
**P37573**	DNA integrity scanning protein disA OS=Bacillus subtilis GN=disA PE=2 SV=1 - [DISA_BACSU]	0.00	96.20	10.28	4	3
**P38493**	Cytidylate kinase OS=Bacillus subtilis GN=cmk PE=1 SV=1 - [KCY_BACSU]	0.00	87.35	17.41	4	3
**P42437**	Uroporphyrinogen-III C-methyltransferase OS=Bacillus subtilis GN=nasF PE=2 SV=1 - [NASF_BACSU]	0.00	82.83	7.25	7	3
**P42980**	Methylglyoxal synthase OS=Bacillus subtilis GN=mgsA PE=3 SV=1 - [MGSA_BACSU]	0.00	51.94	24.82	5	3
**P54391**	Uncharacterized protein ypiF OS=Bacillus subtilis GN=ypiF PE=4 SV=1 - [YPIF_BACSU]	0.00	77.35	27.70	5	3
**P10727**	Anti-sigma F factor antagonist OS=Bacillus subtilis GN=spoIIAA PE=1 SV=3 - [SP2AA_BACSU]	0.00	56.48	29.06	5	3
**P54173**	Uncharacterized protein ypjQ OS=Bacillus subtilis GN=ypjQ PE=1 SV=1 - [YPJQ_BACSU]	0.00	59.35	23.73	4	3
**P54550**	NADPH dehydrogenase OS=Bacillus subtilis GN=namA PE=1 SV=3 - [NAMA_BACSU]	0.00	32.61	13.31	3	3
**O06476**	Uncharacterized ABC transporter ATP-binding protein yfmR OS=Bacillus subtilis GN=yfmR PE=3 SV=1 - [YFMR_BACSU]	0.00	47.10	6.20	3	3
**O34705**	Phospholipase ytpA OS=Bacillus subtilis GN=ytpA PE=1 SV=1 - [PLBAC_BACSU]	0.00	60.60	16.60	4	3
**P40778**	UDP-N-acetylmuramate--L-alanine ligase OS=Bacillus subtilis GN=murC PE=3 SV=2 - [MURC_BACSU]	0.00	53.45	7.41	5	3
**P94463**	Methionyl-tRNA formyltransferase OS=Bacillus subtilis GN=fmt PE=3 SV=2 - [FMT_BACSU]	0.00	53.67	13.25	4	3
**O34751**	Uncharacterized protein yloV OS=Bacillus subtilis GN=yloV PE=4 SV=1 - [YLOV_BACSU]	0.00	29.97	8.32	3	3
**P80875**	General stress protein 16U OS=Bacillus subtilis GN=yceD PE=1 SV=3 - [G16U_BACSU]	0.00	97.55	19.17	3	3
**O34510**	Fe(3+)-citrate import ATP-binding protein yfmF OS=Bacillus subtilis GN=yfmF PE=3 SV=1 - [YFMF_BACSU]	0.00	45.83	12.41	3	3
**O34443**	Adenine phosphoribosyltransferase OS=Bacillus subtilis GN=apt PE=3 SV=1 - [APT_BACSU]	0.00	85.91	20.59	3	3
**O34340**	3-oxoacyl-[acyl-carrier-protein] synthase 2 OS=Bacillus subtilis GN=fabF PE=3 SV=1 - [FABF_BACSU]	0.00	145.99	8.72	5	3
**P81101**	Ribosome-recycling factor OS=Bacillus subtilis GN=frr PE=1 SV=2 - [RRF_BACSU]	0.00	76.52	18.38	6	3
**O05265**	Uncharacterized oxidoreductase yulF OS=Bacillus subtilis GN=yulF PE=3 SV=2 - [YULF_BACSU]	0.00	30.89	10.67	3	3
**P94490**	Xylose repressor OS=Bacillus subtilis GN=xylR PE=3 SV=3 - [XYLR1_BACSU]	0.00	64.66	10.94	5	3
**P42240**	Probable D-galactarate dehydratase OS=Bacillus subtilis GN=garD PE=3 SV=3 - [GARD_BACSU]	0.00	167.96	8.24	4	3
**O31775**	Uncharacterized protein ymdB OS=Bacillus subtilis GN=ymdB PE=4 SV=2 - [YMDB_BACSU]	0.00	43.15	14.02	3	3

**O31849**	Uncharacterized protein yojO OS=Bacillus subtilis GN=yojO PE=4 SV=2 - [YOJO_BACSU]	0.00	44.02	6.74	3	3
**P54381**	Glycyl-tRNA synthetase beta subunit OS=Bacillus subtilis GN=glyS PE=3 SV=2 - [SYGB_BACSU]	0.00	27.75	5.30	4	3
**P45744**	Isochorismate synthase dhbC OS=Bacillus subtilis GN=dhbC PE=1 SV=2 - [DHBC_BACSU]	0.00	66.48	11.81	5	3
**O34989**	Sensor histidine kinase yvrG OS=Bacillus subtilis GN=yvrG PE=3 SV=2 - [YVRG_BACSU]	0.00	82.65	6.03	4	3
**P42100**	Glycerate kinase OS=Bacillus subtilis GN=glxK PE=3 SV=2 - [GLXK_BACSU]	0.00	196.58	11.52	6	3
**P37469**	Replicative DNA helicase OS=Bacillus subtilis GN=dnaC PE=3 SV=2 - [DNAC_BACSU]	0.00	63.09	8.59	6	3
**O31766**	Uncharacterized zinc protease ymfH OS=Bacillus subtilis GN=ymfH PE=3 SV=2 - [YMFH_BACSU]	0.00	53.22	8.41	3	3
**P05655**	Levansucrase OS=Bacillus subtilis GN=sacB PE=1 SV=1 - [SACB_BACSU]	0.00	134.22	9.94	8	4
**P45743**	Isochorismatase OS=Bacillus subtilis GN=dhbB PE=1 SV=1 - [DHBB_BACSU]	0.00	59.34	15.06	5	4
**P50848**	Putative metalloprotease ypwA OS=Bacillus subtilis GN=ypwA PE=1 SV=1 - [YPWA_BACSU]	0.00	39.02	7.19	6	4
**O07631**	GTP-binding protein typA/bipA homolog OS=Bacillus subtilis GN=typA PE=3 SV=1 - [TYPA_BACSU]	0.00	90.20	10.13	6	4
**O34645**	Alpha-galactosidase OS=Bacillus subtilis GN=melA PE=3 SV=1 - [AGAL_BACSU]	0.00	82.52	9.26	5	4
**P54617**	Phage shock protein A homolog OS=Bacillus subtilis GN=ydjF PE=1 SV=3 - [PSPA_BACSU]	0.00	172.34	23.35	6	4
**O31646**	Mannose-6-phosphate isomerase manA OS=Bacillus subtilis GN=manA PE=3 SV=1 - [MANA1_BACSU]	0.00	124.42	16.83	8	4
**O31778**	(Dimethylallyl)adenosine tRNA methylthiotransferase miaB OS=Bacillus subtilis GN=miaB PE=3 SV=1 - [MIAB_BACSU]	0.00	124.23	8.64	6	4
**P80860**	Glucose-6-phosphate isomerase OS=Bacillus subtilis GN=pgi PE=1 SV=4 - [G6PI_BACSU]	0.00	113.14	11.56	10	4
**P54547**	Glucose-6-phosphate 1-dehydrogenase OS=Bacillus subtilis GN=zwf PE=1 SV=2 - [G6PD_BACSU]	0.00	141.36	9.20	11	4
**P24141**	Oligopeptide-binding protein oppA OS=Bacillus subtilis GN=oppA PE=1 SV=1 - [OPPA_BACSU]	0.00	146.12	9.36	8	5
**P50743**	GTP-binding protein engA OS=Bacillus subtilis GN=engA PE=1 SV=1 - [ENGA_BACSU]	0.00	130.50	13.30	5	5
**O07621**	Heme-based aerotactic transducer hemAT OS=Bacillus subtilis GN=hemAT PE=1 SV=1 - [HEMAT_BACSU]	0.00	218.88	14.81	11	5
**O35002**	Carboxy-terminal processing protease ctpB OS=Bacillus subtilis GN=ctpB PE=1 SV=1 - [CTPB_BACSU]	0.00	103.75	13.33	8	5
**P54572**	Probable NAD-dependent malic enzyme 1 OS=Bacillus subtilis GN=yqkJ PE=3 SV=1 - [MAO1_BACSU]	0.00	261.75	18.68	11	6
**P55179**	Peptidase T OS=Bacillus subtilis GN=pepT PE=3 SV=1 - [PEPT_BACSU]	0.00	174.92	18.29	7	6
**O34824**	Phosphoglucosamine mutase OS=Bacillus subtilis GN=glmM PE=1 SV=1 - [GLMM_BACSU]	0.00	292.51	18.08	15	6
**P07343**	Fumarate hydratase class II OS=Bacillus subtilis GN=fumC PE=3 SV=2 - [FUMC_BACSU]	0.00	404.62	13.85	27	6
**P54542**	Uncharacterized protein yqjE OS=Bacillus subtilis GN=yqjE PE=3 SV=2 - [YQJE_BACSU]	0.00	218.01	21.83	7	6
**O05239**	Probable NADH-dependent butanol dehydrogenase 1 OS=Bacillus subtilis GN=yugJ PE=3 SV=1 - [YUGJ_BACSU]	0.00	217.36	23.51	8	7
**O32039**	Histidyl-tRNA synthetase OS=Bacillus subtilis GN=hisS PE=3 SV=1 - [SYH_BACSU]	0.00	147.17	17.92	9	7

**P39142**	Pyrimidine-nucleoside phosphorylase OS=Bacillus subtilis GN=pdp PE=3 SV=2 - [PDP_BACSU]	0.00	207.21	14.32	12	7
**P37518**	GTP-dependent nucleic acid-binding protein engD OS=Bacillus subtilis GN=engD PE=3 SV=1 - [ENGD_BACSU]	0.00	512.11	33.06	22	9
**P12425**	Glutamine synthetase OS=Bacillus subtilis GN=glnA PE=1 SV=3 - [GLNA_BACSU]	0.00	466.41	25.68	19	10
**Q05873**	Valyl-tRNA synthetase OS=Bacillus subtilis GN=valS PE=1 SV=3 - [SYV_BACSU]	0.00	144.70	12.84	15	10
**P80859**	6-phosphogluconate dehydrogenase, decarboxylating 2 OS=Bacillus subtilis GN=yqjI PE=1 SV=4 - [6PGD2_BACSU]	0.00	473.58	34.75	29	13
